# Redefining the Symphony of Light Aromatic Synthesis Beyond Fossil Fuels: A Journey Navigating through a Fe-Based/HZSM-5 Tandem Route for Syngas Conversion

**DOI:** 10.1021/acscatal.4c03941

**Published:** 2024-10-01

**Authors:** Muhammad Asif Nawaz, Rubén Blay-Roger, Maria Saif, Fanhui Meng, Luis F. Bobadilla, Tomas Ramirez Reina, J. A. Odriozola

**Affiliations:** † Department of Inorganic Chemistry and Materials Sciences Institute, University of Seville-CSIC, 41092 Seville, Spain; ‡ State Key Laboratory of Clean and Efficient Coal Utilization, College of Chemical Engineering and Technology, Taiyuan University of Technology, Taiyuan 030024, China; § School of Chemistry and Chemical Engineering, University of Surrey, Guildford GU2 7XH, U.K.

**Keywords:** syngas, CO_2_ valorization, Fischer−Tropsch synthesis, HZSM-5 zeolite, aromatics

## Abstract

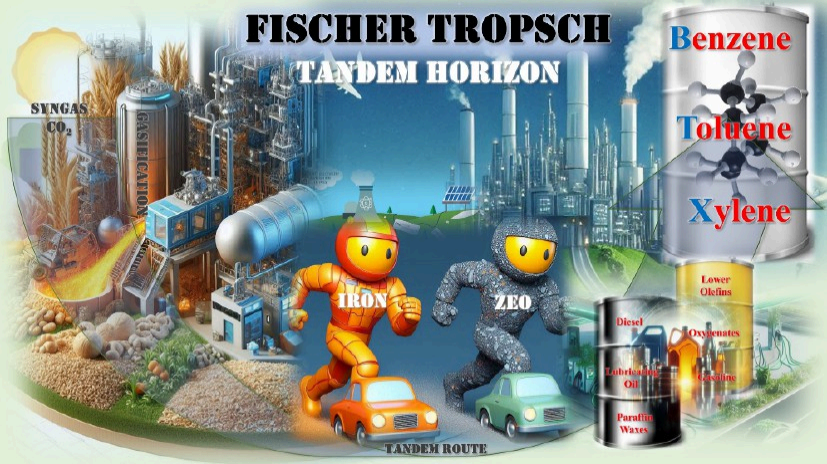

The escalating concerns about traditional reliance on fossil fuels and environmental issues associated with their exploitation have spurred efforts to explore eco-friendly alternative processes. Since then, in an era where the imperative for renewable practices is paramount, the aromatic synthesis industry has embarked on a journey to diversify its feedstock portfolio, offering a transformative pathway toward carbon neutrality stewardship. This Review delves into the dynamic landscape of aromatic synthesis, elucidating the pivotal role of renewable resources through syngas/CO_2_ utilization in reshaping the industry’s net-zero carbon narrative. Through a meticulous examination of recent advancements, the current Review navigates the trajectory toward admissible aromatics production, highlighting the emergence of Fischer–Tropsch tandem catalysis as a game-changing approach. Scrutinizing the meliorated interplay of Fe-based catalysts and HZSM-5 molecular sieves would uncover the revolutionary potential of rationale design and optimization of integrated catalytic systems in driving the conversion of syngas/CO_2_ into aromatic hydrocarbons (especially BTX). In essence, the current Review would illuminate the path toward cutting-edge research through in-depth analysis of the transformative power of tandem catalysis and its capacity to propel carbon neutrality goals by unraveling the complexities of renewable aromatic synthesis and paving the way for a carbon-neutral and resilient tomorrow.

## 1 Introduction

In the dynamic landscape of the modern petrochemical era, the pursuit of energy has propelled a rapid surge in unlocking the potential of aromatic monomers.^1^ These remarkable building blocks now stand at the forefront, driving the creation of a myriad of high-value chemicals and transforming industries with their boundless potential.^2,3^ Concurrently, within the realm of aromatic synthesis, traditional methods have historically been guided by established technical routes sourced from vast petroleum resources. Yet, as industries continue to innovate and expand, the demand for aromatic hydrocarbons is expected to grow, thus necessitating a careful balance between meeting industrial needs and addressing environmental sustainability concerns.^4−6^

The prominent strategies in traditional significant routes of aromatic hydrocarbon synthesis depend on the bulk feed sources derived from intensive conventional carbon routes, involving complex processes of reforming and cracking, etc.^7−10^ As the world gravitates toward sustainable practices, advancements in the synthesis of aromatic hydrocarbons through nonpetroleum routes have gained momentum.^11^ In the present technological and economic era, there is a significant appeal in obtaining biobased low-carbon olefins as fundamental building blocks for the modern aromatic industry serving the energy, aerospace, and defense sectors. Utilizing biobased carbon alternatives or implementing waste recycling processes for olefin raw materials presents an opportunity for accessing a renewable and potentially carbon-neutral source. This strategic shift has the potential to transition the fossil fuel-reliant society toward more adaptable scenarios in the realm of future biofuels, holding promise for the decarbonization of transportation and advancing toward an emission-free society.^8,12^ Therefore, the acceleration of research and development in this realm signifies a pivotal moment in the energy landscape, where innovation and sustainability converge to shape a more environmentally conscious and resilient future.^13,14^ Several approaches focus on utilizing renewable feedstocks and alternative raw materials to produce aromatic compounds from nonpetroleum resources as part of efforts to develop sustainable and environmentally friendly processes.^15−21^ Regarding this, tandem catalysis offers a forward-thinking strategy to augment efficiency and selectivity in the pivotal chemical reactions vital for liquid fuel generation while integrating two separate components (metal-based oxides and the hydrocracking phase) within a unified process. It typically orchestrates a series of reactions, where the output of one reaction seamlessly becomes the input for the next, thereby enabling the utilization of intermediates without necessitating their isolation.^22−26^

Here, the selection of the initial catalyst is intimately tied to the operational parameters, specific sites, and functionalities of the secondary catalyst, predominantly zeolite during the hydrocracking (HCR) phase. The advancements in the integration of metal-based catalysts via Fischer–Tropsch synthesis (FTS),^20,27−32^ reverse water gas shift (RWGS)-FTS,^19,33−40^ or methanol synthesis (MTS)^41−43^ catalytic routes integrated with HZSM-5 (as an HCR or upgrading phase) for the direct synthesis of aromatic hydrocarbons from syngas and CO_2_ represent significant strides in sustainable catalysis. These systems demonstrate enhanced catalytic activity and selectivity in the conversion of syngas/CO_2_ into valuable aromatic hydrocarbons and address the key development in optimizing reaction conditions, exploring different promoters, bimetallic synergies, and the shape-selective confinement of HZSM-5 for improved efficiency and addressing catalyst stability either by FTS or MTS route. However, addressing challenges in product selectivity and striving for advancements using composite materials for tandem catalysis underscore the importance of cooperative adaptability in cascading reactions. In this sense, iron-based catalysts, renowned for their thermal resilience and compatibility with elevated operating temperatures (>300 °C, akin to those of the HCR phase), intrinsic conversion levels for inherent WGS/RWGS activity, and adaptability to various feed gas compositions (H_2_/CO/CO_2_), could compete well with conventional contenders of Ru/Co- or methanol-mediated systems.^19,20,27−40,44^ However, in particular, gathering interest toward benzene, toluene, and xylene (BTX) selectivity via Fe-based integrated catalysts entails detailed mechanistic insights, and differences in the bifunctionalities of MTS, FTS, RWGS-FTS, and RWGS-FTS+HCR catalytic routes deliberately need to be prioritized in view of recent advancement and reports. Therefore, a comprehensive report redefining the symphony of light aromatics (BTX) beyond fossil fuels by exploring advanced catalyst structures that allow reactivity adjustment in tandem catalysis through deliberate designs could address the optimization of CO_2_-enriched syngas systems.

This Review showcases the narrative of the carbon neutrality goal, which has emerged as a substantial shift in recent studies for the manufacturing of aromatic hydrocarbons through syngas/CO_2_ valorization by integrated catalytic systems. It deeply explores the transformative potential of the FTS tandem approach, shedding light on its recent developments and the promise it holds for a cleaner and greener energy landscape. It further delves into the detailed mechanistic approaches and advanced catalytic systems that pave the way toward the revolutionary role of Fe-based/HZSM-5 integrated catalysts through biobased syngas scenarios.

## 2 Aromatics Industry

Aromatic hydrocarbons are a class of organic compounds characterized by the presence of one or more aromatic rings, which are planar cyclic structures with alternating single and double bonds. They generally refer to a class of hydrocarbons containing a benzene ring structure and mainly include monocyclic aromatic hydrocarbons such as benzene, toluene, and xylene (BTX); polycyclic aromatic hydrocarbons such as biphenyl, polyphenylene, etc.; and fused-ring aromatic hydrocarbons such as naphthalene and anthracene.^4,5^

**Figure 1:**
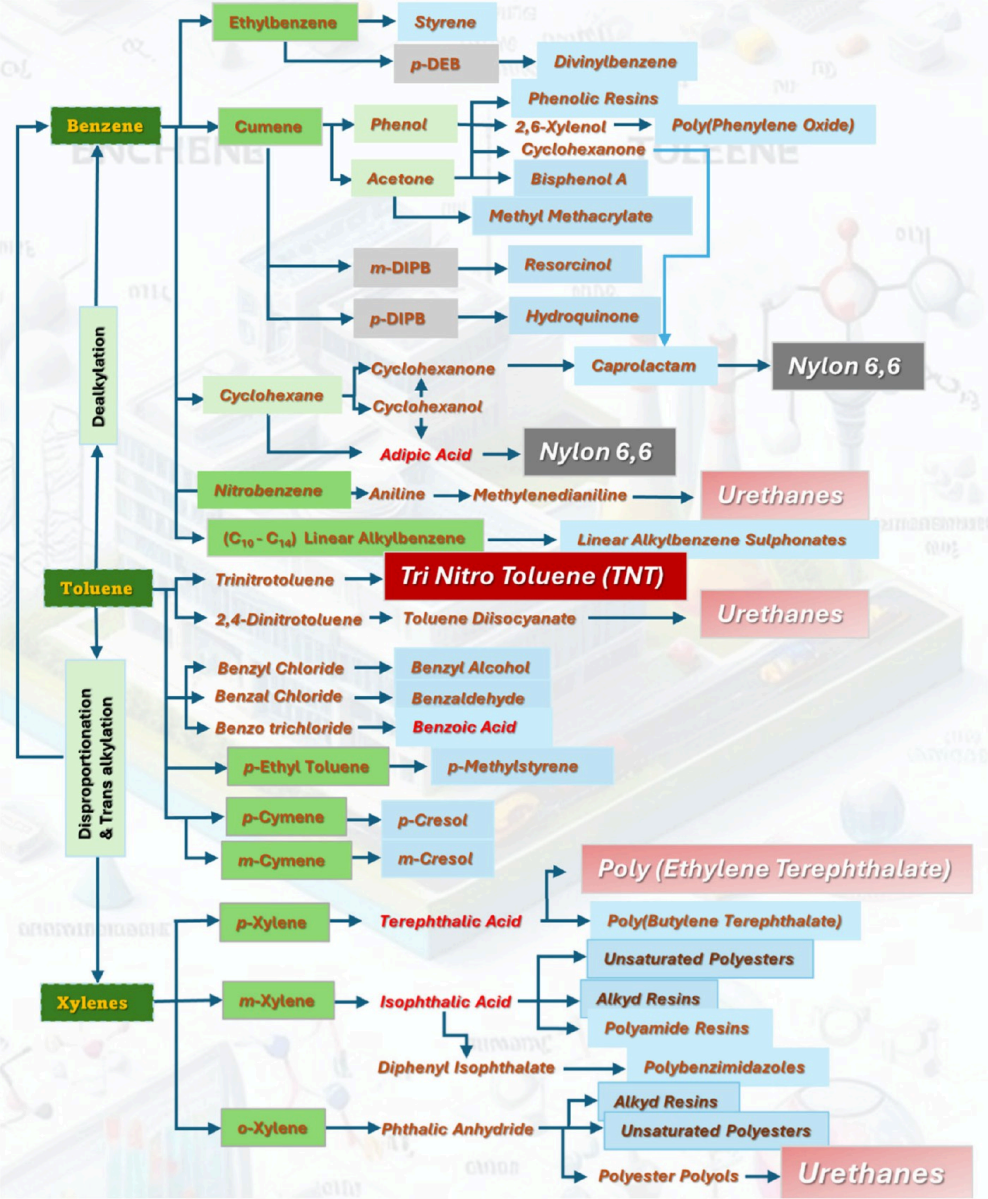
Application of BTX in various fields derived through multiple steps of isomerization, disproportionation, and *trans*-alkylation. Reproduced with permission from ref (45). Copyright 1999 Elsevier.

Among these, the single-ring aromatic hydrocarbons are divided into heavy and light aromatic hydrocarbons. Heavy aromatic hydrocarbons refer to a subgroup of aromatic hydrocarbons that have higher molecular weights and larger structures compared to their lighter counterparts, where the term “heavy aromatic hydrocarbons” can be used broadly, and the specific compounds included in this category may vary depending on the context of use. Additionally, heavy aromatic hydrocarbons may undergo various chemical transformations and reactions, contributing to the complexity of their environmental and industrial impact. Meanwhile, light aromatic hydrocarbons, including benzene, toluene, and xylene (often collectively referred to BTX), are a group of organic compounds characterized by the presence of one or more benzene rings. Due to their unique chemical properties and versatile reactivity, the bedrock of modern chemicals is enriched with BTX derivatives, which serve as essential building blocks for an extensive array of compounds everyday life (Figure 1).^45^ The increasing demand for aromatic hydrocarbons reflects their indispensable role in numerous industrial sectors, driving a surge in global consumption, where aromatic hydrocarbons such as benzene, toluene, and xylene serve as vital building blocks for the synthesis of a diverse array of products, including plastics, resins, fibers, pharmaceuticals, and solvents. The escalating demand is particularly pronounced in the polymer industry, where aromatic hydrocarbons contribute to the production of essential materials such as polyethylene terephthalate (PET) and styrene-based polymers. The expanding automotive and packaging industries further contribute to the surge, as aromatic hydrocarbons play pivotal roles in the manufacturing of synthetic rubbers, adhesives, and coatings. Additionally, the growing need for chemicals in everyday products and the pharmaceutical sector underscores the versatility and ubiquity of aromatic hydrocarbons in modern industrial applications. The automotive industry mainly relies upon aromatic hydrocarbons, where BTX are used as fuel additives to enhance the octane rating of gasoline, improving the combustion efficiency in internal combustion engines. They are the crucial elements in the production of styrene, a monomer in the synthesis of synthetic rubber, styrene–butadiene rubber (SBR). They are also consumer goods and personal care products in the form of perfumes, cosmetics, and personal care products. Benzene is chlorinated to produce chlorobenzenes, which are used in the production of herbicides, insecticides, and fungicides. Benzene serves as a precursor in the production of adipic acid, which is a key intermediate in the synthesis of nylon fibers. It is also used in the synthesis of phenolic resins, which are important in the production of adhesives, coatings, and molded products.

Benzene can also be alkylated to linear alkylbenzenes (laboratories), which are used in the production of detergents and surfactants. Aniline, produced from benzene, is a key building block for the synthesis of various agrochemicals. Benzene can also be hydrogenated to produce cyclohexane, a precursor in the production of nylon and a solvent in various chemical processes, while caprolactam, used in the production of nylon-6, is synthesized from cyclohexane derived from benzene. The high-value-added pyromellitic toluene, with pyromellitic dianhydride as a downstream product, is mainly used in the production of new high-temperature-resistant and insulating materials such as polyimide (PI), where its excellent engineering properties encourage its wider utilization in defense, aviation, machinery, electronics, atomic energy, and other industries.^2,46,47^ In contrast, toluene is adopted as a solvent in industrial cleaning processes, particularly for degreasing and removing adhesives. *para*-Xylene is a key feedstock in the production of terephthalic acid, which is a crucial intermediate in the synthesis of PET. *ortho*-Xylene is utilized in the production of isophthalic acid, a key component in the synthesis of resins and plasticizers. Toluene and xylene are used in the production of toluene diisocyanate, a key component in the synthesis of polyurethane foam and coatings, and as solvents in certain chromatography techniques, analytical chemistry, and the printing industry to facilitate the fluidity and drying properties of the ink. BTX are the starting materials to produce phenol, a compound used in the synthesis of pharmaceuticals, resins, and other chemicals,^48^ where toluene is adopted in producing benzoic acid and toluene-sulfonamide compounds, which are backbone compounds in the pharmaceutical industry. Toluene is oxidized to produce benzaldehyde, which is used in the synthesis of flavors, fragrances, and pharmaceuticals and can be chlorinated to produce benzyl chloride, an intermediate in the synthesis of benzyl compounds and pharmaceuticals. Toluene is commonly used as a solvent in Grignard reactions, facilitating the formation of various organic compounds. Nitration of benzene leads to the production of nitrobenzene, which serves as an intermediate in the synthesis of dyes and explosives. Additionally, toluene is a precursor in the synthesis of trinitrotoluene (TNT), an explosive widely used in military and industrial applications.

The broad range of applications showcases the versatility of aromatic hydrocarbons in different industries, making them indispensable in the production of numerous materials, chemicals, and consumer goods. However, it is important to note that aromatic hydrocarbons can have health and safety considerations, and their handling is subject to proper safety precautions and regulations, where efforts are ongoing to explore alternative, environmentally friendly processes and feedstocks in certain applications.

### 2.1 Aromatics Production Technologies

#### 2.1.1 Potential of Traditional Routes and the Associated Challenges in the Long-Term Practices

**Figure 2:**
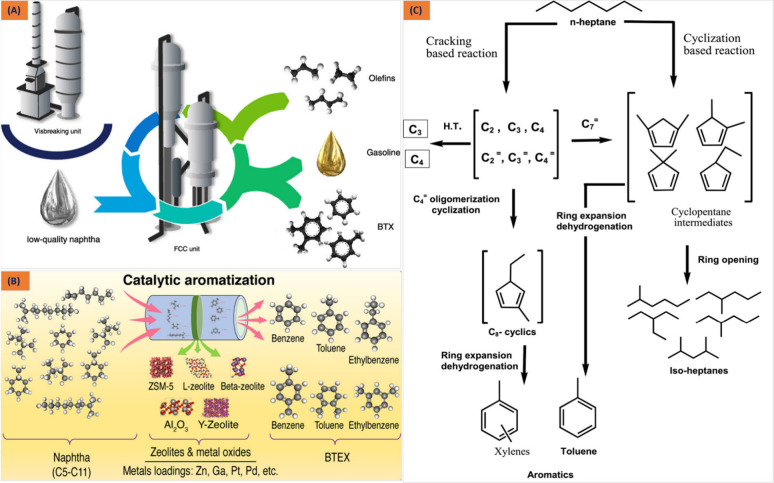
(A) Naphtha fluid catalytic cracking unit. Reproduced with permission from ref (56). Copyright 2020 American Chemical Society. (B, C) Reaction Pathways for the aromatization process of naphtha and n-C_7_ over a ZSM-5 catalyst. Reproduced with permission from ref (57). Copyright 2023 American Chemical Society. Reproduced with permission from (51). Copyright 2022 Elsevier.

The synthesis of aromatic hydrocarbons through the petroleum route stands as an enduring cornerstone in the petrochemical industry, representing a fundamental process that has shaped modern chemistry and industrial production. In the intricate dance of hydrocarbon transformations, catalytic cracking and reforming of naphtha emerge as transformative steps in the synthesis of aromatic hydrocarbons. This conventional method leverages crude oil, a complex mixture of hydrocarbons extracted from geological reservoirs, as the primary feedstock. Through a series of refining steps, including distillation, cracking, and reforming, the heavier fractions of crude oil are transformed into the iconic trio of benzene, toluene, and xylene. At present, the petroleum route has not only fueled the growth of diverse industries but also played a pivotal role in shaping the global economy, remaining integral to the petrochemical landscape and exemplifying the industry’s historical resilience and adaptability. At the heart of aromatic hydrocarbon synthesis lies crude oil, a complex amalgamation of hydrocarbons extracted from the Earth’s crust. Naphtha, a crucial component in refineries obtained from crude oil (containing C_5_–C_11_) hydrocarbons with a boiling range of 30–200 °C, can be further processed into light and heavy categories based on boiling points (30–90 °C and 90–200 °C, respectively). Light or heavy naphtha is directly obtained from refinery distillation, whereas reformate naphtha is crafted through catalytic reforming and cracked naphtha emerges via processes like fluid catalytic cracking (FCC) and hydrocracking. The composition of naphtha, encompassing *n*- and *i*-paraffins, olefins, naphthenes, and aromatics, is subject to variations based on the crude type and processing methods. Notably, naphtha rich in naphthenes yields a higher abundance of BTX (benzene, toluene, and xylene). Therefore, it is in the heavier fractions that the rich tapestry of aromatic compounds begins to unfold. The journey takes a transformative turn with catalytic cracking, a process designed to break down large hydrocarbons into smaller, more valuable components.^49^ Zeolite catalysts, often in the form of finely powdered molecular sieves, play a pivotal role, where they function as molecular gatekeepers, guiding the cracking reactions to produce an assortment of hydrocarbons (Figure 2A). Table 1 outlines significant processes for converting light hydrocarbons such as liquefied petroleum gas (LPG) and light naphtha into aromatics. Notably, Aromax specifically targets benzene production from *n*-C_6_ by utilizing a specialized zeolite in a conventional fixed-bed reforming setup. Presently, catalytic naphtha reforming and naphtha steam cracking (pyrolysis of gasoline) units serve as primary global sources of BTX aromatics. In certain oil-to-chemical refineries, aromatic complexes are generated via high-severity catalytic reforming, where benzene precursors remain unseparated from naphtha. Here, catalytic reforming could be likened to the art of restructuring, with the reforming steps unfurling onto the stage of a pivotal process shaping the trajectory of aromatic hydrocarbon synthesis. It is a process that transforms saturated (naphthenes and paraffins) hydrocarbons into aromatics, involving γ-alumina modified with different chlorinating agents and impregnated by numerous metal species^50,51^ (Figure 2B and C). The high aromatic selectivity of continuous reforming technology has made it a widely adopted and relatively mature process, such as the CCR Platformer process of UP Company.^52^ Under the influence of metal-based catalysts, typically containing platinum or rhenium, the molecular architecture undergoes a transformative shift, where benzene, toluene, and xylene emerge as the stars of the show. However, the consecutive advancement of the petrochemical market and the many variational factors in the petroleum industry have brought various challenges and opportunities to this era.

**Table 1:** Aromatization Processes Adopted in the Conversion of Light Hydrocarbons

company	process	feed	catalyst
UP	Cyclar^a^	LPG	Ga/ZSM-5
GTC Technology	GT-Aromatization^a^	olefins	zeolite
ExxonMobil	M2 forming	naphtha	ZSM-5
Chevron-Phillips Chemical	Aromax^a^	*n*-C_6_	Pt/K(Ba) L
Asahi Chemical	Alpha^a^	olefins	Zn/ZSM-5
ENEOS	Z-forming	LPG	Ga/ZSM-5

a Commercial process.

Traditional aromatic hydrocarbon synthesis methods heavily reliant on fossil fuels as primary feedstocks present significant challenges, contributing to environmental degradation and resource depletion. The extraction and processing of these fossil fuels such as crude oil or natural gas are associated with extensive energy consumption and greenhouse gas emissions.^53^ In addition to the reforming process, the follow-up process also includes the extraction of aromatics, which is a necessary separation step in production.^54^ Here, the development of solvents is the key link in the extraction of aromatic hydrocarbons, in which sulfolane has found its own way as ab extraction agent with the advantages of being a cheap, easy to obtain, nontoxic, and harmless substance.^55^ However, these conventional processes perpetuate a dependence on finite and nonrenewable resources, leading to concerns over long-term sustainability. The imperative for a transition to greener and more sustainable alternatives is evident, thus necessitating the exploration of innovative technologies and feedstocks that reduce the environmental footprint and align with the principles of circular economy and green chemistry. Efforts to diversify feedstocks, enhance energy efficiency, and reduce the environmental impact of aromatic hydrocarbon synthesis are crucial to building a more sustainable and resilient chemical industry. Growing awareness of climate change and environmental sustainability is shifting the focus toward greener alternatives in chemical production, which could weave a narrative that intertwines technological innovation and economic significance. Therefore, in a world increasingly attuned to sustainability, the synthesis of aromatic hydrocarbons through nonpetroleum routes emerges as a transformative chapter, where the industry’s ability to address those challenges will determine its resilience and viability in holding the promise of resource conservation and the integration of renewable energy and a circular economy.

### 2.2 Alternative Strategies as a Renewable Canvas Beyond Fossil Fuels

At present, various Chinese and multinational companies, including Hainan Petrochemical, Zhejiang Petrochemical, Hengyi, and Rongsheng groups of industries, are all expanding the production capacity of aromatics. This capacity is mainly aligned with the refining and chemical integration project, and the required crude oil or naphtha raw materials still need to be imported in large quantities.^6^ However, one of the most promising avenues for alternative energy sources involves the widespread adoption of sustainable synthetic fuels. These fuels have the potential to align with international environmental policies, offering a viable solution to address concerns related to fossil fuels. Among these innovative options, syngas, a dynamic blend of CO and H_2_ derived from diverse sources such as carbon(IV) oxide, biomass, coal, natural gas, and carbon-based waste, shines brightly. It not only serves as a versatile source of chemical feedstocks and oxygenates but also promises to supplant traditional fuels derived from crude oil. In the face of the depletion of conventional resources and soaring market demands for oxygenate precursors, the synthesis of aromatic hydrocarbons is undergoing a transformative shift. Here, biomass- or coal-based syngas processes, with their variable H_2_/CO ratios, offer compelling alternatives to conventional routes.^2,58,59^ The emergence of coal to liquid technology (CtL) has now been industrialized, leading to the efficient formulation of aromatic hydrocarbons, with a series of advanced techniques being adopted in past years.^60^ However, as we confront the environmental repercussions of escalating CO_2_ emissions inherent in hydrocarbon generation, urgent measures to mitigate these impacts have come to the fore. Thus, the imperative to combat the proliferation of carbon dioxide, the primary anthropogenic contributor to global warming, has spurred the development of innovative strategies aimed at alleviating these environmental burdens.^4,61^ Therefore, the nonpetroleum route of producing aromatics from biomass could be in line with global resource distribution and natural conditions by recycling waste CO_2_ streams along with feed gas, contributing toward the need for global raw materials to a certain extent. Given the imperative role of energy security as a “dwelling needle”, particularly in the era of “double carbon”, efficiently and cleanly converting biomass resources into chemicals becomes a paramount challenge and demand in the energy and resources sectors. At present, the technical route of producing aromatic hydrocarbons from biomass as the raw material reduces the investment and operating cost of equipment with improved production efficiency and efficient development prospects.^62^ Biomass-to-aromatics (BTA) can be introduced as a process of gasifying or pyrolyzing biomass to obtain syngas, then syngas could be used as raw material to prepare aromatics. Utilizing biomass-derived feedstocks from agricultural and forestry residues or processing municipal solid waste could provide a renewable and sustainable alternative, offering a carbon-neutral or even carbon-negative approach. The spotlight then shifts to syngas (a versatile mixture of hydrogen and carbon monoxide derived from these renewable sources), and converting syngas into a spectrum of hydrocarbons would be a part of an eco-friendly and circular approach.

Syngas can be converted to a wide spectrum of hydrocarbon species, typically through the coupling of a series of catalytic reactions occurring on different catalyst surfaces composed of a metal catalyst and a molecular sieve. The acidic zeolites (Lewis/Brønsted acidic strength) with hydrocracking and isomerization capabilities are the best option for typical utilization with metal oxides to successfully enhance the selectivity for liquid hydrocarbons and oxygenates by exerting control over the reaction products from the metal catalysts. Aromatics from syngas can be divided into two main catalytic categories of methanol synthesis (MTS) route and Fischer–Tropsch synthesis (FTS) route. Here, syngeas produces methanol usually with a copper- or Zn-based catalyst in the MTS route, followed by th e dehydration of to form DME at the molecular sieve. Methanol/DME and water are then converted into low-carbon olefins, and finally the low-carbon olefins are reacted by polymerization, cyclization, alkylation, or hydrogen transfer to form aromatic hydrocarbons. Meanwhile, the FTS route generally includes the generation of FTS intermediate products (olefins/paraffins) at iron/cobalt catalysts in first step, which aer subjected to the polymerization, cyclization, alkylation, isomerization, and dehydrogenation at the molecular sieve surface to form the desired aromatic hydrocarbon in the second step. The catalytic process of generating aromatics either through MTS or FTS is further categorized as the two-stage (indirect) process and the one-stage (direct, bifunctional, or tandem) approach, which typically depends upon the reactor and catalyst bed configuration.

### 2.3 Two-Stage or Indirect Production of Aromatics

**Figure 3:**
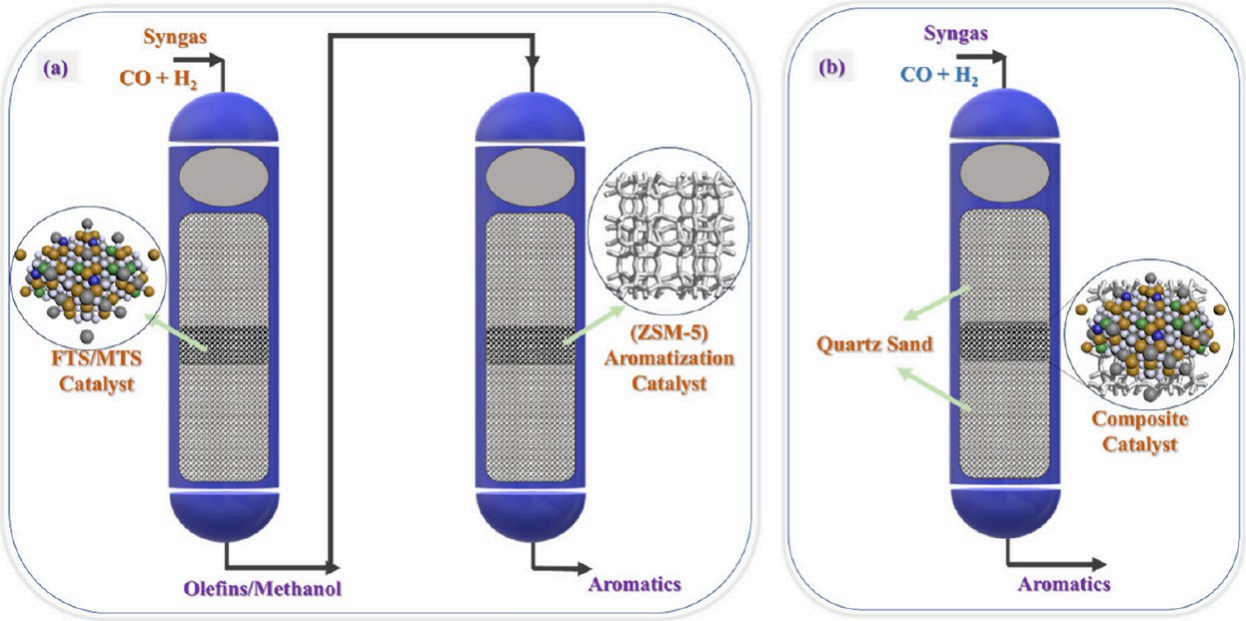
Process diagrams of the (a) two-step method and (b) one-step method for the conversion of syngas to aromatics.

“Two-stage method” refers to the use of two-stage reactors (dual bed configuration) separately, where the specific process flow is to fill the metal oxide catalysts and HZSM-5 molecular sieves in different reactors in series. The process starts with the conversion of synthesis gas into intermediate products (methanol or olefins) first in one reactor confronting the metal oxide catalyst, followed by the generation of aromatic hydrocarbons by a cracking and aromatization reaction in a second reactor containing zeolite as the molecular sieve catalyst (shown in Figure 3a).^63,64^ At present, some countries such as Brazil, South Africa, and China are rich in coal resources and developing aromatics production through coal hydroliquefaction technology and methanol to aromatics technology (MTA).^65,66^ In coal hydroliquefaction, coal is hydrolyzed into a liquid hydrocarbon mixture, followed by the extraction of naphtha fraction and then catalytically reformation of the obtained naphtha to aromatic products.^60^ In contrast, in general MTA is also a representative of the indirect production of aromatics from synthesis gas,^67,68^ in which synthesis gas is first produced by coal gasification technology, then methanol is generated from the synthesis gas through the methanol synthesis process, and finally the aromatics are obtained by the methanol-to-aromatics (MTA) process.^10,69−71^ Various studies have been reported on the production of aromatic hydrocarbons by the “two-stage method”, where Corsaro et al. filled the first stage with Co-based oxide catalysts to control a lower temperature (190 °C), and the second-stage reactor was filled with a HZSM-5 molecular sieve catalyst, adopting a controlled reaction temperature (>250 °C).^72^ Similarly, Zhang et al.^73^ used CuZnAl and γ-Al_2_O_3_ in the first-stage reactor, exploring the optimal temperature of 300 °C for the nonmethanol synthesis route, where the higher reaction temperature was mainly conducive to the dehydration reaction for direct transformation into DME. The second-stage reactor was filled with Mo-HZSM-5 molecular sieves, with a higher reaction temperature of 360 °C for controlled aromatization. Meanwhile, aromatic hydrocarbons can also be extracted as a separate stream of high-quality byproducts in a coproduction process of catalytic pyrolysis (through pyrolytic or bio-oil) by one-stage or a two-stage reactor configuration.^9,21,74^ In a study conducted by Mullen et al.,^75^ various iron-modified HZSM-5 catalysts with different iron loadings were tested for their efficacy in generating aromatic hydrocarbons. This assessment utilized a microscale pyrolysis reactor to conduct catalytic pyrolysis on cellulose, cellobiose, lignin, and switchgrass. The findings revealed that the catalyst containing 1.4 wt % Fe demonstrated the most significant enhancement in aromatic hydrocarbon production from cellulose, cellobiose, and lignin, achieving a carbon yield of approximately 18% from cellulose and that of 25% from cellobiose for selected aromatics. However, the primary obstacle with this method lies in the significant presence of oxygenated hydrocarbons produced through the catalytic pyrolysis of organic matter from sustainable sources. This requires the adoption of energy densification techniques to produce a liquid with a high energy content, as well as other fuel enhancement processes to address the unsuitability of the resulting product for direct utilization.^76^ Even though the dual-bed configuration is prioritized sometimes in terms of each bed being optimized for its specific function, providing flexibility in the reaction conditions (such as temperature, pressure, etc.) and catalyst choices being carried out separately to achieve the optimal conditions, it may also contribute toward some disadvantages of complicated steps, high equipment investment costs, and the loss of synergy between the metal oxide catalyst and HZSM-5 molecular sieves, with the lower CO conversion and selectivity for aromatics. Therefore, the choice between these methods depends on factors such as feedstock composition, desired product selectivity, and overall process economics, and the aim is to maximize the desired valuable product.

### 2.4 A Dynamic Duo of Efficiency and Stability in the Tandem Approach as Direct Route for STA Process

Coupling of multiple reactions by designing a bifunctional catalyst is a useful strategy of converting syngas into a wide spectrum of hydrocarbons species. This adaptability hinges upon the efficient separation of two critical stages transpiring on distinct active centers: first, the conversion of synthesis gas into active intermediate molecules on the surface of the metal oxide catalyst and second, the subsequent arrangement of these intermediates within the “cage or pore channels” of zeolite, guiding the selectivity of subsequent reactions such as hydrocracking, isomerization, or other transformations to manipulate the final product. Therefore, the development of such a catalytic approach that leverage properties from both zeolites and active metal catalysts has expanded the scope of zeolite applications in the rapidly growing catalytic applications of syngas conversion to liquid fuels, successfully enhancing the hydrocarbon selectivity through diverse pathways using metal–zeolite composite catalysis Therefore, theoretically combining metal oxides with a molecular sieve is a method for the direct production of aromatics from syngas (as shown in Figure 3b).^22−26^ It is evident that the characteristics of zeolites exert control over the reaction products from the metal-based oxides, serving as an intermediary phase for secondary processing, providing anchorage for certain metallic species, and enhancing selectivity toward the desired products during syngas conversion.^64,77,78^ Here, metal catalysts synergize with the acidic and shape-selective nature of HZSM-5 zeolite, creating a catalytic powerhouse that propels the conversion of biomass-derived syngas or CO_2_ into aromatic hydrocarbons. Advancements in the integration of metal-based catalysts with HZSM-5 extend beyond reactivity to encompass efficiency and stability. Given the resource landscape of recent decades and the significant market demand, the active exploration of sustainable sources for producing aromatic hydrocarbons has become a prominent subject. This involves aiming for reduced production costs, streamlining reaction processes, and enhancing the overall reaction efficiency.^79−81^ Researchers have fine-tuned catalyst formulations to enhance catalytic activity, reduce energy inputs, and increase the lifespan of the catalyst. Since the presentation of the process decades back by Chang et al.^82^ in the late 1970s demonstrating the process route of directly producing aromatics from syngas, this process turned to the effective advancement and strategies while reducing the manufacturing cost and thermodynamic limitations of the traditional indirect two-step route.^82,83^ Various modifications have been served in view of different promoters to modulate the active sites and generate the new functions of metal oxide catalyst while enhancing the anticipated aromatics fraction from that of the typical hydrocarbon stream.^84^

Here, the one-step method with the advantages of few steps, low equipment investment cost, and synergistic effect between the metal oxide catalyst and HZSM-5 molecular sieve can also greatly improve the CO conversion rate and aromatics selectivity. The metal oxide catalyst dictates the type of intermediate and the rate of formation, while the molecular sieve’s size, structure, and environmental properties determine the final product. By modulating the oxides and molecular sieves, such as their composition and structure, and optimizing the coupling between the two, precise control over product composition can be achieved. Successful industrialization of this achievement could revolutionize the coal chemical industry, offering a new paradigm for sustainable energy production. Therefore, tandem catalysis via an FTS/MTS route can be a promising way of directly synthesizing aromatics from syngas while simultaneously reducing the concerned issues. At present, the “one-step method” is mainly endorsed by most research activities for aromatics synthesis; however, a series of cascade reactions in the same reactor requires the good coupling of various parameters of the two catalysts. Here, the temperature inconsistency of the binary catalysts in the coupling process is more prominent; thuk, solving the temperature matching issue has gradually become the main interest in recent years.^4,85^ Meanwhile, the direct scheme is still in the laboratory research stage, with plenty of unique reaction mechanisms being presently proposed in this regard. The research also mainly focuses on the modification of molecular sieves, elaborative screening of the metal oxides, and the combined effects of bifunctional catalytic system on the distribution of aromatics, along with elucidating the impact of different catalytic/process parameters.^25,86−88^ Therefore, obtaining a tandem catalyst with high activity, high selectivity, and good stability is of great significance for exploring those impacts.

### 2.5 Catalytic Marvels in the Selectivity and Yield of Aromatics

The nonpetroleum route brings forth a cast of catalysts, including zeolites and various metal-based catalysts, showcasing their efficiency in steering the conversion of biomass-derived or syngas-derived intermediates into aromatic hydrocarbons. These catalysts play a crucial role in ensuring high yields, selectivity, and process efficiency in an environmentally conscious manner. Innovations in catalysis contribute to more energy-efficient and selective processes, while tailoring catalysts for biomass-derived syngas conversion is a key area of research for sustainable aromatic hydrocarbon synthesis. The “one-step or direct method” using a multifunctional catalyst in a single reactor simultaneously contains the two different types of active species of the metal oxide catalyst and a molecular sieve catalyst. Among these, metal oxide catalysts can further be divided into the olefin (Fischer–Tropsch) synthesis and oxygenate (methanol) synthesis routes for two different reaction intermediate processes. The system represented by Fe- and Co-based metal oxides, such as FeMn,^89^ CoMn,^90^ FeCu,^62^ FeZn,^32^ FeMg,^91^ FeCa,^92^ FeZr,^93^ and FeCo^94^ catalysts, are based on the FTS route, leading to the olefinic/paraffinic intermediate products, while the systems represented by Cu-, Zn-, Zr-, or Cr-based metal oxides are categorized as the methanol synthesis route giving methanol/DME as the intermediate product.^26,67,95−97^ Therefore, the different intermediate products of the two routes lead to the different distributions of end-products for aromatic hydrocarbons.

#### 2.5.1 Brief Overview of the Methanol-Mediated Route

The methanol synthesis route,^98^ using different metal oxides, can efficiently convert CO and H_2_ to methanol. It is currently being adopted by different copper-based oxide catalyst research companies including UK ICI, Lurgi and Sud Chemie, Denmark Tropφe, UCI, and Mitsubishi. Meanwhile, the research units of China’s domestic copper-based oxides mainly include the Nanhua Group Research Institute, the Southwest Chemical Industry Research Institute, and the Qilu Petrochemical Company Research Institute. In addition, the precious metal Pt and Pd catalyst along with the effects of Al_2_O_3_, TiO_2_, ZrO_2_ Ga_2_O_3_, ZnO, Cr_2_O_3_, SiO_2_, and La_2_O_3_ supports also have a significant contribution on the conversion of syngas to methanol.^99,100^ The preparation process has been rigorously improved for enhancing the structural and electronic behaviors of the copper-based oxide, where the higher dispersion increases the catalyst life and further reduces the operating and production costs. Adopting composite catalyst using MTS catalysts is a way of converting syngas to the oxygenated compounds as the intermediate products that can be subsequently transformed to aromatics over HZSM-5.^101^ Mobil Corporation first reported in 1977 that oxygenates such as methanol could generate aromatic hydrocarbons from methanol on ZSM-5 molecular sieves; later, several new processes in terms of preparation methods, additive modification, and carriers were conducted on the MTS route in the multifunctional catalytic matrix.^26,66,67,78,97,100,102−104^ Usually, the use of a Cu-based metal oxide surrounding the industrial production zone as in the CuZnAl catalyst could be an efficient approach of high conversion rates and a wider hydrocarbon spectrum. However, the higher optimum temperature for aromatization compared to that of methanol synthesis on Cu-based catalysts restrict the catalytic performance of Cu-based catalysts for the sintering and thermal deactivation behavior.^98,104,105^ Therefore, the elevated temperature-resistance ability of zirconium can increase the dispersion of Cu species, delay sintering, and improve the activity and stability of the CuZnAl ternary catalytic system. Meanwhile, Zn and Zr-based oxides have also been commonly used in the methanol synthesis route, and their advantages such as stable chemical properties, oxygen vacancy anodes, strong ion exchange capacity, and surface acidity and alkalinity have attracted more attention for their integration with zeolites. ZrO_2_ itself has low methanol synthesis activity and therefore is usually categorized as a carrier or auxiliary agent, where its nanomaterials have the advantages of large specific surface area and small particle size. The ZnCr catalyst was found as an early high-pressure catalyst, where its core–shell ZnCr/Zn-ZSM-5 structure usually possessing a good stability and reactivity could improve the catalytic activity while effectively suppressing the formation of byproducts.^42,47,95,106^ Wang Ye et al. used Zn-ZrO_2_ and HZSM-5 molecular sieves to directly synthesize aromatics in one-step synthesis gas and found that the content of hydrocarbon-based aromatics reached 80% with a continuous stable life of more than 1000 h, although its conversion was not comparable to that of Cu for a maximum value of only 20%.^23^ Kaoru et al. and Yan et al. also implied Pd-based catalysts could be combined with different molecular sieves for the “one-step” reaction to produce aromatic rich oil products from biomass synthesis gas.^107,108^ Owing to the limitations in achieving the specific higher fractions of BTX, stable catalytic activity and temperature mismatching issues in Cu based catalysts or the lower single pass CO conversion rate by Zn-, In-, Zr-, Ce-, or Cr-based oxides by one-step methanol routes are the governing factors for the increasing concerns to shift toward the FTS route.^41,109−113^

#### 2.5.2 Fischer–Tropsch Synthesis As a Highly Versatile Process

FTS is a significant process that utilizes syngas as a feedstock to synthesize liquid hydrocarbons, employing specific catalysts and conditions, where numerous refinements and adjustments to the CO hydrogenation reaction technology have been made to the efficient techno-economic advancement, including catalyst development and reactor design.^114,115^ It stands out as a highly versatile process capable of converting syngas into a diverse range of hydrocarbons, basically referring to a catalytic chemical reaction in a process of gas to liquid (GTL) polymerization that turns the syngas as one of the carbon sources into the hydrocarbon chains by means of a metal catalyst as follows:^116,117^
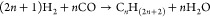


Here, catalysts crucial for producing valuable chemicals and fuels often contain metallic components such as single atoms, clusters, oxides, carbides, or alloy particles (for instance, cobalt-, rhodium-, gold–palladium-, metallic iron-, or iron carbide-based catalysts), resulting in various hydrocarbon chain growth phenomena. However, these metallic species face challenges related to poor catalytic stability and the predictions of the Anderson–Schulz–Flory (ASF) distribution model, limiting the selectivity for C_2_–C_4_ hydrocarbons, gasoline (C_5_–C_11_), jet fuel (C_8_–C_16_), and diesel (C_10_-C_20_) to 58%, 48%, 41%, and 40%, respectively.^118^ Therefore, the breakthrough lies in a novel catalytic principle that would sustain a stable efficiency and overcome the theoretical limits of the ASF distribution. Different mathematical models and theoretical studies of the product-distribution-based composite catalysts have been proposed by different researchers, adding to a new step for the ASF rule and giving a new horizon for the combination of different catalysts with different active sites.^65,119^ These endeavors aim to optimize the utilization of syngas, making it a more sustainable and versatile pathway to produce liquid fuels and chemicals from diverse feedstocks.

#### 2.5.3 Utilization of Carbon Dioxide as Feedstock for the RWGS-FTS Strategy

**Figure 4:**
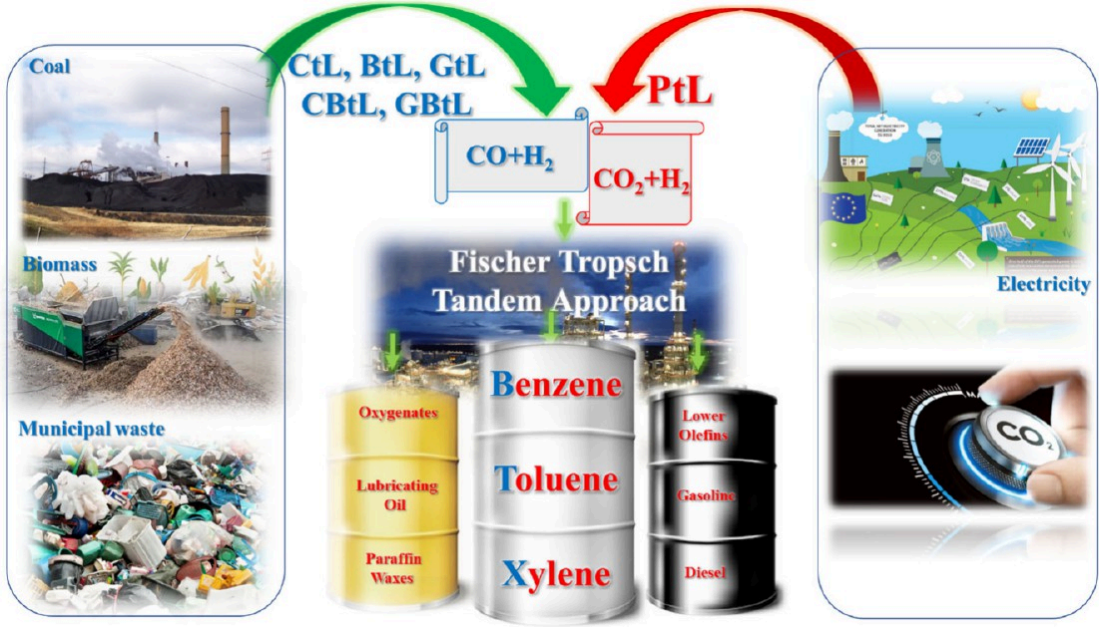
Potential pathways for the synthesis of aromatics through FTS. Reproduced with permission from ref (120). Copyright 2020 Elsevier.

CO_2_ hydrogenation technology has emerged as a highly effective strategy not only for reducing atmospheric CO_2_ levels but also for generating valuable chemicals and fuels, including gasoline, aromatics, alcohols, and olefins. In scenarios where CO_2_ is introduced as a feedstock or in a CO_2_-modified Fischer–Tropsch synthesis (CO_2_–FTS), tandem catalysis may include a separate set of reactions aimed at incorporating CO_2_ into the hydrocarbon products (Figure 4). This step contributes to the overall reduction of carbon emissions by utilizing CO_2_ as a resource. Therefore, the tandem catalytic approach excels in its ability to streamline multiple reaction steps, optimize selectivity, and enhance overall efficiency in the production of liquid fuels from syngas and CO_2_. However, rational catalyst design is still a hot topic in this era for increasing the selectivity of desired liquid (aromatic) hydrocarbons lasting for the industrial time scale. Co-based metal oxides exhibiting reduced water gas shift (WGS) activity have garnered considerable attention, particularly when combined with a zeolite for syngas to hydrocarbon technology. The notable interaction between cobalt and zeolite has proven instrumental in boosting catalyst performance, leading to enhanced C_6_^+^ hydrocarbon selectivity.^102,121^ Various kinds of alkali and alkaline promoters have been extensively reported in Co-based FTS processes, demonstrating the improved catalytic performance and product selectivity for long-chain liquid hydrocarbons.^122^ The utilization of Mo/Ni/K/ZSM-5 and Mo/Co/K/ZSM-5 catalytic systems, with potassium acting as the electron assistant and copper as an additional catalyst for the water–gas shift reaction, has been highlighted due to the substantial enhancement of conversion rates and the selective production of aromatic hydrocarbons.^65^ Nevertheless, cobalt-based oxides have found their niche in the low-temperature Fischer–Tropsch (LTFT) process, exhibiting a penchant for generating longer-chain linear alkanes.^123^ On the other hand, the distinctive shape-selection effect, acidic nature, and heightened aromatization ability of HZSM-5 molecular sieves shine at temperatures ranging from 300 to 400 °C.^4,107,124,125^ Therefore, integration with HZSM-5 for the high-temperature Fischer–Tropsch (HTFT) process (>300 °C) poses challenges due to increased methane production, detracting from the suitability for the STA process.^4,25,126^ Therefore, overcoming these hurdles and seeking improvements via composite materials for tandem catalysis with strategic designs represent critical avenues of research, underscoring the necessity of advancing the Fischer–Tropsch process to enhance selectivity for the desired hydrocarbons while mitigating environmental concerns associated with waste streams.

### 2.6 Fe as an Efficient and Old Compassionate Lad

Considerable research has been dedicated to developing efficient catalysts for the conversion of syngas involving multiple catalytic processes that selectively yield valuable products. The OX-ZEO tandem catalysis technology has evolved into a versatile hydrocarbon production platform, where the key function of this strategy would be the separation of two crucial steps, namely, the “activation of reactant molecules” and “intermediate carbon–carbon bond coupling” on two distinct active centers. The mechanism of a tandem catalyst to produce liquid fuels from syngas involves a sequential series of catalytic reactions, where the goal is to convert these gaseous feedstocks into liquid hydrocarbons. Initially, synthesis gas transforms into an active intermediate on the metal oxide catalyst surface (often based on iron or cobalt) to form linear hydrocarbons, primarily paraffins and olefins, as intermediates. Meanwhile, the generated FT synthesis intermediates (usually olefins) would adsorb and diffuse through the zeolite pore channel in a series of various cascading reactions (cracking, isomerization, polymerization, and cyclization) at the acidic sites, consequently increasing the CO conversion levels and breaking the ASF distribution law.^64,77,78^ Herein, the syngas composition (CO/H_2_ ratio) is crucial for subsequent reactions, where Fe-based catalysts are more favorable for syngas with a low H_2_/CO ratio as they exhibit high intrinsic selectivity for the water–gas shift (WGS) reaction. Therefore, the choice of catalyst is intricately tied to the reaction temperature, where Fe-based catalysts perform well as decent candidates to optimize the reaction at a higher temperature range (300–350 °C), consequently producing a higher fraction of lower olefinic products as intermediates for the upgrading phase (zeolite). Therefore, Fe-based catalysts have been widely explored in STA as compared to other FTS catalysts for their thermal stability and high-temperature operation, which favors the production of olefins with much lower selectivity to paraffins (Tables 2 and 3).^19,20,22−40,44,69,116,127^

**Table 2:** Comparative Reaction Results from Literature Data for CO Hydrogenation to Aromatics

	reaction conditions					hydrocarbon distribution (%)			
catalysts	*T* (°C)	H_2_/CO	*P* (MPa)	CO conv. (%)	CO_2_ sel. (%)	CH_4_	C_2_–C_4_	C_5_^+^ aliphatics	aromatics
Fe_3_O_4_@MnO_2_^128^	340	1.5	2	91.8	37.9	12.0	37.4	45.7	
Fe/CNF^129^	300	2.0	2	88.0	42.0	13.0	52.0	18.0	
Fe-MnK-AC^130^	320	2.0	1	85.0	48.0	22.7	39.4	29.7	
FeMnCu^131^	300	2.0	2	96.9	23.0	20.0	40.1	9.5	
MnCr/Z5^48^	430	4.0	1	13	45.0	6.7	19.5	2.9	70.9
Fe_3_O_4_@MnO_2_/Z5_hol_^127^	320	4.0	2	90.3	45	6.1	25.6	11.7	56.6
Fe_3_O_4_@MnO_2_/Z5_hol_^132^	320	2.0	1	85	41	5.1	25.4	10.5	59.0
FeMnKSiO_2_/Z5^133^	300	2.0	1	63.8	40.2	12.3	19.59	55.2	12.7
KFeMn/Z5^134^	320	2.5	1	81.1	40.9	12.3	29.7	17.3	40.7
Na-Zn-Fe_5_C_2_/Z5^32^	340	2.0	2	88.8	27.5	9.6	26.6	13.2	50.6
ZnCr/Z5^80^	350	4.0	1	18.3	49	1.5	16.9	12.6	69
KFeCo/Z5^135^	310	2.0	1	98.5	37.9	8.9	35.2	20.8	35.1
Na-FeMnCo/Z5^61^	350	4.0	1.5	98.1	21.5	18.9	11.6	14.4	55.1
Na-FeCa@AC/Z5^136^	350	4.0	2	96.86	21.2	8.57	11.81	19.79	60.1
Na-FeCuCo/Z5^62^	340	4.0	1.5	97.2	22.6	17.2	11.1	18.3	53.1
FeMn-Z5@Si-1^137^	320	1.0	1	60.0	42.7	22.5	32.5	11.4	33.6
FeMn-Z5@Si-2^137^	320	1.0	1	81.2	46.2	24.8	43.5	6	25.7
NaFeMn/Z5 (nano)^89^	350	4.0	2	95.3	27.4	18.2	17.5	10.5	53.8
NaFeMn-Si/Z5^138^	350	4.0	1	90.5	41.1	9.2	8.1	18.3	64.4
NaFeZnMg/Z5^139^	350	4.0	2	97	23.7	22.5	14.7	10.7	52.1
NaFeMg/Z5^140^	370	4.0	2	96.19	26.4	24.6	11.12	12.4	51.38
FeMn/Z5 (pm)^137^	320	1.0	1	49.4	39.8	19.9	41.8	9.6	28.7
FeMn/Z5 (layered)^137^	320	1.0	1	29.1	24.0	18.4	43.5	5.9	32.2
MoFe/Z5^141^	320	2.0	1	59.0	44.8	24.9	24.4	14.5	36.2
ZnCrO_*x*_-ZSM-5^142^	350	4.0	1	16.0	∼46	1.7	15.1	9.3	73.9
ZnAlO_*x*_/Z5^143^	360	5.0	2	56.5	46.2	8.2	24.9	4.5	62.4
Zn-ZrO_2_/Z5^23^	400	3.0	2	21.0	42.0	1	17	1	81.0
Fe-ZnCr_2_O_4_/Z5^30^	350	2.0	1	45	∼46.1	0.5	18.6	0.5	80.4

**Table 3:** Comparative Reaction Results from Literature Data for CO_2_ Hydrogenation to Aromatics^a^

	reaction conditions						hydrocarbon distribution (%)			
catalysts	H_2_/CO_2_	*T* (°C)	*P* (MPa)	GHSV (mL·g^–1^·h^–1^)	CO_2_ conv. (%)	CO sel. (%)	CH_4_	C_2_–C_4_	C_5_+ aliphatic	aromatics
ZnO-ZrO/Z5(300)^111^	3	340	4	2700	9.1	42.5	0.6			70.0
ZnCr_2_O_4_/Z5^144^	3	350	4	1200	23.1	27.8	0.5	12.3	1.8	85.3
ae-Zn-ZrO_2_/Z5^145^	3	340	4	2400	15.9	34	0.3	19.4	4.3	76.0
Fe_2_O_3_@KO_2_/ZSM-5^146^	3	375	3	5000	48.9	12.8	13.9	45.9	15.3	24.9
Na-Fe_3_O_4_/Z5(25)-Si-1 × 12%^33^	2	320	3	4000	28.5	15	6.2	32.7	18.1	43.0
Na-FeAlOx/Zn-Z5@SiO_2_^147^	3	370	3	4000	45.2	15.3	13.8	26.2	21.3	38.7
CuFeO_2_/0.15M-Z5(25)^148^	3	320	3	8100	52.8	7.3	4.1	14.1	13.4	69.7
Na/Fe-Z5^149^	3	300	1	4800	21.8	40.9	14.7	25.5	5.1	54.7
1Na-Fe/Z5^150^	3	340	3	4000	32.3	16.6	5.6	19.9	11.2	63.5
K-3Fe/Zn/Z5(21)^151^	3	320	3	7200	43.6	11.5	13.6	23.3	37.7	25.4
15Fe-10K/Al_2_O_3_&P-Z5^152^	1	400	3	6000	36.4	10.2	10.8	39.6	10.1	39.5
FeK1.5/HSG|Z5(50)^40^	3	340	2	26 000	35.0	39.0	3.5	4.4	24.0	68.0
ZnFeO_*x*_-4.25Na/Z5^83^	3	320	3	4000	36.2	6.9	11.1	16.5	15.6	60.0
Na-Fe@C/Z5^153^	3	320	3	6000	33.3	13.3	4.8	10.4	34.6	50.2
Na-Fe_3_O_4_/Z5^81^	2	320	3	4000	27.7	16.0	5.9	29.6	20.0	44.5
Cu-Fe_2_O_3_/Z5-pt^81^	3	320	3	1000	55.4	4.41	12.5	9.4	15.4	61.9
Cu-Fe_2_O_3_/Z5-dg^81^	3	320	3	1000	49.7	6.1	20.8	10.5	22.4	44.9
Cu-Fe_2_O_3_/Z5-hy^81^	3	320	3	1000	54.5	4.45	12.3	10.0	21.5	55.2
Na-FeAlO_*x*_/Zn-Z5(12.5)@SiO_2_^154^	3	370	3	4000	45.2	15.3	13.8	26.2	21.3	38.7
2.83Na-FeMn/Z5(105)^155^	3	320	3	4000	27.0	21.9	9.0	28.4	26.1	36.5
Fe&LZ5^156^	3	320	3	12 000	31.5	30.1	14.5	36.6	23.1	25.7
Na-FeMn/Z5@S1–S^155^	3	320	3	4000	20.7	34.4	11.3	37.2	28.0	23.5
ZnCr_2_O_4_/Z5@SiO_2_^157^	3	350	3		15.9	38.5	7.7	36.8	5.3	50.2
Cr_2_O_3_/C-500–500/Z5-S^158^	3	350	3	1200	25.4	70.6	2.1			80.1
ZrO_2_-Cr/Z5@SiO_2_^159^	3	360	4	1200	13.9	45.1	1.1	20.8	1.3	76.8
ZnZrO_*x*_/ZSM-5@RH^160^	3	340	3	15 000	12.2	∼80	1.1	∼20		74.7
ZnZrOx/Z5-0.73^161^	3	315	3	2040	17.5	23.8	1.2	29.8	7.7	60.3
Zn-UIO-66/Z5^162^	3	320	3	4800	20.4	33.8			1.1	85.3
ZnZr_7_O (500)-sheet-Z5^163^	3	320	3	2400	14.5	40.2				75.7
Fe-K/a-Al_2_O_3_&P/ZSM-5^152^	1	400	3	3000	36.4	10.2	9.7	35.6	9.0	35.5
NaFe/Z5(25)-Si-1 × 12%^164^	2	320	3	4000	28.5	15.0	6.2	32.7	18.1	43.0
2.3NaCuFe/HR-Z5-S^83^	3	320	3	1000	33.3	16.0	11.9	15.5	3.8	68.7
FeCu(100:3)/CLZ5–790^165^	3	340	3	4000	42.7	16.8	10.6	29.1	29.9	30.3
K/Fe-Cu-Al@Z5^166^	3	320	3	4000	∼44	∼6	∼8	∼32	∼30	30.6
3-NFC-5/Z5^34^	3	320	3	1500	42.1	10.7	9.0	11.9	22.3	56.8
NFC-DM/0.5M-Z5^167^	3	320	3	1500	46.2	6.5	8.1	10.4	25.4	56.1
Cr_2_O_3_/Z5^168^	3	350	3	1200	34.5					75.9
ZnZrO/Z5^110^	3	320	4	1200	14.0					73.0
ZnAlO_*x*_&Z5^38^	3	320	3	2000	9.1					73.9
Na-Fe_3_O_4_/Z5^169^	3	320	3	4000	33.6					40.9
ZnCrO_*x*_/ZnZ5^170^	3	320	5	2000	19.9					56.5
Cr_2_O_3_/Zn-Z5@SiO_2_^171^	3	350	3	1200	22.1					70.1
ZnCr_2_O_4_+Z5^172^	3	300	3	300	11.3					75.4
Na-ZnFeOx+Z5^29^	3	320	3	1000	41.2					75.6
FeMnOx+Z5^173^	3	320	3	1000	44.5					64.2
ZnCr_2_O_4_+Z5^174^	3	350	4	1200	23.4					66.0
CuFeO_2_/Z5^175^	3	320	3	8100	52.8					69.7
LaFe_2_O_3_+Z5^176^	3	350	3	1000	61.2					85.8

a C_2–4_ refers to lower olefins and C_2–4_ paraffin, C_5+_ includes C_5+_ aliphatic, pm refers to particle mixing, and Z5 refers to HZSM-5 zeolite.

#### 2.6.1 Active Fe Phase Synthesis, Reactions, And the Effect of Different Metal Additives

In the fascinating realm of syngas conversion to hydrocarbons using Fe-based catalysts, a symphony of active iron species takes center stage, each lending its unique flair to the catalytic performance. However, understanding the nature and role of these active iron species is crucial for designing and optimizing Fe-based catalysts for syngas conversion to hydrocarbons with the aim of improving catalytic efficiency, selectivity, and stability. Experimental techniques, such as in situ spectroscopy, microscopy, and theoretical modeling, are commonly employed to elucidate the active species and reaction mechanisms involved in these catalytic processes. Usually, iron oxide (FeO), metallic iron (Fe), and iron carbonyl complexes, being derived from different precursors or interchanging phases among each other during the pretreatment, reduction, or reaction process, are considered to play some catalytic role for LTFT or WGS/RWGS activities, which is crucial in the conversion of syngas to hydrocarbons. However, iron carbides (Fe_*x*_C) have been identified as the most active species in promoting specific reactions within FTS systems, influencing product selectivity and catalytic activity.

Carbides can be generally categorized as ionic, covalent, or interstitial carbides, where the size and electronegativity of transition metals enable to interstitially dissolve the C, O_2_, or N_2_ atoms into their crystal lattices.^177^ Fe, as a famous transition metal that has interstitially dissolved C in its crystal lattice, is referred to as Fe-carbide, where its unique chemical and physical properties and high melting points can often take encourage its application in various fields. Thus, the renewed attention toward Fe-based FTS catalysts has attracted many novel research approaches and efforts with advanced characterization techniques regarding the diverse role of carbon incorporation with Fe, which leads to the significant physical and chemical properties.^177,178^ The different structures of Fe_*x*_C with trigonal prismatic interstices and octahedral interstices can be classified according to the sites occupied by the carbon atoms. Here, (pseudo-)cementite (θ-Fe_3_C), Hägg carbide (χ-Fe_5_C_2_), and Fe_7_C_3_ are well-known carbides in FTS that usually have stable and clearly established structures. These iron carbides (Fe_*x*_C) have frequently been identified through various in situ characterizations and theoretical calculations as key contributors to CO activation and the phenomenon of chain growth.^32,179−182^ The unsaturation of the reaction product increases with the increase in Fe_*x*_C content during the reaction process, which is reflected in the increase in the selectivity of olefins intermediate products and the selectivity of aromatics in the final product.^183^ Over the past few decades, numerous fundamental studies have been conducted to address the challenges of sintering active Fe species and carbon deposition, which lead to increased deactivation rates. These efforts aim to facilitate the rational design of productive and stable Fe-based systems for Fischer–Tropsch (FT) synthesis.^184,185^ Among the various active species, Fe_5_C_2_ is notably recognized for its capability to adsorb and dissociate CO, as well as its effectiveness in promoting olefin formation, which is crucial for hydrocarbon chain growth. Additionally, magnetite (Fe_3_O_4_) is widely acknowledged for its significant role in WGS and RWGS activities, particularly in enhancing the CO_2_ fraction. While ε-iron carbide (ε-Fe_2_C) emerged as a promising FT catalyst, its instability under realistic reaction conditions (above 300 °C) posed a challenge.^186^

**Figure 5:**
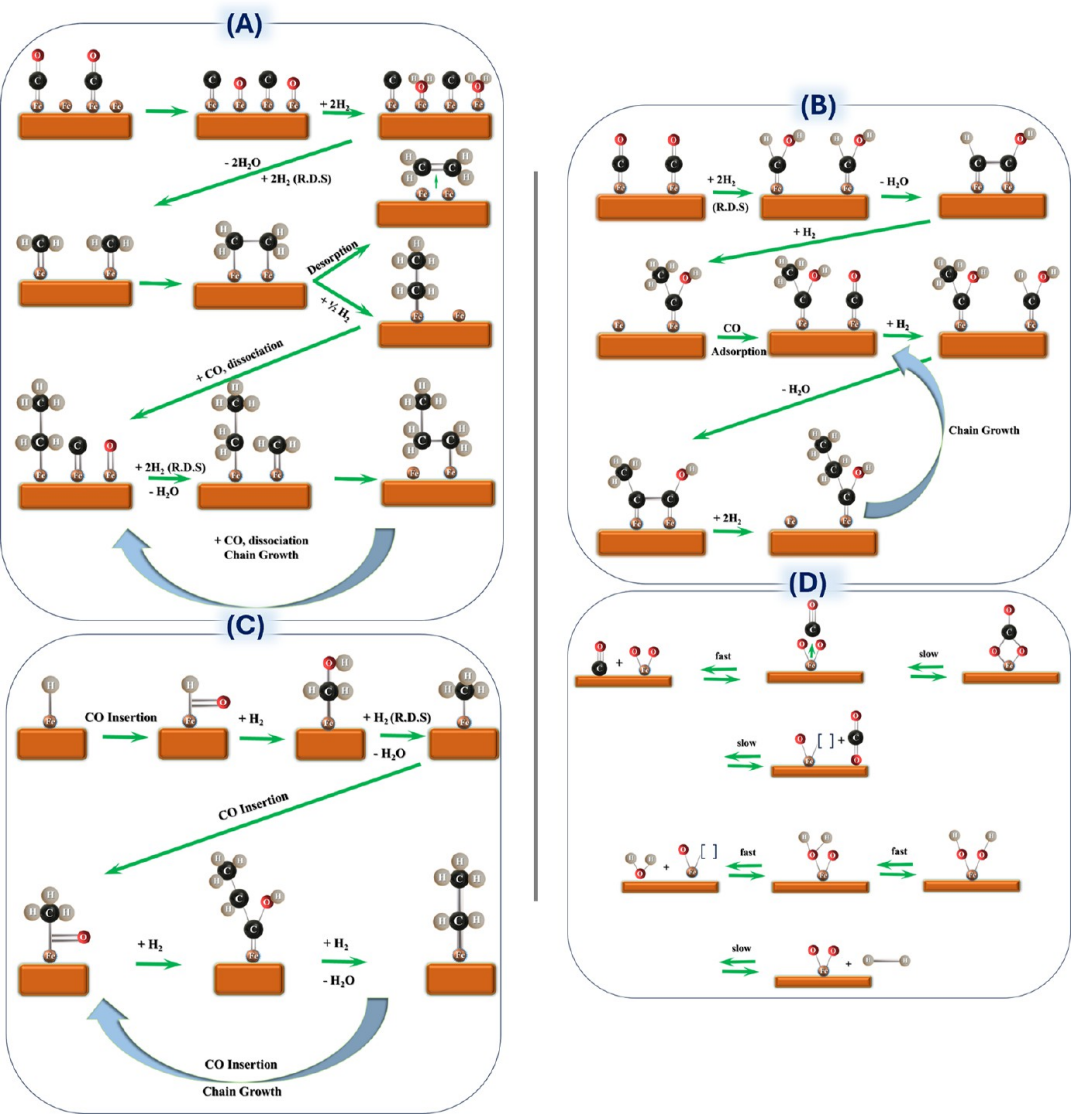
Overall mechanistic routes proposed on the Fe-based surfaces for (A) carbide mechanism, (B) enol mechanism, (C) CO-insertion mechanism, and (D) WGS reaction. Reproduced with permission from ref (181). Copyright 2008 Royal Society of Chemistry.

Notably, the difference between the FTS and the MTS routes resulting in different pathways also lies in the different intermediate products generated from different active centers of these systems.^26^ The interpretation of the FT synthesis complex reaction mechanism is fraught and filled with pitfalls, converting the two simple components of H_2_ and CO into a wide spectrum of olefin/paraffin hydrocarbons, along with a minor range of oxygenates. Since its discovery in 1923, the FTS mechanism has been elucidated through various proposed pathways, including the carbide mechanism, the CO-insertion mechanism, the enol mechanism, and CO_2_ FTS schemes (shown in Figure 5A–D). In the generation mechanism of lower olefins, the most important step is the formation of the first C–C bond, which is mainly categorized as the surface carbonization mechanism, enol mechanism, and CO insertion mechanism.^187^ It is believed in the surface carbide mechanism that CO will be adsorbed and dissociated on the FTS catalyst surface with the formation of metal carbide, followed by chain growth with CH_2_ insertion. Here, the free oxygen can be combined with hydrogen to form water, and carbon atoms will form carbides with the reduced metal. The active species (such as Fe_5_C_2_) then undergo hydropolymerization to form C–C linkages, where the mobile CH_2_^–^ species freely move over the catalyst surface.^4,114,181,188^ The oxygenate (enol) mechanism, on the other hand, involves CO chemisorption and chain growth through nondissociative adsorption of CO, where the first C–C linkage is achieved through the enol structure (HCOH), with the condensation and water elimination steps using adjacent groups. It is different from the surface carbide mechanism, in which CO is only physically adsorbed on the metal surface and then combined with dissociated H_2_ to form the HCOH structure, followed by the dehydration or self-reaction of two hydroxy carbenes to form a C–C bond.^189^ The proposed mechanism of CO as the only carbon source has demonstrated that CO can be directly hydrogenated to form methanol, while CO_2_ cannot be directly hydrogenated on the metal catalyst surface.^190^ When some CO_2_ is mixed into the syngas, the reaction rate of methanol synthesis increases, which cannot be explained by the single-carbon-source mechanism of CO. Some studies believe that CO_2_ is the only carbon source and that only it can be hydrogenated in the methanol synthesis process, while CO may not undergo the direct hydrogenation step.^191^

**Figure 6:**
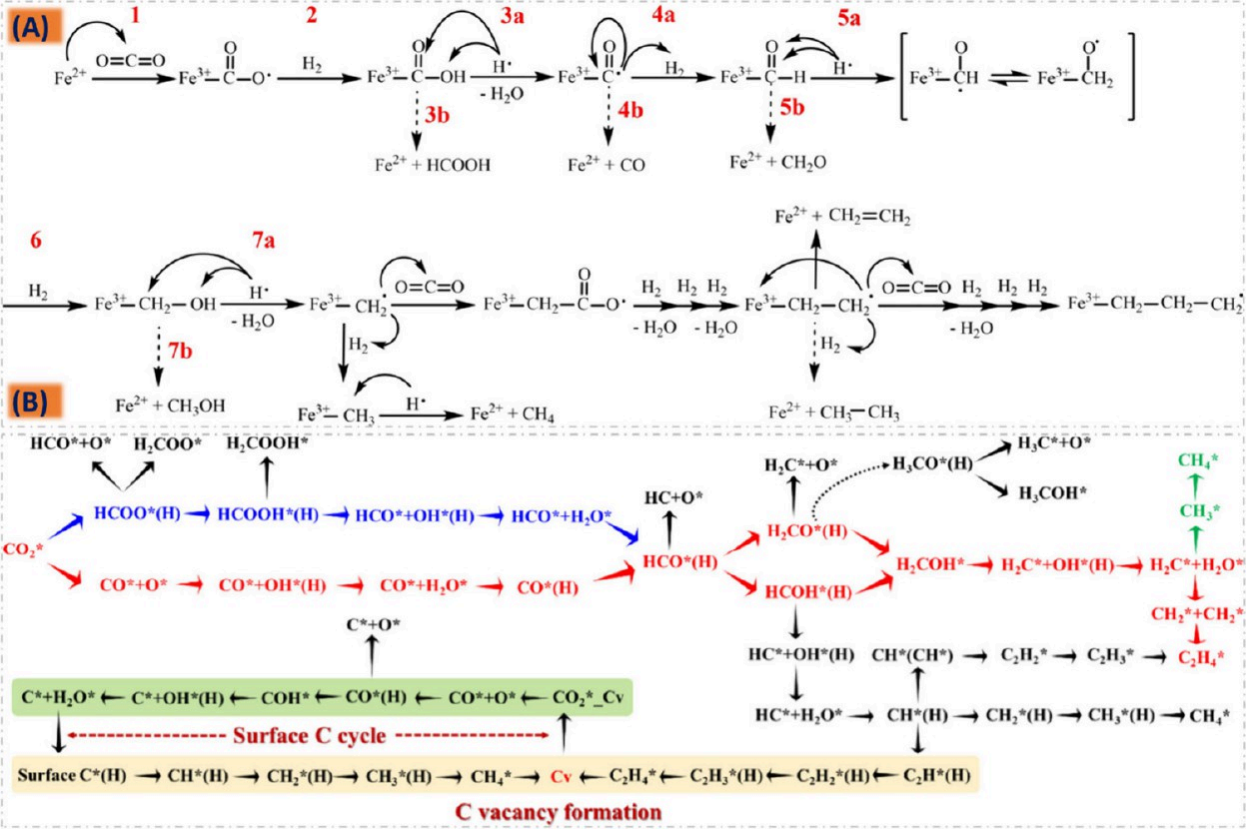
CO_2_ hydrogenation schemes producing different intermediate species on an Fe-based catalyst. (A) Reproduced with permission from ref (192). Copyright 2019 Willey and Sons. (B) Reproduced with permission from ref (193). Copyright 2023 American Chemical Society.

While at present the double-carbon-source mechanism has also been explained for the methanol synthesis reaction, demonstrating that both CO and CO_2_ can undergo hydrogenation to generate methanol, the intermediate products after hydrogenation are different. Recently, Xu et al.^194^ presented a comprehensive reaction mechanism for a direct pathway from CO_2_ to methanol via HCOO* intermediates facilitated by Cu–ZnO interfaces in Cs-CuFeZn catalysts. It was demonstrated that the presence of the Cs promoter hampers the methanol synthesis rate, and at elevated temperatures (>300 °C) the thermodynamics limits methanol production, favoring CO formation. Conversely, higher alcohol synthesis becomes favorable at elevated temperatures due to Cu–ZnO’s ability to catalyze the reverse water gas shift (RWGS) reaction, generating CO, and copper–iron carbide facilitating C(H)O* insertion to form higher alcohols. Furthermore, the Cs promoter enhances the CO insertion reaction during the CO_2_-to-HA process by regulating the hydrogenation ability of the catalyst, while methanol is generated over a methanol synthesis catalyst, which is further converted into light olefins or dehydrated to DME first and then converted to light olefins.^66,195,196^ Meanwhile, different perceptions have also been presented in this scenario, where a mechanism involving carbonyl (CO) insertion into a metal–alkyl bond, which is different from the previous two mechanisms, is also distinctive. Here, only H is adsorbed on the catalyst surface, and then CO is inserted into a metal–hydrogen bond to form a carbonyl (CHO) structure, while the CHO structure is easily hydrogenated to form a hydroxyl (−OH)) structure, and the reinsertion of CO after −OH hydro-dehydration forms a C–C linkage^197^ (Figure 6A and B). In a recent study by Guo et al, it was demonstrated that, aside from the carbide mechanism, Co_3_Fe_7_ sites could generate ample oxygen-containing intermediates (CO*, HCOO*, CO_3_^2^*, and HCO_3_*) that facilitate subsequent chain propagation via the oxygenate mechanism.^17^ A record-breaking C_5_^+^ yield of 26.7% and C_5_^+^ selectivity of 57.8% at a CO_2_ conversion rate of 50.2% were claimed for the novel iron-based catalyst modified with cobalt (KZFe-5.0Co) in response to the cobalt playing a crucial role in enhancing the reduction processes and strengthening the interaction between CO_2_ molecules and iron species. Furthermore, it was interpreted that the proposed reaction species could lead to reinforced cascade reactions between the reverse water gas shift (RWGS) reaction and chain propagation.

Meanwhile, let us not forget the unsung heroes of surface defects and active sites, whose subtle contributions, like secret whispers in the wind, amplify catalytic prowess by encouraging the adsorption and activation of reactant molecules. Recently, surface defects and active sites on the catalyst surface have also contributed to its catalytic activity, where these sites may involve coordinatively unsaturated iron atoms, steps, or kinks on the catalyst surface at which adsorption and activation of reactant molecules occur (this will be discussed in the following section). This captivating tale underscores the paramount importance of unraveling the dynamic interplay among these diverse iron species, guiding the artisanal craft of tailoring Fe-based catalysts toward a crescendo of enhanced efficiency, selectivity, and longevity in the grand theater of syngas conversion.

#### 2.6.2 Electronic and Structural Modifiers

Electron additives typically denote metallic elements possessing electron-donating characteristics. Alkali/alkaline promoters, on the other hand, are known for their ability to donate electrons to the vacant d-orbital of iron (Fe), thereby reducing the work function at the catalytic surface composed of Fe^0^/FeO_*x*_/Fe_*x*_C. This reduction enhances the stability of the carbide phase,^61,153,198,199^ thus restraining excessive H_2_ adsorption and promoting CO chemisorption and dissociation, which facilitate carbonization processes. The seminal work by Chang et al.,^24^ dating back to the 1970s, initially deviated from the ASF distribution law and has since undergone rigorous modifications with various effective techniques to enhance carbon monoxide hydrogenation. Although iron’s abundance makes it an attractive catalyst source, challenges persist, including significant accumulation of inactive carbonaceous deposits and increased selectivity for CO_2_ as a waste byproduct.^4,181^ Thus, there is a pressing need for novel, efficient metal catalysts capable of improving catalytic performance for carbon monoxide hydrogenation while mitigating anthropogenic impacts, particularly in reducing CO_2_ emissions. Several studies have demonstrated the effectiveness of incorporating diverse active transition metal promoters, not only in accelerating the reaction rate of iron oxide but also in significantly enhancing the selectivity of olefinic intermediates by suppressing excessive surface hydrogenation at higher CO conversion rates (85–95%).^32,199−202^ In various studies, Liu et al. have demonstrated that employing different metal components (such as Na, Cu, Mn, Zn, Zr, Ca, Mg, and Co) as common electronic promoters leads not only to an enhancement in the reaction rate but also to a significant improvement in the selectivity of olefinic intermediates by mitigating excessive surface hydrogenation at higher CO conversion levels.^26,27,35,36,81,89,136,138,200,203^ The reciprocity of the promoters elucidated that these additional components could take part in tuning the reducibility of iron oxides and modulating the carburization behaviors. Specially, Mn has been found to be a potential candidate that can have sterling catalytic performance with the effect of the silica–alumina ratio, ion exchange modification, and particle size on the reaction of ZSM-5 zeolite.^32,138^ Li et al. and Yang et al. modified the FeMn catalyst with Na and K promoters, where the added alkali metal species promoted the formation active Fe_*x*_C phase during the reaction and increased in the long chain molecules selectivity of C_5_^+^.^89,138,201,204^ Although it is established that adjusting the alkali content appropriately can enhance the carbonization of Fe, excessive addition of elements, such as Na and Mn, may result in the occlusion of surface-active sites. This can reduce selectivity toward aromatics, increase grain size, and hinder H_2_ adsorption,^36,61,89^ thus accelerating the carbon deposition and deactivation of Fe-based catalysts.^136,139,205^

Cu has been identified to play a role in facilitating the coupling of carbon bonds and promoting chain growth phenomena to enhance the structural and reduction capabilities of Fe-based catalysts during the selective hydrogenation of carbon monoxide (CO).^81,115,206^ Incorporating a specific quantity of copper oxide can effectively modify the surface and structural characteristics of Fe-based catalysts, leading to enhanced CO adsorption and stabilization of intermediates.^34,62,131,207^ It can improve the dispersion and promote reduction; however, careful control is needed to navigate the high temperature resistance.^208^ Fe-MnO/ZSM-5, Fe-Cu-MnO/ZSM-5, and Cu-Fe_2_O_3_/ZSM-5 catalysts in different studies have been found to produce aromatics in a one-step synthesis process, illustrating that the introduction of smaller amounts of Cu and Mn is beneficial to the formation of olefins during the FTS reaction and improves the selectivity of aromatics.^36,81,203^ Meanwile, FeCuSi catalysts modified with transition metals such as Zr, Mn, Mo, and Cr have demonstrated that Zr could improve the adsorption performance of CO and the selectivity of light olefins.^209^ Similarly, Cu-based Fe catalysts designed by Yang et al.^26^ and Guan et al.^210^ indicated that the addition of Cu could enhance the metal active sites of Fe species, which could in turn accelerate the formation of olefinic intermediates. Zn has also been found to increase the product selectivity.^211,212^ For example, Zhao et al.^32^ and Saif et el.^139^ applied FeZnNa/Z5 to the synthesis of aromatic hydrocarbons in one-step process and demonstrated that the combination of FeZnNa and Z5 has a great influence at the FTS product distribution, giving a selectivity of 53% for the formation of aromatic hydrocarbons with 85–95% CO conversion. Thus, various transition metals have played a crucial role in enhancing the desired product selectivity. However, the synergy between these metal oxides varies depends on factors such as the loading amount, the preparation method, the operating conditions, and the utilization of surface/electronic modifiers. Unlocking the optimal combination and interplay of these elements is key to maximizing the catalytic efficiency and achieving superior outcomes.

Employing a diverse array of high-porosity structural modifiers, including γ-Al_2_O_3_, SiO_2_, and TiO_2_, is commonly acknowledged for its ability to mitigate the sintering of metal active components. This practice enhances the morphological characteristics of iron nanoparticles, resulting in improved catalytic performance and longevity,^185,213,214^ since in the case of an active species containing the catalytic activity for a specific reaction the metal active component can be supported on the carrier to obtain a supported catalyst with two active sites. Structural additives such as Al_2_O_3_, ZnO, MgO, ZrO_2_, and SiO_2_ are active components and often interact with Fe, for their addition is more conducive to the adsorption of CO and can also enhance the carburization effect to improve C_5_^+^ selectivity.^206,215,216^ The robust metal–support interaction (MSI) can result in the formation of hard-to-reduce species and the aggregation of individual active metals in harsh reaction conditions, leading to facile deactivation.^4,217^ Meanwhiole, carbon nanofibers, carbon spheres, activated carbon, and carbon nanotubes present themselves as promising solutions, providing high specific surface areas, thermal and chemical inertness, efficient surface chemistry, and excellent recycling properties. Consequently, they offer effective means of dispersing active iron species, thus mitigating deactivation and enhancing catalytic performance.^180,218,219^ The discrete metal nanocrystals dispersed on carbon support particles offer significantly increased surface area and appropriate texture/pore structure for effective utilization of the active metal compounds vigorously synthesized by various techniques, including the solid-state reaction, sol–gel, or wet-chemical synthetic process.^168,213,220,221^

#### 2.6.3 Fabrication Strategies

**Figure 7:**
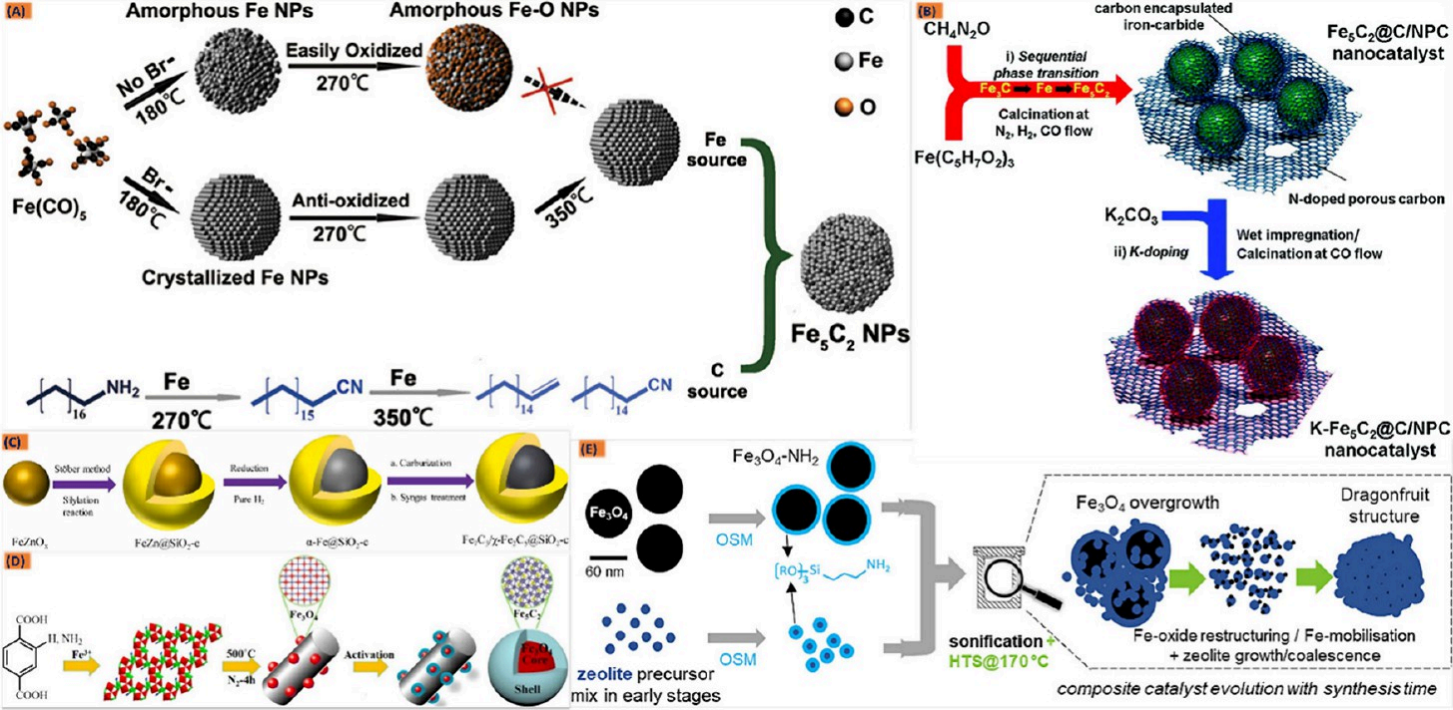
Schematic illustration of different synthesis methods for Fe-carbide. (A) Fe_5_C_2_ nanoparticles via a facile bromide-induced synthesis method. Reproduced with permission from ref (221). Copyright 2012 American Chemical Society. (B) K-doped Fe_5_C_2_@C/NPC nano catalyst through a sequential thermal treatment and the incipient wetness method. Reproduced with permission from ref (225). Copyright 2021 Royal Society of Chemistry. (C) Core–shell structured hydrophobic Fe_7_C_3_/χ-Fe_5_C_2_ @SiO_2_-c nanosphere by a classical silylation reaction. Reproduced with permission from ref (226). Copyright 2023 Elsevier. (D) Fe_3_O_4_@Fe_5_C_2_ core–shell nanocrystals by pyrolysis of a MOF. Reproduced with permission from ref (227). Copyright 2016 American Chemical Society. (E) Oxide and carbide restructuring during Fe/zeolite genesis. Reproduced with permission from ref (29). Copyright 2023 Elsevier.

Nonetheless, achieving optimal structural and compositional properties of the generated Fe_*x*_C species via a specific technique involving multistep fabrication strategies necessitates competing with the dynamic phase evolution processes prone to oxidation during the reaction^88,180,181,218,222^ (Figure 7). Yang et al.,^221^ in an earlier report, unveiled a straightforward wet-chemical method for synthesizing Hägg iron carbide (Fe_5_C_2_) nanoparticles, with bromide serving as the crucial agent for transforming Fe(CO)_5_ into Fe_5_C_2_ (Figure 7A). Similarly, Xu et al.^223^ proposed a catalyst primarily composed of ε-iron carbide by introducing an innovative approach using rapidly quenched skeletal iron, which undergoes swift carbonization during LTFT at 423–473 K. These newly synthesized Fe_5_C_2_ nanoparticles, when employed in Fischer–Tropsch synthesis (FTS), demonstrated inherent catalytic activity and outperformed traditional the iron/cobalt or costly noble ruthenium catalyst, exhibiting excellent selectivity for liquid fuels and impressive stability. On the other hand, different recent approaches have gained momentum in this area, with Chen et al.^224^ introducing a cost-effective and facile method involving a carbon-supported Fe catalyst (K-Fe/NC) with customizable FTS selectivity. The catalyst with the core–shell structure of K-Fe/NC derived from the pyrolysis of Prussian Blue demonstrated remarkable catalytic activity predominantly within the C_2_–C_13_ range. Temperature-programmed surface reactions along with theoretical calculations that shed light on the CO dissociation and association mechanisms suggested a synergistic effect between Fe_5_C_2_/ε-Fe_2_C and Fe_3_O_4_ active sites. Lee et al.^225^ presented both theoretical and experimental findings unveiling the potent influence of K promotion comprising carbon-encapsulated iron-carbide nanoparticles supported on nitrogen-doped porous carbon (Fe_5_C_2_@C/NPC) (Figure 7B). The synthesized nanocrystals dazzle with their exceptional performance, boasting a staggering CO conversion rate reaching a remarkable 96.7% over a time-on-stream period of 78 h. Notably, their selectivity for C_5_–C_13_ linear α-olefins peaks at an impressive 16.5%, accompanied by a productivity rate of 5.9 CH_2_ μmol·g_cat_^–1^·s^–1^. The computational model mirrors these experimental triumphs, corroborating the affirmative impact of potassium, even in minute quantities (approximately 1 wt %, K/Fe = 0.05), on the FTS reaction and heralding a promising frontier in catalytic design. Liu et al.^226^ unveiled a fascinating discovery of the dynamic tailoring of the metastable Fe_7_C_3_ phase through subtle adjustments in the shell thickness of hydrophobic SiO_2_ surrounding a core–shell nanostructured FeZn@SiO_2_-c nanocatalyst (Figure 7C). While χ-Fe_5_C_2_ reigns supreme as the dominant iron carbide phase in FeZnO_*x*_ and FeZn@SiO_2_ nanocatalysts, a plethora of Fe_7_C_3_ phases emerge within the realm of hydrophobic FeZn@SiO_2_-c nanospheres. A captivating transformation unfolded as FeZn@4.1-SiO_2_-c exhibited a drastic >70% decrease in CO_2_ selectivity compared to FeZnO_*x*_ yet simultaneously achieved a remarkable 1.7-fold increase in olefin selectivity during syngas conversion. The hydrophobic interface orchestrated this symphony by quenching the WGS reaction, thereby facilitating the swift diffusion of water. The resulting H_2_-lean and CO-rich microenvironment nurtures the formation of Fe_7_C_3_ and fosters the production of olefins. Meanwhile, in a groundbreaking study by Bing An et al.,^227^ an exceptionally potent catalyst for Fischer–Tropsch Synthesis (FTS) was unveiled, achieved through the ingenious pyrolysis of iron-infused metal–organic frameworks (MOFs). The process yielded nanoparticles boasting a distinctive iron oxide@iron carbide core–shell architecture meticulously dispersed on carbon supports, (Figure 7D). The resultant structure exhibited remarkable prowess in FT synthesis, outperforming most known catalysts by a significant margin, with Fe-time yields reaching an impressive value of 720 μmol_CO_·g_Fe_^–1^·s^–1^. The key innovation lay in overcoming the pervasive challenge of strong metal–support interactions encountered during conventional calcination processes, which often compromise catalytic activity due to difficulties in reducing metal oxides. By leveraging the unique properties of MOFs, the researchers successfully mitigated this issue, achieving full coverage of carbonates on particle surfaces through decarboxylation of the MOFs. This process not only stabilized the particles but also left behind an active surface rich in dangling bonds, thereby enhancing catalytic turnover.

Similarly, in a parallel breakthrough, Lyu et al.^186^ introduced a promising strategy for crafting a highly active and durable Fe-based FT catalyst. This involved the ingenious stabilization of pure-phase ε-Fe_2_C nanocrystals ensconced within the graphene layers. The formation of ε-Fe_2_C was facilitated by carburization of the α-Fe precursor, a process elucidated by density functional theory (DFT) calculations, which highlighted the crucial role of interfacial interactions within the ε-Fe_2_C@graphene structure. The resulting ε-Fe_2_C@graphene catalyst exhibited an exceptional iron-time yield of 1258 μmol_CO_·g_Fe_^–1^·s^–1^ under realistic FT conditions, eclipsing traditional carbon-supported Fe catalysts by an order of magnitude. Remarkably, this superior performance was sustained for a minimum of 400 h under elevated temperatures, underscoring the catalyst’s robustness and long-term stability. These groundbreaking advancements promise to revolutionize the landscape of catalysis, offering unprecedented efficiency and durability in FTS applications. However, there have always been different surface reactions occurring in different environments of Fe-based FTS process that could lead to different active or inactive reaction species.^228,229^ In a pioneering study by Paalanen et al.,^230^ a cutting-edge in situ XRD and Raman spectroscopy setup shed light on the circuitous interplay between the Boudouard reaction and FTS on sodium–sulfur-promoted iron-based catalysts, particularly at temperatures surpassing 300 °C. This innovative approach enabled real-time monitoring of the evolution of various iron phases (Fe_*x*_O_*y*_/α-Fe/Fe_*x*_C) and carbon species under conditions mimicking FTS at 340 °C and 10 bar, with a focus on the CO-carburized Na–S-promoted and unpromoted Fe(-Na-S)/α-Al_2_O_3_ catalysts. The study uncovered fascinating insights, revealing the transformation of amorphous carbon featuring both C(sp^3^) and C(sp^2^) configurations into carbon nanofiber-like structures during FTS. Moreover, the introduction of Na–S promotion and CO carburization at temperatures ≥340 °C led to a notable increase in the presence of 6-fold cyclic C(sp^2^) species. Initial carbon deposits within the catalysts served to mitigate the rapid escalation of Raman band intensities, while Na–S promotion significantly enhanced the subsequent intensity growth. Crucially, the evolution of carbon species remained unaffected by the presence of specific Fe carbides or their transitional states. Furthermore, Na–S promotion facilitated the reduction of Fe_3_O_4_ by (H_2_:)CO, consequently promoting the formation of carbon-depositing Fe carbides. These findings offer valuable insights into the complex dynamics governing carbon deposition and catalyst performance, paving the way for enhanced understanding and optimization of Na–S-promoted iron-based Fischer–Tropsch catalysts.

Weifeng Tu et al.^231^ investigated the utilization of Na–Fe_5_C_2_–ZnO catalysts for the conversion of CO_2_ into linear α-olefins and revealed a dynamic interplay between the RWGS and CO hydrogenation processes, with each process exerting varying degrees of influence. Where the RWGS reaction operated at a chemical equilibrium, the conversion of CO_2_ was primarily governed by thermodynamic principles. Meanwhile, the addition of Na to the catalyst system facilitated the modulation of the reaction pathways, effectively reducing the formation rates of paraffins during CO hydrogenation and enhanced the interaction between olefins and hydrogen. Additionally, Na altered the surface properties of the catalyst by promoting the interaction between CO* and H*, thereby facilitating the formation of C–C bonds and revolutionizing the catalytic landscape, which led to improved turnover rates of olefins. Similarly, Wang by et al.^29^ discovered a potential route of developing sinter-resistant oxide-zeolite composite materials with uniformly dispersed oxide nanoparticles tightly integrated within a zeolite matrix, as proposed Figure 7E. The study offered insights into the mechanism of metastable oxide–zeolite reorganization during cohydrothermal synthesis and organosilane modification, featuring intimately dispersed Fe_3_O_4_ nanoparticles 1–4 nm in size within a slightly mesoporous Al-ZSM-5 matrix throughout. They claimed the catalyst exhibited remarkable performance, yielding a high aromatic productivity of 214 mmol_aromatics_·h^–1^·g_Fe_^–1^, with an impressive 62% selectivity toward aromatics in hydrocarbons, while demonstrating excellent resistance to sintering and maintaining stable aromatics production for over 500 h. We have also introduced a groundbreaking method aimed at developing a highly efficient and durable iron-based integrated catalyst, denoted as Na-FeCa@AC/HZSM-5, for the STA process. Departing from conventional techniques utilizing Fe/C catalysts, our approach leverages spatial confinement strategies to disperse Fe_3_O_4_ nanocrystals within a robust graphite framework, effectively curtailing the issues of sintering and agglomeration encountered with Fe nanoparticles.^136^ By precisely controlling the addition of promoters (AC, Ca), adjusting calcination parameters, and managing surface carbon reactions during the sol-precipitation phase, we attained elevated levels of CO chemisorption on the Fe surface, consequently leading to the formation of carbide nanoparticles with exceptional purity (44% θ-Fe_3_C + 54% χ-Fe_5_C_2_). Further illustrations revealed that carburizing the generated Fe_3_O_4_ nanocrystals in the syngas pretreatment environment resulted in the formation of carbon patches that encapsulated the active carbide phase, thereby stabilizing it throughout the reaction. Notably, the composition of resultant multilayer carbide films facilitating the rapid CO dissociation and achieving 97% CO conversion with 60% aromatics selectivity during the reaction dynamically shifted to 70.7% χ-Fe_5_C_2_ ⇌ 27.3% θ-Fe_3_C, possibly in response to the decomposition of olefinic intermediates. Here, the convergence of multidisciplinary research efforts into the engineering of graphite architectures or design of phase-stable Fe carbides, heralding a paradigm shift toward enhancing carbon efficiency in Fe-based nanocatalysts, underscores the remarkable electromechanical properties that attracted the encapsulation of active Fe nanocomposites.^87,232^ In essence, the fusion of Fe carbide with HZSM-5 is a strategic maneuver, elevating the selectivity toward aromatic hydrocarbons while fortifying the catalytic stability and ushering in the era of resource efficiency for FTS-mediated pathways of aromatic synthesis. Despite hurdles like catalyst deactivation, this integrated catalyst system shines brightly on the horizon of industrial applications, presenting a clear path toward the sustainable production of coveted aromatic compounds from syngas.

#### 2.6.4 Defect Engineering and Boosting Surface Oxygen Vacancies in Fe–O_*x*_

**Figure 8:**
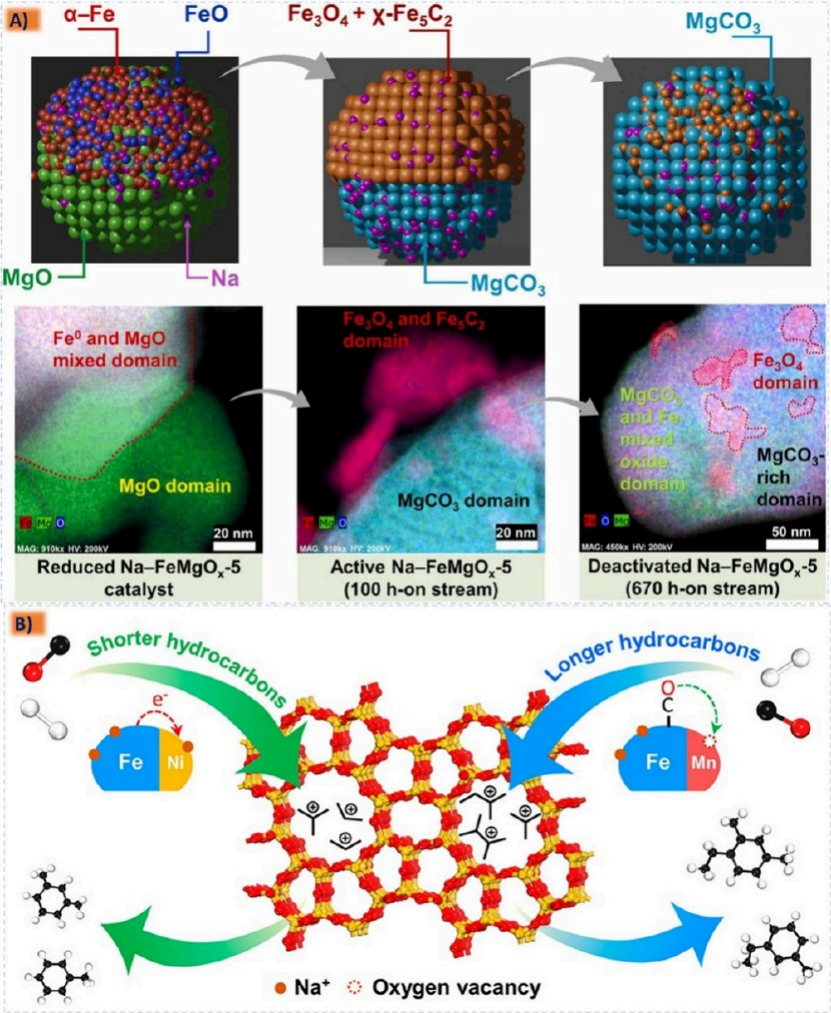
(A) MgO promoter facilitating the oxygen vacancies to accelerate the reduction of Fe oxides and the formation of Fe-based phases for CO_2_ hydrogenation to long-chain hydrocarbons. Reproduced with permission from ref (240). Copyright 2023 Elsevier. (B) Promoting Fe reduction using the presence of oxygen vacancies in NiO on the sodium-mediated bimetallic Fe–Ni catalyst for selective production of light aromatics over HZSM-5 zeolite. Reproduced with permission from ref (31). Copyright 2021 American Chemical Society.

Oxygen vacancies (O_v_), being the predominant point defects in oxide materials, are often present at low concentrations, making them difficult to detect; however, their concentration can be increased during manufacturing processes, such as by doping films or incorporating them into the crystal lattice to balance the charge introduced by dopants.^233^ Additionally, postproduction methods like electric field cycling, electron bombardment, UV irradiation, or rare-gas ion sputtering can generate these defects, reducing the surface oxygen content. O_v_ possesses unique physiochemical and electronic properties that make it important in various scientific and technological fields, such as facilitating electron–hole separation, surface charge transfer, and serving as sites for molecular oxygen adsorption and activation, forming superoxide anion radicals (O_2_^–^).^234^ The resultant core holes are typically filled by neighboring oxygen atoms through interatomic Auger processes, leading to charge redistribution at interfaces. Consequently, inducing surface O_v_, by manipulating the geometric and electronic structure of metal oxides, significantly enhances the intrinsic activity of heterogeneous catalysis.^46,61,160,235^ Notably, the sites more conducive to the adsorption of reactive species, influenced by the manipulated electron density and generated vacancies, have been shown to catalyze the chemisorption of CO/CO_2_ molecules during catalytic CO_2_ reduction.^236^ Various methods are employed to regulate the abundance of oxygen vacancies, including chemical reductant treatment, hydrogen reduction treatment, electrochemical reduction, active metal reduction, metal doping effects, and the creation of multiple phase interfaces.^13,46^ Investigations into the impact of these oxygen vacancies have primarily focused on methanol-mediated pathways employing Cu-, Mn-, Zn-, Zr-, or Pt-based catalysts.^15,143,237^ However, the trend is now shifting in recent studies exploring the influence of these oxygen vacancies in CO_2_ hydrogenation or Fischer–Tropsch synthesis routes, particularly when employing Fe-based catalysts.^81,229,238,239^ Recently, Ahmed et al.^240^ investigated the enhanced catalytic activity using the Na-FeMgO_*x*_ catalyst while discussing the transformation of MgO to MgCO_3_ during CO_2_ hydrogenation, the promotional effect of MgO, and deactivation mechanisms (Figure 8A). The model catalytic system Na-FeMgO_*x*–5_ exhibited a promising C_5_^+^ yield and CO_2_ conversion at the onset of the reaction under optimal conditions, where MgO enhanced the reduction of Fe oxides to metallic α-Fe and promoted the formation of oxygen vacancies. The bimetallic Na-FeMgO_*x*–5_ catalyst facilitates the formation of active phases during the reaction. Additionally, MgO enhances the adsorption of CO_2_ and H_2_, promoting long-chain hydrocarbon formation. However, the transformation of MgO into inactive MgCO_3_ would lead to a decline in C_5_^+^ selectivity due to the reoxidation of active phases, emphasizing the importance of selecting a metal oxide promoter that maintains its electronic states during CO_2_ conversion for developing a stable Fe-based catalyst. Meanwhile, Wang et al.^31^ demonstrated that Na modifying a FeNiO_*x*_ catalyst in a specific ration (FeMnO_*x*_(5:1)-0.40Na) can regulate superior catalytic activity for the production longer olefin-rich hydrocarbons by faciliating the reduction and carburization of FeO_*x*_ via the O spillover, thus leading to oxygen vacancy-assisted CO dissociation and conversion efficiency and finally resulting in highly branched monocyclic aromatics (Figure 8B). However, it was illustrated that faster HZSM-5 deactivation may occur due to increased coking activity from the isomerization-hydrocracking of long-chain hydrocarbons, thus requiring a careful regulation of both (Ni and Na) elements in the composite catalyst configuration.

**Figure 9:**
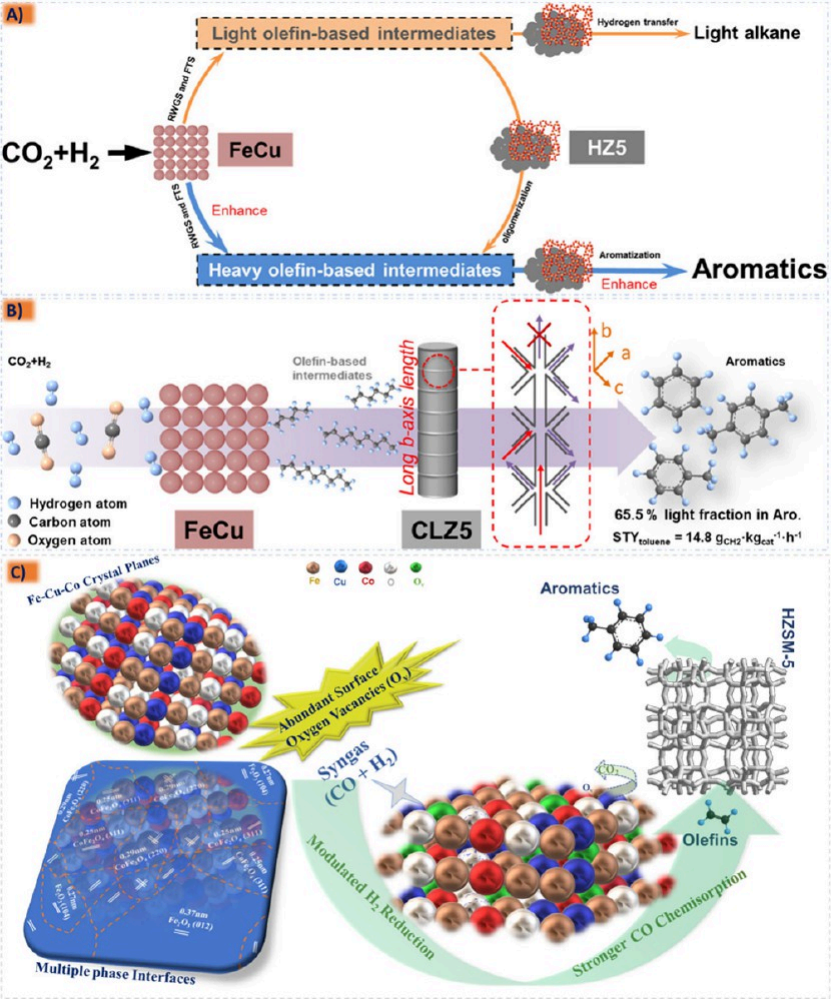
Effect of Cu promoter accelerating the formation of surface oxygen vacancies on the (Fe) metal active phase for accelerating aromatic synthesis. (A, B) Reproduced with permission from ref (241). Copyright 2023 American Chemical Society. (C) Reproduced with permission from ref (62). Copyright 2021 Royal Chemical Society.

Cu has usually been defined as an electron density modifier for Fe-based catalysts.^20^ Therefore, a bifunctional catalyst composed of a Cu-modified Fe-based catalyst and ZSM-5 was developed by Wen et al.^241^ for selective CO_2_ hydrogenation to aromatics, where the catalytic mechanism of the prepared catalysts was investigated via different characterization techniques (Figure 9A and B). It was illustrated that the Cu promoter could enhance the carbon-chain growth, reduce methane formation over the Fe-based catalyst, and consequently boost aromatic selectivity in the bifunctional catalyst, where heavy olefin-based intermediates were augmented to aromatization compared to lighter ones. It was further interpreted that by increasing the straight channel length of the zeolites along the *b*-axis direction, a space time yield (STY) of toluene of 14.8 g_CH2_·kg_cat_^–1^·h^–1^ could be achieved. This was attributed to the elongation of the *b*-axis length, which limited the diffusion of products along the straight channel and compelled them to diffuse through the sinusoidal channel in the ZSM-5 zeolite. However, copper addition can also lead to surface defects. For example, Chen et el.^34^ determined that the addition of copper (Cu) to sodium-modified iron oxide (Na–Fe_3_O_4_) shows a synergistic effect for enhancing the creation of O_v_, where it enhances the removal of surface oxygen atoms and increases the formation of O_v_. This enhancement was particularly notable when sodium was present, as indicated by the shift in the reduction temperature toward the lower position required to convert iron oxide (Fe_2_O_3_) to Fe_3_O_4_ after Cu doping. This finding suggested that Cu has a more pronounced effect on surfaces with sodium, leading to the removal of more oxygen atoms and the creation of more O_v_. Additionally, Cu doping further increases the presence of COOH* intermediates, promoting the nondissociative activation of CO, resulting in increased gaseous CO production while reducing the surface content of CH_x_*, and preventing excessive C–C coupling, thus enhancing the production of lower olefins. We have also deeply investigated the impact of different combinations of Cu and Co components in Fe–Co/Fe–Cu bimetallic systems on the catalytic performance of an integrated Fe-based/HZSM-5 catalyst in our recent studies of the STA process.^61,62^ The results showed that the codoping of Cu and Co metals at appropriate calcination temperatures effectively incorporated these elements into the Fe structure. This process facilitated the creation of numerous surface oxygen vacancies through crystal plane manipulation, tailored lattice distortion, and the formation of multiple-phase interfaces (Figure 9C). The fabrication of finely tuned CoFe_2_O_4_ bimetallic nanocrystals enhanced the surface’s adsorption capabilities, influencing the reduction behavior of various metal oxides (Fe, Cu, and Co). The synergistic interaction between Cu and Co, along with increased oxygen vacancy concentration, promoted CO chemisorption and CO_2_ molecule dissociation during the reaction, enhancing the catalyst’s intrinsic activity.

**Figure 10:**
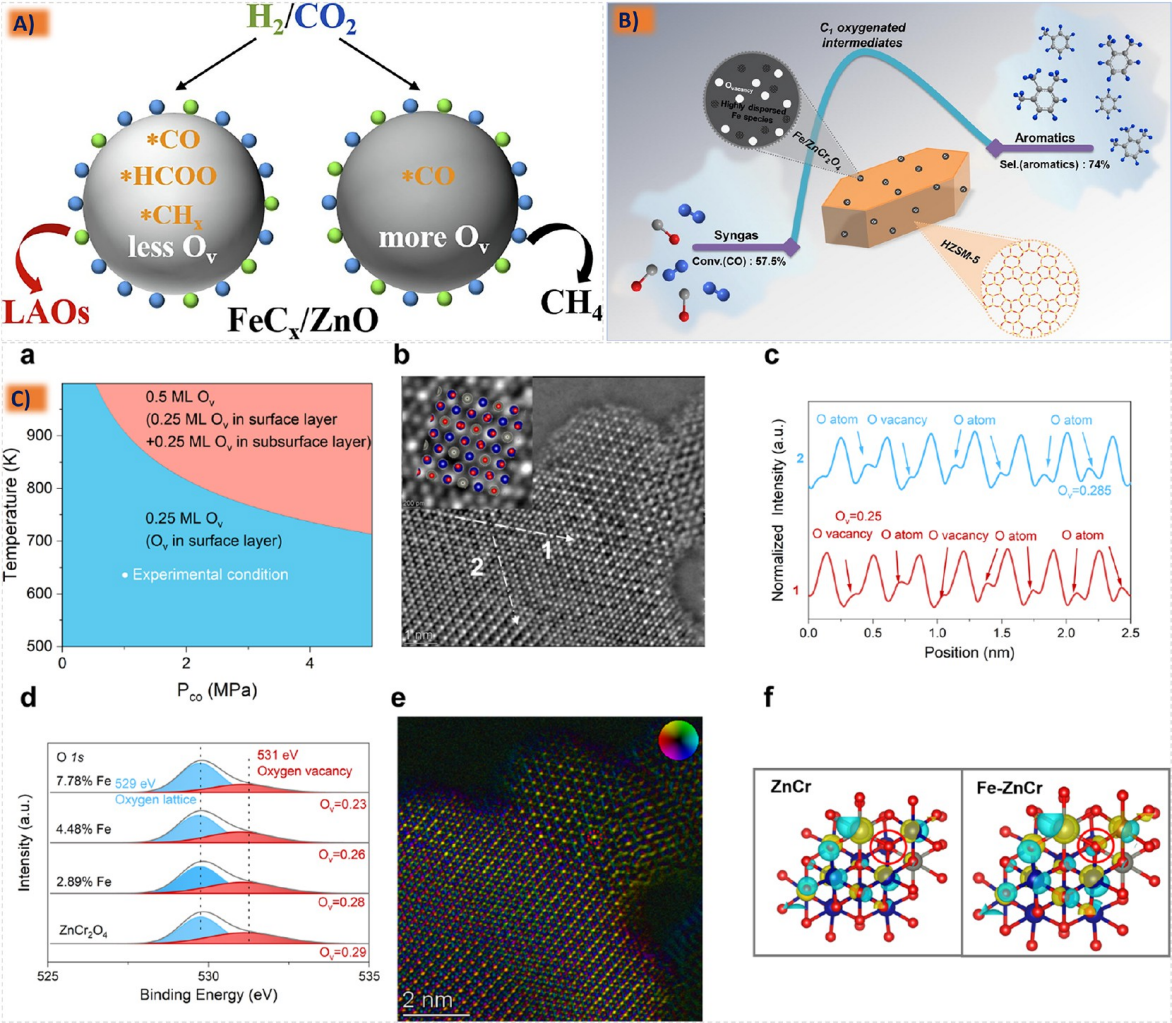
(A) Effects of surface oxygen vacancies for linear α-olefins on FeCx/ZnO catalysts. Reproduced with permission from ref (160). Copyright 2023 Elsevier. (B) The synergistic effect of oxygen vacancy concentration and highly dispersed Fe carbides over Fe/ZnCr_2_O_4_ spinel oxide for the rapid formation of abundant oxygenated intermediate species. Reproduced with permission from ref (106). Copyright 2022 Elsevier. (C) Effect of oxygen vacancy formation as oxygen-donor support on the spontaneously monodispersed Fe in ZnCr_2_O_4_ spinel. Reproduced with permission from ref (30). Copyright 2022 Springer Nature.

**Figure 11:**
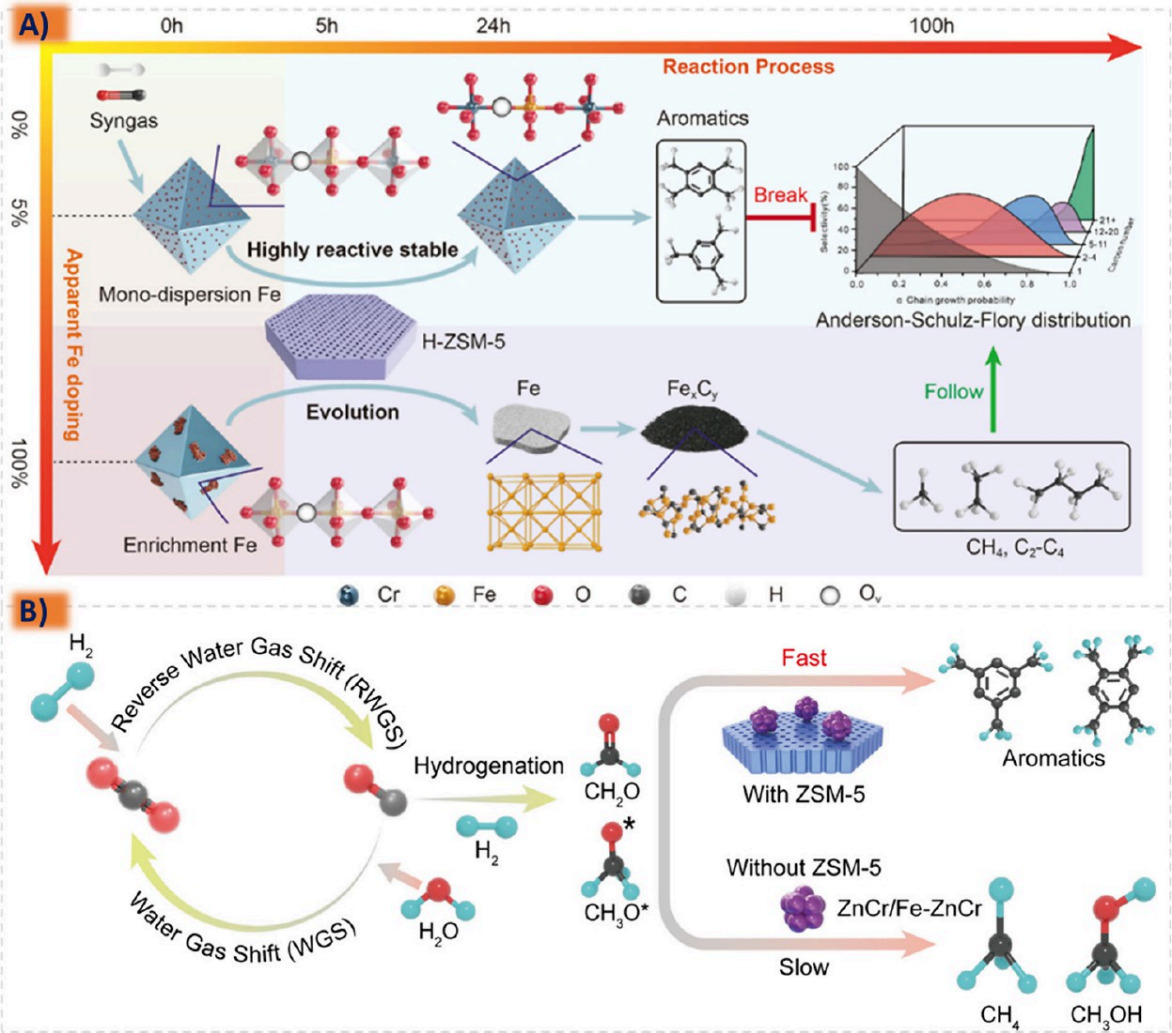
(A, B) Mechanistic insights on the effect of oxygen vacancy and Fe-carbide actives dictating different reaction intermediates on the spontaneously monodispersed Fe in ZnCr_2_O_4_ spinel. Reproduced with permission from ref (30). Copyright 2022 Nature Springer.

The intricate synergy between zinc and other metal oxides also emphasized the importance of precise adjustment of multiple catalyst additives to optimize the overall catalytic performance (Figure 10A).^160^ Zn has found its potential role in reducing carbon deposition and improving the stability of molecular sieves in FeMn-based catalysts composited with HZSM-5, where the stronger Brønsted acid sites in a lower SiO_2_/Al_2_O_3_ ratio of HZSM-5 linearly promoted the aromatics selectivity.^69,242^ Doping zinc into a binary Fe–Al spinel could result in a ternary Fe–Zn–Al spinel catalyst, achieving a noteworthy space-time yield for linear α-olefins in CO_2_ hydrogenation reactions; active Fe_5_C_2_ nanoparticles could be enveloped with Fe–Al spinel layers upon the introduction of aluminum alone, facilitating hydrogenation while impeding C–C coupling over the Fe–Al catalysts.^212^ Additionally, zinc doping allowed for the redistribution of aluminum, mitigating undesirable strong interactions between Fe_5_C_2_ and spinel phases and resulting in heightened selectivity to higher olefins. Fu et al.^106^ devised a novel catalyst that effectively transforms syngas into valuable aromatic hydrocarbons by integrating iron-modified Fe/ZnCr_2_O_4_ with zeolite, which remarkably achieved a notable higher (57.5%) CO conversion with a high (74%) aromatics selectivity (Figure 10B). The catalyst’s success lies in the impregnation of a small amount of iron (3%) into ZnCr_2_O_4_, finally transforming the Fe/ZnCr_2_O_4_ spinel oxide into the highly dispersed iron carbide species during the reaction. The synergistic effect of increased oxygen vacancies in the spinel ferrite enhanced the CO molecule activation, while the iron carbide species formed during the reaction further bolstered the process. These elements work together to promote the creation of intermediate compounds easily converted into aromatics by zeolite, thus providing significant insights into crafting highly efficient catalysts for syngas conversion. Similarly, Gu et al.^243^ studied the effectiveness of K and Fe loaded onto ZrO_2_ compounds (Fe-K/ZrO_2_) in CO_2_ hydrogenation depending on the presence of O_v_, which varies due to the different crystal phases of ZrO_2_. Using impregnation methods, K and Fe were applied to the surfaces of monoclinic zirconia (m-ZrO_2_) and tetragonal zirconia (t-ZrO_2_), with Fe levels ranging from 0 to 20 wt % and K at 1 wt %. The enhanced activity of 10Fe-1K/m-ZrO_2_ was primarily due to the higher concentration of surface oxygen vacancies on m-ZrO_2_ following the reduction in H_2_/N_2_. However, excessive surface Fe species on m-ZrO_2_, reducing the concentration of surface O_v_, would also hinder ZrO_2_’s catalytic performance in CO_2_ conversion. A similar effect was also investigated by Orege et al.^244^ demonstrating that both strontium and sodium exhibit synergistic effects in increasing the number of O_v_, where the characteristic surface CO_3_*, HCO_3_*, and HCOO* species generated from surface O_v_ were apparently filled through the interaction between CO_2_ and OH groups. Thus, the increased CO_2_ adsorption capacity within the Na–Sr–Fe catalyst enhanced the CO_2_ conversion rates while maintaining low selectivity for CO and CH_4_ (both below 10%). In another report by Tian et al.,^30^ a rationalized ZnCr_2_O_4_ spinel as an oxygen-donor support was synthesized to spontaneously monodisperse Fe on ZnCr_2_O_4_ with abundant O_v_ via a simple impregnation method for the syngas-to-aromatic reaction by coupling the monodispersed Fe with a H-ZSM-5 zeolite (Figure 10C). A record high selectivity of total aromatics (80–90%) at a single pass and a turnover frequency from 0.14 to 0.48 s^–1^ were achieved by monodispersed Fe, ascribed to more efficient activation of CO and H_2_ at the oxygen vacancy closest to the isolated Fe site and the prevention of carbide formation, which could lead to different catalytic behaviors (fast or slow) in WGS and RWGS routes (Figure 11A and B). The role of oxygen vacancies within Fe-based catalysts is nothing short of remarkable when it comes to wielding a multitude of roles for converting syngas into aromatics. They can serve as bustling active sites that determine the CO/CO_2_ absorption/dicossiation rates while delicately modifying the catalyst’s electronic structure, ensuring a steady performance over time, opening up avenues for fine-tuning and optimization, and providing an exciting prospect for crafting catalysts that are not just efficient but exquisitely selective. In essence, they are the unsung heroes in the realm of FTS catalyst development that can pave a harmonious way for the synthesis of precious aromatic compounds from syngas.

#### 2.6.5 Bimetallic (Fe–Co) FT Components as the Activity Enhancement Aspirants

**Figure 12:**
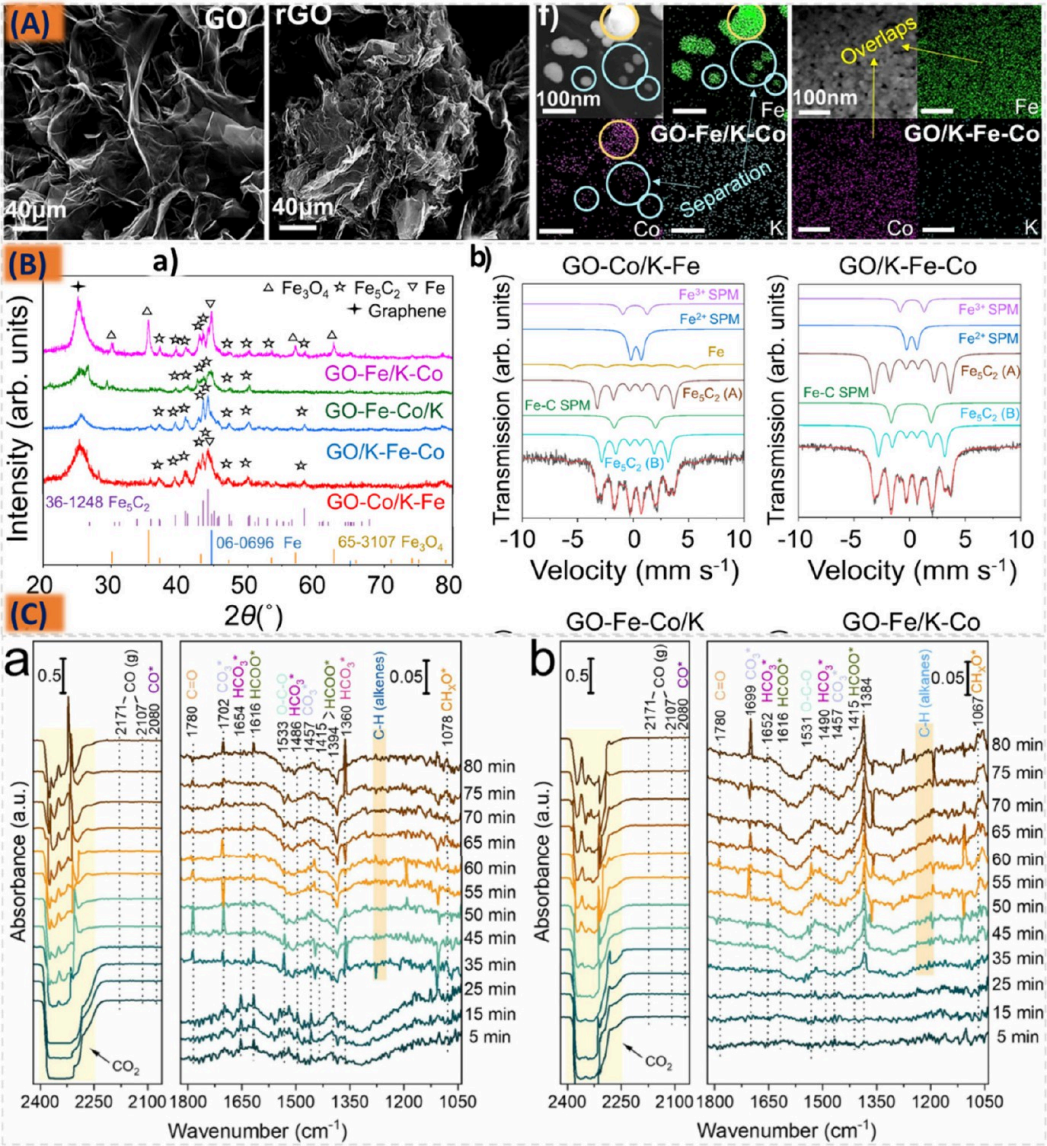
(A, B) SEM, XRD, and MES results of Fe–Co bimetallic catalysts for CO_2_ hydrogenation with tunable selectivity through a graphene oxide and reduced graphene oxide fencing approach. Reproduced with permission from ref (252) 2024 Springer Nature. (C) In situ high pressure DRIFTS of highly efficient Co–Fe bimetallic catalysts for CO_2_ hydrogenation into olefins. Reproduced with permission from ref (253). Copyright 2023 Elsevier.

Numerous research endeavors concentrate on bimetallic catalysts that amalgamate conventional FTS transition metals, aiming to address the limitations associated with single-metal catalysts, where Fe and Co emerge as the most utilized composite catalysts. Investigations in this domain have demonstrated that the combination of these metals yields heightened catalytic activity, attributed to synergistic geometric and electronic effects, that surpasses those observed with pure (Fe or Co) metal catalysts in FTS processes.^245^ Different reports examining the interaction between cobalt and iron species in alumina-supported bimetallic catalysts revealed that the incorporation of a minor quantity of cobalt alongside iron enhanced the reducibility of iron.^246^ This adjustment in catalytic behavior facilitates an increased conversion of carbon oxides, leading to a significant enhancement in the selectivity of light olefins. In parallel investigations, the structural and electronic characteristics of cobalt–iron bimetallic catalysts unveiled that the synergistic influence arising from the presence of cobalt alongside iron species significantly enhanced catalytic activity, which was particularly notable in the selective synthesis of a diverse spectrum of hydrocarbons.^247,248^ Furthermore, certain investigations have examined the employment of zeolite as a supporting material in the Fe–Co bimetallic catalytic system for FTS, observing an enhanced and consistent catalytic performance, particularly toward lower olefins and aliphatic hydrocarbons within the C_13_–C_23_ range.^124,249,250^ However, the product distribution and catalytic behavior of both metals in FTS depend on various factors such as loading amounts, preparation methods, operating conditions, and catalyst support.^94,216,251^ Despite extensive research efforts over past decades to develop efficient bimetallic Fe–Co catalysts for FTS, particularly for linear hydrocarbon production, only a few early studies have investigated the integration of iron–cobalt bimetallic catalysts with zeolite for the direct conversion of syngas to aromatic hydrocarbons. Martínez et al. pioneered the development of a hybrid catalyst in 2005, comprising a physical mixture of bimetallic KFeCo and ZSM-5 zeolite, for one-step conversion of syngas to gasoline-range isoparaffins and aromatics. Although this catalyst exhibited an overall aromatics selectivity of 34%, it suffered from progressive deactivation, with a time on stream (TOS) of only 17 h.^135^ Moreover, achieving the desired catalytic behavior for aromatic synthesis with Fe-rich catalysts requires higher reaction temperatures compared with Co- or Cu-rich examples. Matching the operating variables for Cu or Co metals is crucial for achieving the desired catalytic behavior in aromatic synthesis.^128,248^ However, the combination of both Fe and Co as FTS main active components is gaining momentum again as the activity enhancement interacts with substantial geometrical and electronic modifications.^238,250^ Liang et al.^252^ introduced a novel method involving graphene fence engineering to control multiple reaction sites (Figure 12A and B). By utilizing Fe–Co active sites on the graphene fence surface, the process achieves high selectivity for light olefins (50.1%) and liquefied petroleum gas (43.6%). This approach sets a precedent for CO_2_ direct hydrogenation to liquefied petroleum gas via the Fischer–Tropsch pathway, boasting superior space-time yields compared to other composite catalysts reported previously. Liu et al.^253^ delved into the structural changes, proposed distinct adsorption properties, and surface reactivity of various Na-promoted Co–Fe catalysts with different compositions and proximities using advanced characterization techniques. The overall findings suggested that Co_1_Fe_2_ is more selective for olefins in comparison to a single-atom Co catalyst that would favor methane formation methane. An in situ diffuse reflectance infrared fourier transform (DRIFTS) study (Figure 12C) revealed that the CoFe bimetallic catalyst facilitates the formation of carbonate, bicarbonate, and formate species from CO_2_, leading to key intermediates for olefin production with higher CO_2_ conversion levels.

**Figure 13:**
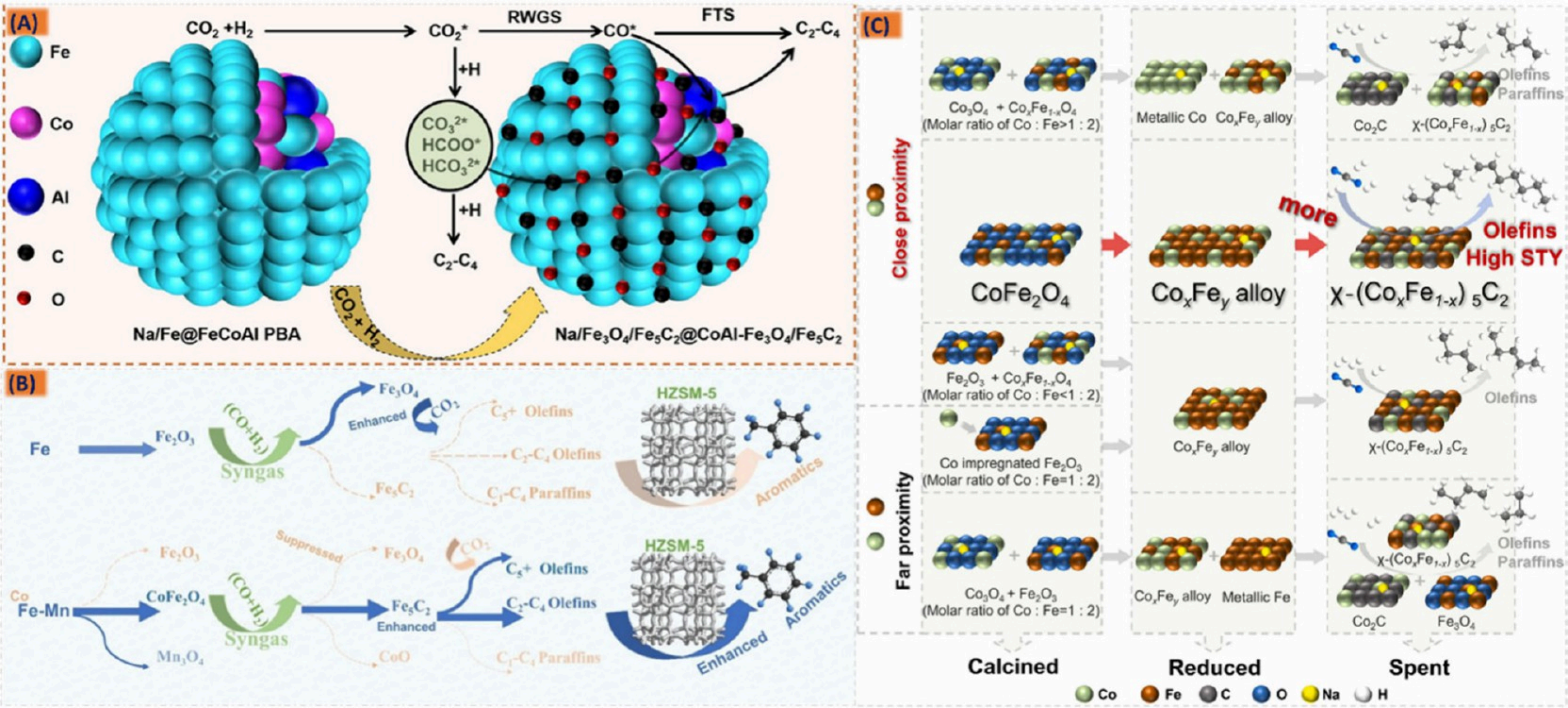
(A) FeCoAl Prussian Blue analogue-based core–shell catalyst for directly converting CO_2_ to light hydrocarbons via Fischer–Tropsch synthesis. Reproduced with permission from ref (254). Copyright 2023 American Chemical Society. (B) Synergistic interplay of Fe–Co bimetallic oxides for the different catalytic behaviors of Fe_2_O_3_/CoFe_2_O_4_/MnCo_2_O_4_ species in a Na-FeMnCo/HZSM-5 composite catalyst. Reproduced with permission from ref (61). Copyright 2021 Chemistry Europe. (C) Schematic structural evolution of highly efficient Co–Fe bimetallic catalysts for CO_2_ hydrogenation into olefins. Reproduced with permission from ref (253). Copyright 2023 Elsevier.

Similarly, a distinct reaction scheme has been proposed by Li et al.^254^ for CO_2_ FT synthesis to C_2_–C_4_ hydrocarbons over a novel porous FeCoAl Prussian Blue analogue (PBA)-based core–shell catalyst (Na/Fe@FeCoAl-P) with dual-active interfaces (Figure 13A). The model catalytic system (Na/Fe@FeCoAl-P0.1) exhibited significantly improved selectivity (40.8%) for C_2_–C_4_ hydrocarbons, a high olefin/paraffin (O/P) ratio of 8.7, and a markedly enhanced CO_2_ conversion of 54% at 330 °C compared to the C_2_–C_4_ hydrocarbon selectivity of 32.0% at a CO_2_ conversion of 38.1% of a traditional Na/Fe catalyst. Additionally, the selectivity of C_2_–C_4_ hydrocarbons reaches 45.7%, with 38.7% comprising C_2_^=^–C_4_^=^ products at 280 °C. Characterization results revealed that the presence of FeCoAl PBA not only restrained the production of heavy hydrocarbons due to its unique porous structure but also promoted the formation of an abundant Fe_5_C_2_ phase, enhancing FTS activity. Here, apart from the activated CO_2_* and CO* species being transformed at the interfaces between Fe_3_O_4_ and Fe_5_C_2_ originating from the Fe shell, the remaining CO_2_* and CO* species diffuse from the FeCoAl core to the CoAl-promoted Fe_3_O_4_/Fe_5_C_2_ interfaces, subsequently undergoing additional hydrogenation and C–C coupling processes. Meanwhile, in our recent study of Fe–Co bimetallic composite catalyst containing Na-FeMnCo/HZSM-5, we thoroughly investigated the effect of modulating different calcination temperatures and ratios of Fe, Mn, and Co on the catalyst’s composition of Fe_3_O_4_, Fe_*x*_C, and Co_*x*_C reactive species, which resulted from divergent Fe_2_O_3_, CoFe_2_O_4_, and MnCo_2_O_4_ structural variants^61^ (Figure 13B). Further insights demonstrated that incorporating Co and Mn into Fe led to structural and electronic adjustments, resulting in uniformly distributed CoFe_2_O_4_ nanocrystals and influencing the formation of oxygen vacancies, reduction, and carburization behaviors that ultimately enhanced the concentration of the active Fe_5_C_2_ phase. The catalyst showed improved selectivity toward olefinic intermediates and higher aromatic fractions of 55% at a CO conversion rate of approximately 98% with reduced CO_2_ generation.

The proposed reaction scheme by Liu et al.^253^ delving into the structural changes of various Na-promoted Co–Fe catalysts with different compositions involves CO_2_ activation and conversion to CO* on the χ-(CoxFe_1–*x*_)_5_C_2_ active site, with varying Co/Fe ratios influencing the pathway toward either olefins or methane (Figure 13C). It was discovered that the arrangement and content of Co and Fe in the catalysts can elucidate the intrinsic correlation between structural changes and olefin synthesis in CO_2_ hydrogenation. Here, the Co_1_Fe_2_ catalyst, characterized by a singular CoFe_2_O_4_ spinel structure in its precursor, displayed superior capability in rapid reduction and carbonization, transitioning from CoFe_2_O_4_ to the Co_*x*_Fe_*y*_ alloy and ultimately to χ-(Co_*x*_Fe_1–x_)_5_C_2_ (up to 97%). Consequently, it exhibited notably enhanced catalytic performance compared to the other catalysts studied, maintaining stability over a 500 h test period without significant deactivation and achieving an unprecedented olefin yield of 1810.8 mg·g_cat_^–1^·h^–1^, which is particularly suitable for microchannel reactors. Additionally, the alloy carbide promoted CO_2_ adsorption, hindered surface intermediate hydrogenation, and suppressed carbon deposition, highlighting its unique role in catalysis. However, Co/Fe molar ratios exceeding 1/2 hinder the formation of χ-(Co_*x*_Fe_1–*x*_)_5_C_2_ alloy carbide, favoring Co_2_C formation and resulting in poor olefin synthesis performance. Catalysts with weaker Co–Fe interactions, such as im-Co_1_Fe_2_ and phy-Co_1_Fe_2_, exhibit different extents of alloying for the Co_*x*_Fe_*y*_ and χ-(Co_*x*_Fe_1–*x*_)_5_C_2_ carbide phases, leading to inferior olefin synthesis performance compared to that of Co1Fe_2_. The Fe-based/HZSM-5 catalyst, as a composite comprising a typical bimetallic FT synthesis catalyst and a molecular sieve, necessitates careful consideration in the application of appropriate quantities and optimal process conditions for the STA process due to the pronounced methanation activity of cobalt at elevated temperatures. On the other hand, excessive addition of cobalt content may lead to heightened CH_4_ selectivity in the product, consequently diminishing the selectivity toward aromatics. This aspect may have been overlooked for decades regarding its utilization in composite catalysts for the STA process. This oversight likely stems from the predominant focus in prior studies on Fe–Co bimetallic catalysts, which typically manipulated catalytic activity with higher cobalt contents to achieve desired long-chain products under LTFT synthesis reaction conditions. Therefore, Fe–Co bimetallic oxides stand out as potent agents of activity enhancement for revolutionizing the Fischer–Tropsch synthesis reaction. Their unique composition imbues them with remarkable catalytic properties, making them pivotal players in driving the synthesis reaction forward and acting as frontrunners in the quest for optimized FTS. By combining the strengths of both iron and cobalt, these bimetallic oxides offer a synergistic effect that amplifies the catalytic activity, selectivity, and stability. Their significance lies not only in their ability to enhance the catalytic activity but also in their potential to shape the landscape of renewable energy production on a global scale, thus representing a beacon of hope for realizing the full potential of FTS in meeting the ever-growing demands of a modern, energy-hungry world.

## 3 Symphony of HZSM-5 and Its Tandem Reactions in Aromatic Hydrocarbons

**Figure 14:**
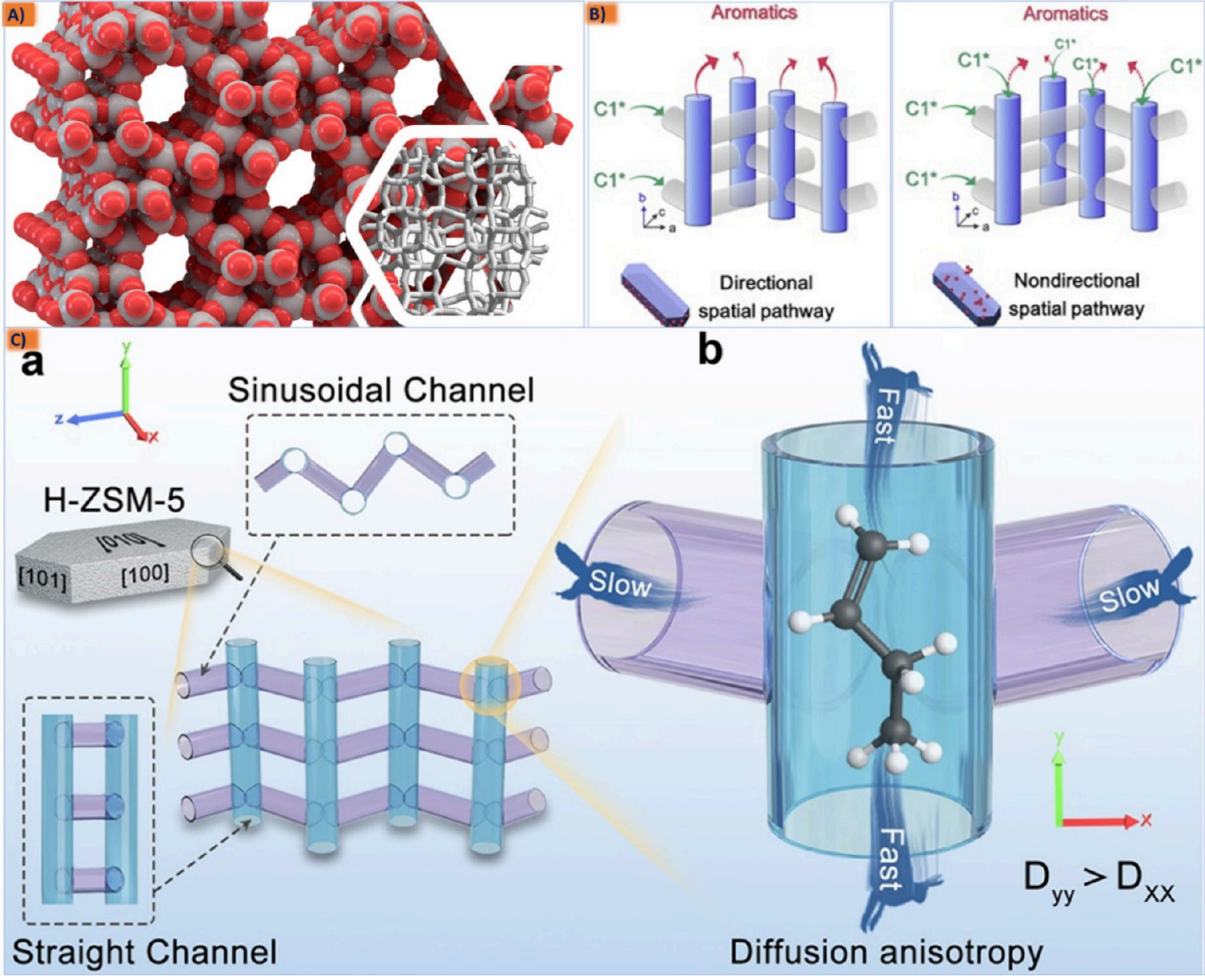
Schematic view of straight and sinusoidal channels along the different axes of hierarchical mesoporous, nano, or hollow H-ZSM-5 zeolites with different geometries, diffusion anisotropies, and channel and framework structures. (A) Reproduced with permission from ref (39). Copyright 2023 Springer Nature. (B) Reproduced with permission from ref (256). Copyright 2021 Elsevier. (C) Reproduced with permission from ref (257). Copyright 2022 Elsevier. (D) Reproduced with permission from ref (258). Copyright 2021 Nature Springer.

Zeolites are commonly defined as porous crystalline aluminosilicates constructed from tetrahedrally linked SiO_4_^–^ and AlO_4_^–^ units, containing high purity levels and the flexibility to modify most structures to fulfill specific functions. Shape-selective catalysis (typically zeolites) involves the manipulation of the interaction between the catalyst structure and the configurations/dimensions of reactants, intermediates, and products.^255^ This manipulation enables control over selectivity toward desired products or the pathways of catalytic reactions. Hence, HZSM-5 zeolite acting as a molecular sieve serves as a critical component in the tandem catalyst system that enables the selective and efficient production of valuable aromatic hydrocarbons from syngas by selectively filtering and transforming intermediate olefins (alkenes) into the desired aromatic products. Its unique microporous structure allows only molecules of specific sizes and shapes to pass through, directing the reaction toward aromatization, while the inherent acidity of HZSM-5 provides the necessary catalytic sites to facilitate the complex chemical transformations involved in the process (Figure 14).^39,256−258^ HZSM-5 zeolite is a workhorse in the realm of catalysis, which owes its exceptional activity in transforming olefins into aromatics to the unique interplay of two types of acidic sites: Brønsted and Lewis acid sites. Brønsted acid sites, essentially protons (H^+^) attached to framework oxygen atoms, act as proton donors, initiating and facilitating the crucial carbon–carbon bond formations required for aromatization, while Lewis acid sites, formed by vacant electron orbitals on metal cations like aluminum (Al^3+^) within the zeolite framework, act as electron acceptors, polarizing and weakening nearby carbon–carbon bonds and thus making them more susceptible to breakage and rearrangement during the reaction. This synergistic action of both acid types is crucial for the efficient conversion of olefins into aromatic rings. Brønsted sites initiate and drive the key reactions, while Lewis sites assist by weakening bonds and stabilizing intermediate structures, finally orchestrating the transformation of simple olefins into complex and valuable aromatic hydrocarbons. Therefore, HZSM-5 molecular sieves could be regarded as the most used aromatization catalysts for syngas to aromatic hydrocarbons process in view of their standout features of uniform pores, tunable acidities, and high thermal and hydrothermal stabilities.^113,259,260^

### 3.1 Architectural Modifications (Core–Shell, Yolk Shell, or Eggshell Structures) for High Aromatic Yield

**Figure 15:**
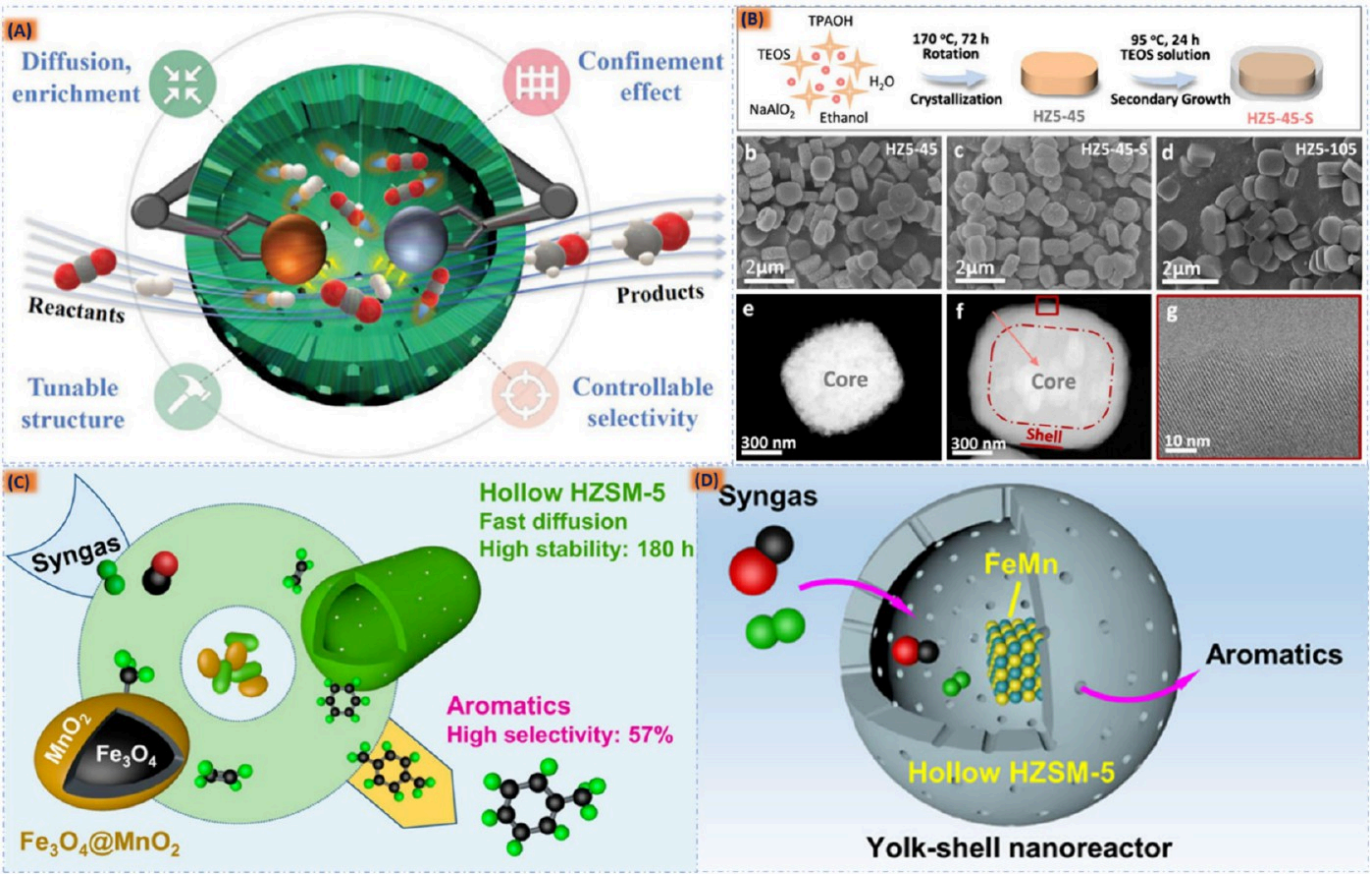
Different synthesis and confinement schemes of hollow, core, yolk, or egg shell structures of Fe-based/HZSM-5 catalysts. (A) Reproduced with permission from ref (262). Copyright 2020 John Wiley & Sons. (B) Core–shell. Reproduced with permission from ref (266). Copyright 2023 American Chemical Society. (C) Fe/Mn core–shell with hollow HZSM-5. Reproduced with permission from ref (127). Copyright 2019 American Chemical Society. (D) Fe/Mn yolk shell with hollow HZSM-5. Reproduced with permission from ref (269). Copyright 2021 American Chemical Society.

The characteristics of zeolite-supported metal catalysts, exerting control over the reaction products from the metal catalysts and serving as intermediates for secondary processing, could provide excellent activity, thermal stability, and shape-selectivity in diverse catalytic processes. Encapsulating a hierarchical, hollow, or hollow spherical dual-metal layer zeolite around FTS catalysts using a core–shell (CS), yolk shell (YS), or eggshell (ES) structure could enhance the selectivity and modulate the final product distributions^261,262^ (Figure 15A). The zeolite outer layers in the core–shell catalysts could facilitate the cracking and isomerization of long straight-chain hydrocarbon products, resulting in the suppression of C_11_ hydrocarbons and an increase in the ratio of isoparaffins/normal paraffins.^263,264^ Meanwhiole, the systematic assessment of the impact of mesoscopic gradients in acid site concentration for a series of CS MEL-type zeolites (highlighted in a recent study) revealed that ZSM-11@silicalite-2 particles with ultrathin shells have enhanced mass transport. Here, the ES configurations comprised of an inert core and a catalytically active shell in silicalite-2@ZSM-11 and silicalite-1@ZSM-5 created pseudo-nanosheets with total turnovers that were markedly higher than those of their homogeneous counterparts.^265^ Similarly, nanocatalytic architectures with advanced and complex YS structures (raspberry cores with a single multishelled core) have recently been regarded as a protective and performance-enhancing feature.^227,266^ The shape confinement effects encapsulate the active phase with a protective and porous shell material, and the larger void between the “yolk” and the metallic core prevents normal deactivation states from accumulating and sintering. The modifications to the shell, yolk, void, or a combination of these could provide excellent catalytic performance and stability via different design strategies, especially the seed-directed growth synthesis method.^267^ In a recent study on a microscopic scale, the proximity of two framework aluminum atoms in zeolite was demonstrated to lead the creation of paired acid sites, enhancing activity in various reactions like methanol to aromatics.^268^ Meanwhile, at the mesoscopic level, positioning aluminum atoms at crystal edges accelerates reaction progression and enables the engineering of hollow structures, significantly improving the aromatic yield. By exploiting this aluminum zoning, the hollow zeolites fabricated through desilication could result in the highest capacity for aromatic production. The comparative catalytic performance of different H-ZSM-5 zeolites with similar properties but differing aluminum distributions illustrated that enriching aluminum closer to the external surface results in more active paired acid sites compared to isolated ones. This arrangement not only speeds up cascade reactions, leading to increased aromatic yields, but also improves diffusion and void-confinement properties. A recent study by Xing et al.^266^ underscores the efficacy of a shielding strategy in preserving the bifunctional nature of CO_2_ hydrogenation catalysts; however, the methanol-mediated pathway can also be encouraged toward the FT synthesis aromatic route (Figure 15B). A simple technique of coating a layer of silicalite-1 (S-1) outside H-ZSM-5 crystals has been proposed, where the S-1 layer (1) could limit metal migration while preserving H-ZSM-5 acidity, preventing excessive metal loss and prolonging catalyst lifetime by inhibiting the aromatic cycle under microscale proximity (granule mixing, GM) compared to the uncoated catalyst. Xu et al.^127^ introduced a novel bifunctional catalyst combining Fe_3_O_4_@MnO_2_ and hollow HZSM-5, which demonstrated a remarkable ability to synthesize aromatics from syngas with a selectivity of 57% at CO conversion exceeding 90% with excellent stability over a 180 h (Figure 15C). It was interpreted that the electron transfer between Mn and Fe species within the core–shell Fe_3_O_4_@MnO_2_ catalyst facilitated the generation of olefin intermediates, which subsequently adsorbed at the acid sites of HZSM-5 to form aromatics. The unique shortened channels and cavity structures of hollow HZSM-5 facilitated the diffusion of reactants and products, thereby enhancing catalyst stability by minimizing carbon deposition, while the closer proximity of reaction intermediates typically enhances the catalytic performance of bifunctional catalyst, as the short distances (proximity) between the different active sites of the FT phase and the hydroprocessing phase (HZSM-5) can be highly beneficial for accelerating the second reaction through a high local concentration of the product of the first catalyst, thus enhancing the selectivity. Although each generated variant has its advantages, no variant can be described as a superstructure because of the possible different needs for each chemical process, thus necessitating the different generated sites tailored to the reaction. A novel catalyst comprising FeMn nanoparticles as the yolk and hollow HZSM-5 zeolite as the shell, developed by Xu et al.,^269^ exhibiting a remarkably high aromatics space-time yield (STY) of 1.9 g·gFe^–1^·h^–1^, surpassing those of the previous catalytic systems by a significant margin (Figure 15D). The superior catalytic performance stemmed from the ordered yolk@shell structure, which effectively utilized the shape-selective effect and aromatization capability of the HZSM-5 zeolite. By tuning the elemental composition of the yolk, along with the hollow structure, the mesoporous channels, and the acidity of the nanoreactor, we could enhance aromatics production while mitigating coke deposition. These findings offer a promising strategy for fabricating highly efficient multifunctional catalysts that may find application in other tandem catalysis systems.

### 3.2 Architectural Modifications in HZSM-5 (Mesoporous, Nano, Hollow, or Hierarchical Designs) for Dictating the Reactions toward BTX

The tailored architecture of HZSM-5 guides the reactions, resulting in improved yields and minimizing the formation of undesired byproducts, a pivotal aspect in sustainable synthesis. The integrated catalyst system optimizes this property, enhancing the selectivity toward the desired aromatic products.^148,259,270^ However, the conventional HZSM-5 zeolite possesses a microporous structure, limiting the diffusion of larger molecules, such as those formed during syngas conversion. To address this, researchers have explored the synthesis of different variants of HZSM-5 including mesoporous, nanoporous, hollow, hierarchical, core–shell (CS), eggshell (ES), and yolk shell (YS) HZSM-5. In here, mesoporous ZSM-5 boasts a larger pore size compared to conventional ZSM-5, offering improved accessibility for bulkier intermediate products generated during syngas conversion. This enhanced accessibility can potentially lead to increased conversion efficiency and product yields of aromatic hydrocarbons. Various methods are employed for the synthesis mesoporous ZSM-5, often involving the use of templates or specific chemical treatments to create additional mesopores within the zeolite structure, while nano-ZSM-5 features smaller crystals compared to conventional ZSM-5, resulting in a higher external surface area. This increased surface area provides more active sites for the desired catalytic reactions, potentially leading to improved conversion rates and aromatic hydrocarbon selectivity. Synthesis methods for nano-ZSM-5 typically involve modifying reaction conditions or utilizing specific precursors to control crystal growth and size. Both mesoporous and nano-ZSM-5 present promising avenues for enhancing the performance of ZSM-5 catalysts in syngas conversion to aromatic hydrocarbons. Their specific advantages in terms of improved mass transport and increased active sites hold significant potential for optimizing this process toward more efficient and sustainable production of valuable chemicals. Compared with microporous ZSM-5, mesoporous ZSM-5 has a high specific surface area, a large pore size, and good catalyst stability and is beneficial to the shape-selective effect of aromatic hydrocarbons. Mesoporous ZSM-5 can be obtained by some specific templates or alkali treatment of microporous ZSM-5.^271^ Zhou et al.^125^ and Song et al.,^272^ using NaOH to treat HZSM-5 by ion exchange and impregnation methods, not only changed the acidity of the molecular sieve but also changed the pore structure of the molecular sieve, making the catalyst less prone to carbon deposition while prolonging its life. The mesoporous ZSM-5 was synthesized using three different templating agents, CTAB, DDAC, and CNT, and the effect of the templating agent was investigated on the catalytic activity. The results indicated that the mesoporous molecular sieve synthesized using CNT as a template had higher aromatic selectivity and less carbon deposition during the reaction.

**Figure 16:**
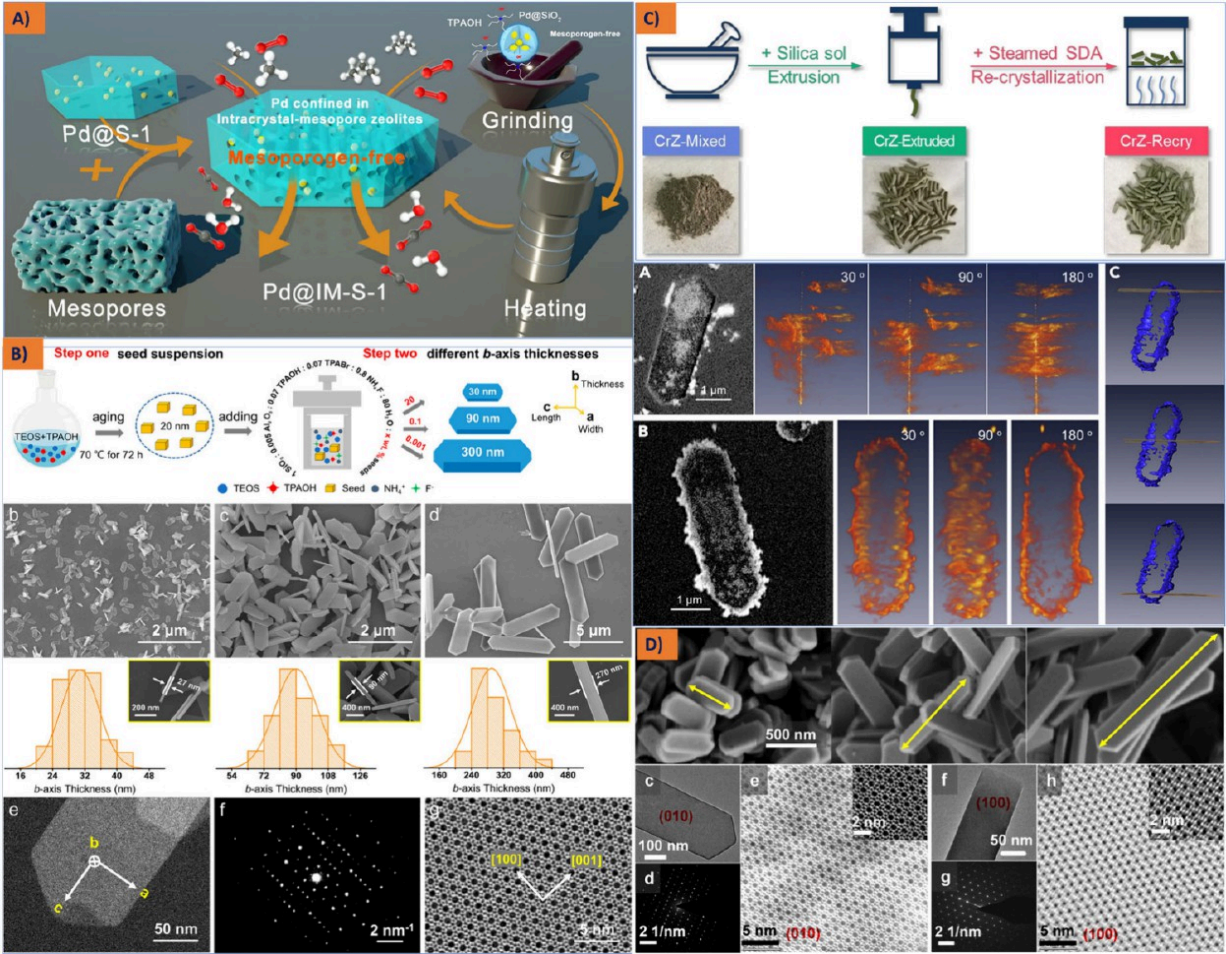
Synthesis schemes of different mesoporous, nano, or hollow zeolites, (A) Reproduced with permission from ref (274). Copyright 2022 Springer Nature. (B) Reproduced with permission from ref (275). Copyright 2023 American Chemical Society. (C) Reproduced with permission from ref (256). Copyright 2021 Springer Nature. (D) Reproduced with permission from ref (258). Copyright 2021 Spring.

Catalytic reactions in a simple FT catalyst would encounter challenges due to the strong adsorption of product molecules on the catalyst surface hindering continuous substrate conversion, which can be addressed by creating nanochannels for rapid product escape. Well-designed MFI or hierarchical zeolite crystals with specific dimensions and microporous or mesoporous environments can also find a role as promising promoters, helping accelerate product escape from the catalyst surface and thus boosting FT synthesis products such as olefins/gasoline and CO conversion in contrast to the solo Fe-based catalyst.^16,273^ The interaction between the environment and reaction intermediates is important for exploring catalytic mechanisms, where delving into acid–base catalysis in the case of zeolite would explore the functionalities and active sites critical for mediating the importance of surface acidity, basicity, and bifunctional sites in promoting catalytic transformations.^263^ Different reaction pathways would be evolved with the manipulation of surface acidic environments, different morphological and architectural effects, and different channel orientations. In contrast, a straightforward two-step in situ method was introduced by Peng et al.^274^ without the need for mesoporogens to produce palladium (Pd) nanoparticles (NPs) enveloped within a single-crystalline zeolite (silicalite-1, S-1) with intramesopores, termed Pd@IM-S-1 (Figure 16A). Here, the excellent catalytic activity was attributed to the zeolite shell’s confinement and guarding effect, along with enhanced mass-transfer efficiency and access to active metal sites. The interfaces between Pd and PdO serve as novel active sites, providing active oxygen species for the initial C–H cleavage of light alkanes, while the high thermal and hydrothermal stability and recyclability of Pd@IM-S-1 were attributed to the guarding effect of the S-1 zeolite shell, where the uniform mesoporous structure and nanosized crystals of the zeolite shell could enhance mass transfer efficiency and access to active sites. Similarly, in another study highlighting the significant application of *b*-axis-oriented ZSM-5 nanosheets in the alkylation of benzene with methanol, different thicknesses of nanosheets (approximately 30, 90, and 300 nm) were prepared to investigate their catalytic properties in alkylation reactions^275^ (Figure 16B). Here, the enhanced catalytic activity for xylene selectivity and extended lifetime of the 30 nm thick sample were attributed to the shortened channel length, increased surface area, and enlarged mesopore volume, facilitating reactant and product diffusion, enhancing acid site accessibility, and reducing coke formation. The precoking and heteroatom introduction strategies were developed to precisely adjust catalyst acidity while elucidating the diffusion effect on ethylbenzene selectivity over ZSM-5 nanosheets of different thicknesses, underscoring the role of strong Brønsted acid sites in modulating ethylbenzene selectivity dynamics. Effectively managing the formation, diffusion, and conversion of bridging intermediates is crucial for enhancing the efficiency of tandem reactions using multicomponent catalysts. However, conventional mechanical mixing often leads to a random distribution of catalytic components, resulting in inefficient diffusion and decreased catalytic performance. Liu et al.^256^ validated the possibility of tailoring the tandem reaction path by adjusting the spatial distribution of a bicomponent catalyst, offering insights for other bifunctional catalytic systems where the anisotropic diffusion of intermediates and products was crucial (Figure 16C).

Different research approaches in this regard also focus on metal modification to suppress one acidic site (Lewis or Brønsted) while compromising the other for selectively enhancing the desired product.^276^ The mesoporous ZSM-5 can also be directly synthesized by alkali treatment of microporous ZSM-5, where Zhao et al.^32^ applied FeZnNa/Z5 to the one-step synthesis of aromatics from syngas and studied the effect of alkali treatment and ion exchange modification on Z5. The results showed that the strong Brønsted acid sites of Z5 decreased and the aromatics selectivity of the reaction increased after NaOH treatment owing to the suitable acidity and pore structure. Similarly, the nano-ZSM-5 molecular sieve is advantageous in the aromatization reaction for its smaller crystal grains. Niu et al.^277^ investigated molecular sieves with different particle sizes, finding that molecular sieves with smaller crystallites had a better reaction effect, while in another study using a different method to prepare Zn-ZSM-5 they found that the grain size had little effect on the acidity of Zn/Z5. Here, the ion exchange method increased the Zn dispersion on the surface of the molecular sieve and affected the surface species with the formation of ZnOH^+^, which is beneficial to the improved aromatics selectivity and catalyst life.^278^ The modification of metal elements mainly affects the reaction performance by changing the acidic properties of ZSM-5 molecular sieves and improves the selectivity of aromatic hydrocarbons, usually by impregnation, ion exchange, chemical deposition, physical mixing, and skeletal element substitution. Therefore, the commonly used metal elements are transition metal elements such as Ga, Zn, Mo, Co, and Ni. Conte et al.^68^ found that Ni modification could increase the hydrogenation capacity of molecular sieves and favors the alkylation of toluene and methanol. Similarly, the aromatic products generated by Cu modification were mainly C_9_–C_11_ heavy aromatics, and Ag facilitated the formation of C_6_–C_7_ light aromatics. Meanwhiole, Agustín et al.^135^ and Yan et al.^59^ synthesized Pd-modified ZSM-5 catalysts and respectively found that the strong hydrogenation ability of Pd and higher temperature/pressure were beneficial to the aromatic compounds, with an increased catalyst service life. However, despite its initial effectiveness in response to the alteration in the HZSM-5 channels by those metals for the targeted selectivity of a specific hydrocarbon (such as *p*-xylene), it has been categorically illustrated that the metal-modified HZSM-5 compromised reactivity with selectivity due to modifiers acting as acid-site-neutralizing agents and partially obstructing pores, leading to reduced diffusivity of reactants, loss of active sites, decreased pore volume, and diminished surface area.^279^ Consequently, modified HZSM-5 demonstrated decreased specific activity and poor stability in prolonged reactions due to susceptibility to modifier loss. These challenges hinder the practical development of HZSM-5 catalysts with superior shape-selectivity for obtaining a highly purified targeted molecule with minimal energy consumption. An orientation-distributed bicomponent Cr_2_O_3_/ZSM-5 catalyst was developed using a straightforward method involving extrusion and recrystallization, primarily locating Cr_2_O_3_ on the (100) and (101) surfaces of ZSM-5 and leaving the (010) surface highly exposed, while the recrystallized catalyst exhibited unsaturated coordinated sites in Cr_2_O_3_ and a higher acid density in ZSM-5. This directional modulation of intermediate and product diffusion enhances the tandem reaction of the conversion of CO to aromatics in the recrystallized catalyst, achieving a formation rate of aromatic rings (on a carbon basis) of 2.99 mmol·g^–1^·h^–1^ at 49.4% CO conversion, marking the highest reported performance data to date. Meanwhile, an efficient synthesis scheme by Liu et al.^258^ demonstrated a range of H-ZSM-5 zeolites with similar sheet-like shapes but varying *c*-axis lengths, suggesting a metric to quantify the influence of morphology on the catalytic performance of H-ZSM-5 zeolite (Figure 16D). The samples with longer *c*-axes exhibited improved catalytic activity and stability, demonstrating that these differences arise from the varying diffusion behavior of olefins in different channels, where the proposed descriptor provided valuable insights into the design of highly efficient zeolite catalysts for olefin cracking.

**Figure 17:**
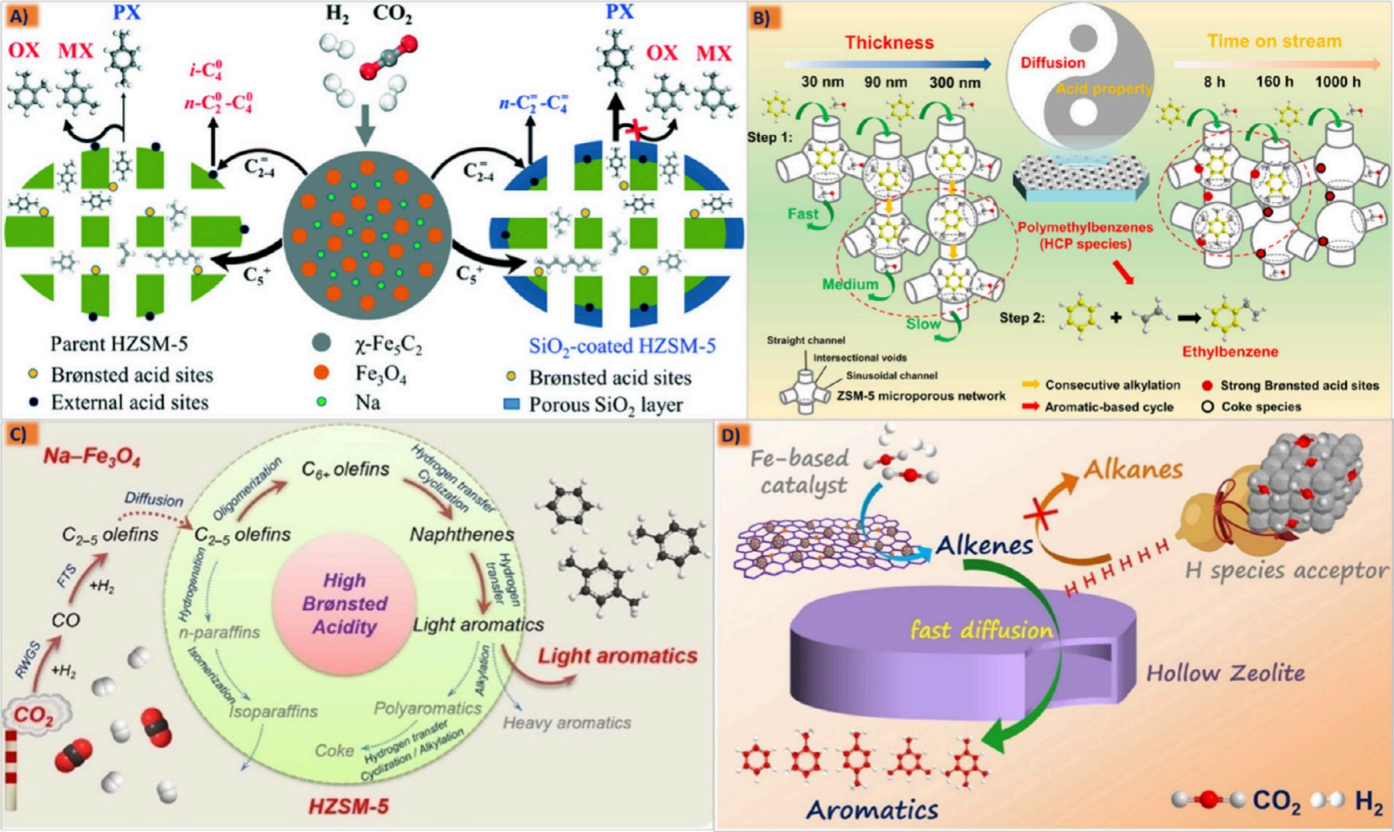
Symphony of HZSM-5 and its tandem reactions for light aromatic hydrocarbons by CO_2_ hydrogenation. (A) Reproduced with permission from ref (149). Copyright 2019 Royal Society of Chemistry. (B) Reproduced with permission from ref (275). Copyright 2021 American Chemical Society. (C) Reproduced with permission from ref (33). Copyright 2021 Elsevier. (D) Reproduced with permission from ref (153). Copyright 2020 Elsevier.

Similarly, coating the zeolite’s external surface with SiO_2_ significantly increases the selectivity of *p*-ethyltoluenes, suggesting that isomerization of initially formed *p*-xylene and *p*-ethyltoluene within zeolite channels is substantially reduced with diffusion onto the SiO_2_-coated external surface^149^ (Figure 17A). Furthermore, the SiO_2_ coating exhibited minimal impact on the intrinsic formation before diffusion out of the channels; conversely, severe isomerization could occur on the parent zeolite’s external surface due to exposed acid sites. Zhang et al.^275^ elaborated that the selectivity of ethylbenzene can be influenced by both diffusion and acidity factors, where the thickness of ZSM-5 nanosheets could directly dictate ethylbenzene selectivity at the initial stage of reaction due to varying diffusion behavior (Figure 17B). Thicker nanosheets with longer channels hinder benzene diffusion into micropores, leading to a higher methanol/benzene ratio in micropores. Additionally, thicker nanosheets slow down product diffusion, promoting consecutive alkylation and poly(methylbenzene) formation and thus increasing ethylbenzene selectivity. However, as the reaction progressed, changes in ethylbenzene selectivity were primarily driven by acidity, where strong Brønsted acid sites, which promoted methanol to ethylene side reactions, became covered by coke species over time, leading to decreased ethylbenzene selectivity and increased methanol utilization. The catalytic performance of the zeolite as an upgrading phase mainly depends on the balance between the hydrogenation ability, which is significantly influenced by the nature, accessibility, strength, and concentration of acid sites (Brønsted or Lewis).^263^ The cracking and hydrocracking abilities of zeolite have been reported to decrease in response to the decrease in acid site concentration with an increase in the Si/Al ratio. While the lower Si/Al ratio representing the higher acidity of zeolite could enhance hydrogenation function and moderate cracking activity, it may deactivate quickly due to the initial higher throughput of the acidic sites, leading to the coverage of the pores with carbon deposits. Brønsted acid sites (BAS) have been primarily proposed to drive the aromatization activity as the main active sites,^33,112^ where categorically increasing the BAS significantly enhanced the light aromatics selectivity (Figure 17C). Further illustration revealed that passivating the external BAS through silylation suppresses the alkylation and isomerization of light aromatics and xylene, with overall light aromatics constituting up to 75%. However, an intensive increase in BAS density (>154 μmol g^–1^) could also lead to accelerated coke formation, resulting in carbon-rich, highly condensed, and hard-to-oxidize coke species, thus deteriorating the catalyst’s properties and shortening its lifespan. However, the multifunctional catalyst combining Na-modified Fe-based catalysts with hollow acidic zeolite H-ZSM-5 ensures a high aromatic yield of 203.8 g_CH2_·kg_cat_^–1^·h^–1^ through the subsequent dehydrogenation and cyclization of formed alkenes on the acid sites of the hollow H-ZSM-5^153^ (Figure 17D). The cooperative interaction could be derived by the multifunctional catalyst components in the tandem process, where CO_2_ adsorbed on the Fe-based catalyst could act as an acceptor for H species generated from dehydrogenation and cyclization reactions, thereby enhancing aromatic yield by shifting the chemical thermodynamic equilibrium.

### 3.3 Mechanistic Insights for the Difference in Bifunctionalities of FTS and the MTS Route in the Tandem Approach

**Figure 18:**
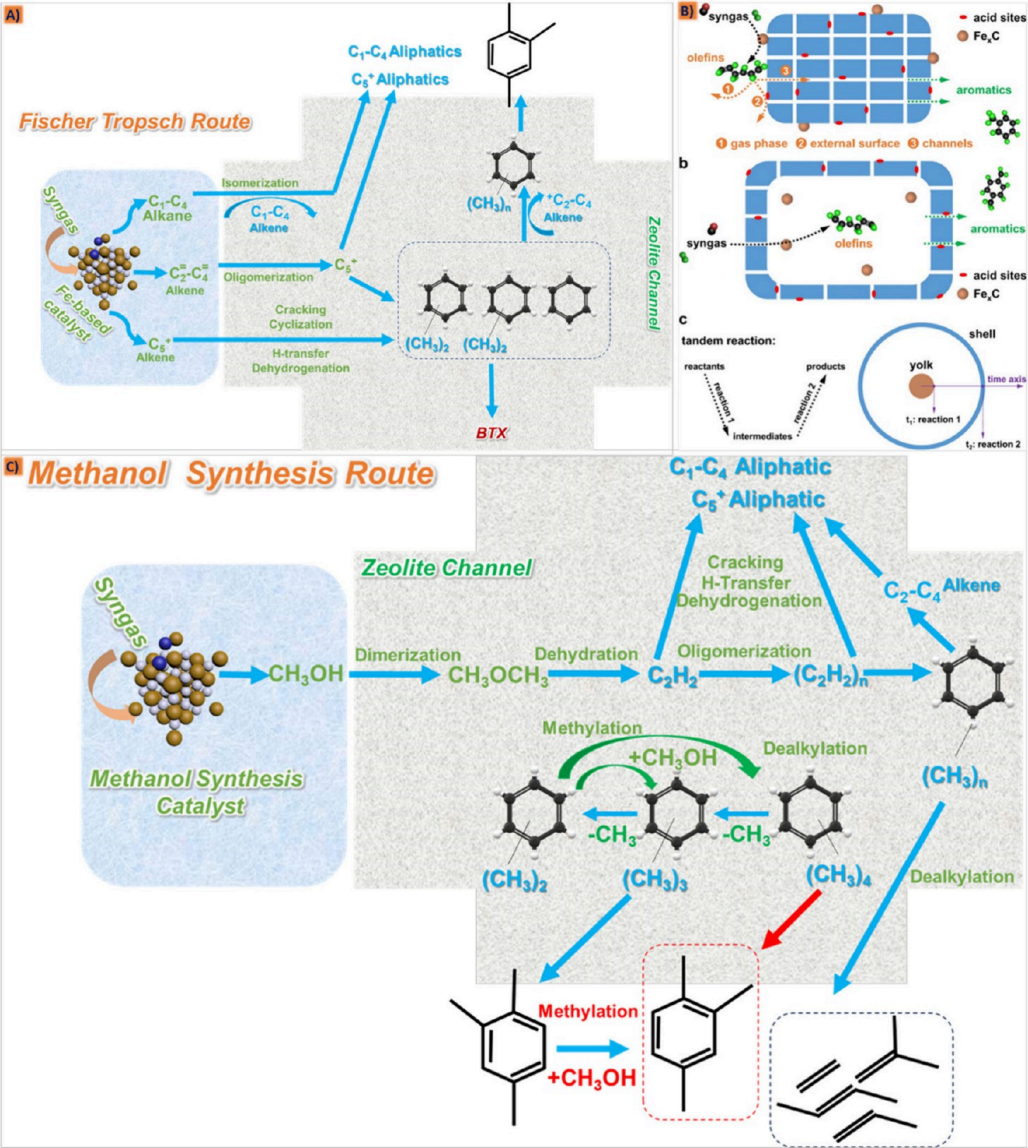
Differences between the asymmetry of (A, B) FTS and (C) the MTS tandem route on the HZSM-5 surface producing a different range of aromatic hydrocarbons. Reproduced with permission from refs (26) and (269). Copyright 2017 and 2021, respectively, American Chemical Society.

Under the action of molecular sieves, light olefins undergo a polymerization reaction to form long-chain hydrocarbons, and long-chain hydrocarbons are cyclized and dehydrogenated to form aromatic hydrocarbons. Aromatic hydrocarbons are also alkylated under the action of molecular sieves to form polyalkyl aromatic hydrocarbons, or dealkylated to produce low-carbon byproducts.^11^ It is generally believed that methanol will first adsorb on the acidic sites of solid acids and then dehydrate between the two molecules, generating DME.^195^ Methanol and DME then will be converted into light olefins on the molecular sieves, similar to the FT synthesis mechanism, where the main point depends on the formation step of the first C–C connection.^70,280^ A detailed mechanistic insight into STA process by FTS and MTS has been provided by Yang et al.^26^ and by Xu et al.^269^ (Figure 18A–C). It was demonstrated that the Fe-based/modified-HZ catalysts yield C_2_^=^–C_4_^=^ as well as C_5_^+^ alkenes through the FTS route on Fe_2_O_3_–SiO_2_, followed by the production of BTX from unsaturated aliphatic hydrocarbons via cracking, cyclization, hydrogen transfer, and dehydrogenation. Despite difficulties, poly(methylbenzene) (MB), including tri-MB and tetra-MB, and other aromatics were generated from the alkylation of light aromatics with olefins, favoring the production of BTX over Fe_2_O_3_–SiO_2_/modified-HZ catalysts. Meanwhile, CZA/modified-HZ catalysts followed the hydrocarbon-pool mechanism, where methanol first generated dimethyl ether DME at the CZA surface, followed by the conversion of DME into light olefins within zeolite channels. Hydrocarbon-pool species, mainly poly-MB, were then produced via oligomerization, cyclization, hydrogen transfer, and dehydrogenation of light olefins, leading to the formation of durene rather than BTX or tri-MB. Meanwhile, the selectivity of tetra-MB was lower over Fe-CZA/Ni-HZ due to the reduced formation of hydrocarbon-pool species, resulting from the suppression of dimethyl ether equilibrium by C_2_^=^–C_4_^=^.

**Figure 19:**
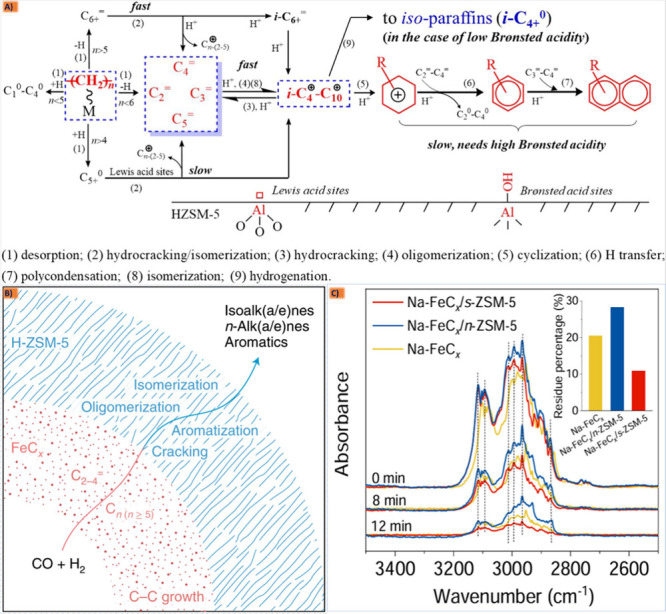
Symphony of HZSM-5 and its tandem reactions for light aromatic hydrocarbons by CO hydrogenation. (A) Reproduced with permission from ref (134). Copyright 2018 Elsevier. (B) Reproduced with permission from ref (281). Copyright 2024 Springer Nature. (C) In situ DRIFT spectra of different Fe/HZSM-5 catalysts. Reproduced with permission from ref (16). Copyright 2024 Nature Springer.

However, different kinds of reaction schemes have been demonstrated in recent studies, suggesting that the overall final product depends upon various catalytic features of both FTS and zeolite phases. These studies have delved deep into the various mechanisms and pathways underlying the formation of aromatic hydrocarbons from diverse hydrocarbon sources (Figure 19A–C), overall projecting a higher degree of aromatization for olefins compared to paraffinic intermediates.^263,282^ Notably, the cyclization of lower olefins has emerged as a prominent route, yielding efficient naphthene precursors that undergo subsequent aromatization via dehydrogenation processes. Xu et al.^149^ revealed in his report that over 95% aromatics in liquid hydrocarbons, mainly toluene, xylene, and 3C-branched alkylbenzenes, can be achieved by tuning different reaction parameters and catalyst combinations in the hybrid catalyst system of Fe-based/HZSM-5 zeolite while dictating the CO conversion, aromatic selectivity, and catalyst deactivation modes. It was further revealed that high Brønsted acidity in HZSM-5 favors aromatics, while reduced acidity due to coke deposition shifts selectivity to iso-paraffins. Figure 19A and B outlines the formation of aromatics and iso-paraffins from FTS hydrocarbon products over HZSM-5 zeolite. It can be illustrated that the HZSM-5 zeolite promotes aromatic formation from FTS hydrocarbons, particularly from small hydrocarbons (C_2_–C_5_), due to the space constraints within its channels. Depending on the architecture of the zeolite channel, these space constraints urge C_2_–C_5_ molecules to be preferentially adsorbed and undergo cycloaddition reactions, forming aromatic precursors (C_4_–C_10_), where the short chain hydrocarbons undergo cyclization and polymerization to form alkylbenzenes and subsequently alkyl-naphthalenes or aromatic coke. The balance between aromatization and isomerization, governed by the zeolite’s properties and reaction conditions, determines the overall aromatic content in the liquid C_5_^+^ phase. However, on HZSM-5 catalysts, the cracking of *n*-α-olefins triggers a selective increase in propene formation through a β-scission mechanism alongside the generation of ethene and butene fractions, while the shape selectivity effect exhibited by HZSM-5 zeolite tends to diminish the overall aromatization activity for long-chain paraffins. The activation of *n*-paraffins can yield carbenium ions and α-*n*-olefins, with the latter being desorbed directly from the surface due to reduced hydride transfer reactions within the small pores of HZSM-5.^283^ For the catalytic sites containing carbenium ion complexes and the reduced hydride transfer reactions in the small HZSM-5 pores, the α-*n*-olefins are desorbed directly from the surface rather than by exchanging the hydride ion with another molecule.^179^ A recent study by Wang et al.,^16^ investigating the promotional effect of zeolite in controlling the FTS reaction, revealed that Na-FeCx catalyst with s-ZSM-5 could exhibit better olefin tolerance even at lower temperatures, possibly due to improved desorption of olefin products from the catalyst surface, as supported by DRIFT spectra (Figure 19C). This accelerated desorption inhibits carbon chain growth, optimizing selectivity. In contrast, n-ZSM-5 delayed olefin desorption, leading to an excessive level of hydrogenation and alkane production. Sodium migration, which could affect catalytic activity, was ruled out, as recycled Na-FeC_*x*_ from Na-FeC_*x*_/s-ZSM-5 catalysts performed similarly to fresh Na-FeC_*x*_. Theoretical simulations revealed efficient olefin transfer from Na-FeC_*x*_ to zeolite micropores, enhancing desorption. Molecular dynamics simulations confirmed a shifted equilibrium between desorption and readsorption in the presence of zeolite, indicating its role in promoting ethene desorption from the Na-FeC_*x*_ surface given that the anticipated distribution of desired aromatic hydrocarbons in Fe-based STA processes hinges primarily on increasing the olefin-to-paraffin ratio (O/P).^284^

On the other hand, C_6_^+^ olefins desorbed from Fe carbides may undergo catalytic hydrocracking or isomerization over Brønsted acid sites to form C_3_–C_5_ olefins and iso-hydrocarbons. Meanwhile, heavier *n*-paraffins (C_5_^+^) may be hydrocracked or isomerized over Lewis acid sites, although these reactions are slow, as evidenced by the detection of *n*-paraffins in liquid phases. According to the widely accepted carbonium ion mechanism, C_2_–C_5_ olefins can be polymerized into iso-C_4_–C_10_ on Brønsted acid sites, followed by cyclization into alkylcyclohexanes within zeolite channels, where subsequent H transfers with C_2_–C_4_ olefins yield alkylbenzenes and C_2_–C_4_ paraffins. These alkylbenzenes can be further condensed into alkyl-naphthalenes and aromatic coke. Usually, iso-paraffins (C_4_^+^) are formed through the hydrogenation of iso-C_4_–C_10_ carbenium ions with the zeolites having a high Si/Al ratio (low Brønsted acidity) or coke deposition occurs. The fractional analysis of the liquid phase in C_5_^+^ hydrocarbons usually spans around the yield of aromatics and iso-paraffins, where the selectivity toward the formation of aromatics and their content in the liquid C_5_^+^ phase is determined by the competition between aromatization and isomerization reactions, which varies depending on the properties of different HZSM-5 zeolites and reaction conditions. On the other hand, the integration of HZSM-5 zeolite with the Fe-based catalyst may also lead to a significant increase in lower paraffins such as CH_4_ compared toMTS catalysts. CH_4_ primarily forms on the Fe-based catalyst during FTS through the hydrogenation of metal-CH_2_ groups, where the strong acidity of HZSM-5 facilitates the cracking of hydrocarbons into short-chain hydrocarbons, including CH_4_. On the other hand, zeolite releases hydrogen during aromatization, raising the local partial pressure of H_2_ over FeC_*x*_ sites and thus enhancing the hydrogenation of CH_2_ groups into CH_4_. Alkali additives (like Na or K) are added to the FTS catalysts that would counteract the reduction in CH_4_ selectivity via the electron-donor effect by promoting olefin formation and chain propagation and nullifying the effect of zeolite opposing role, enhancing the hydrogenation phenomenon. However, the interplay of different reaction parameters profoundly influences product distribution across catalytic sites within both the Fe phase and the zeolite phase, leveraging kinetic and thermodynamic strategies, where the shifts underscore heightened intrazeolite diffusion limitations favoring the formation of heavier products.^263^ Meanwhile, by employing effective framework factors, these insights shed light on how the composition of hydrocarbon products confined within zeolitic micropores during alkene oligomerization dictates diffusion rates and selectivity.^269^ This occluded organic phase significantly shapes the outcomes of alkene oligomerization on medium-pore zeolites such as MFI, MEL, and TON. Understanding the convoluted interplay between kinetic aspects and intrazeolite transport constraints offers a pathway to effectively tailor rates and selectivity in alkene oligomerization and related molecular chain-growth reactions that can be achieved through strategic selection of the zeolite topology and optimization of the reaction conditions.

## 4 Impact of Catalytic/Process Parameters for the Targeted Development of BTX

**Figure 20:**
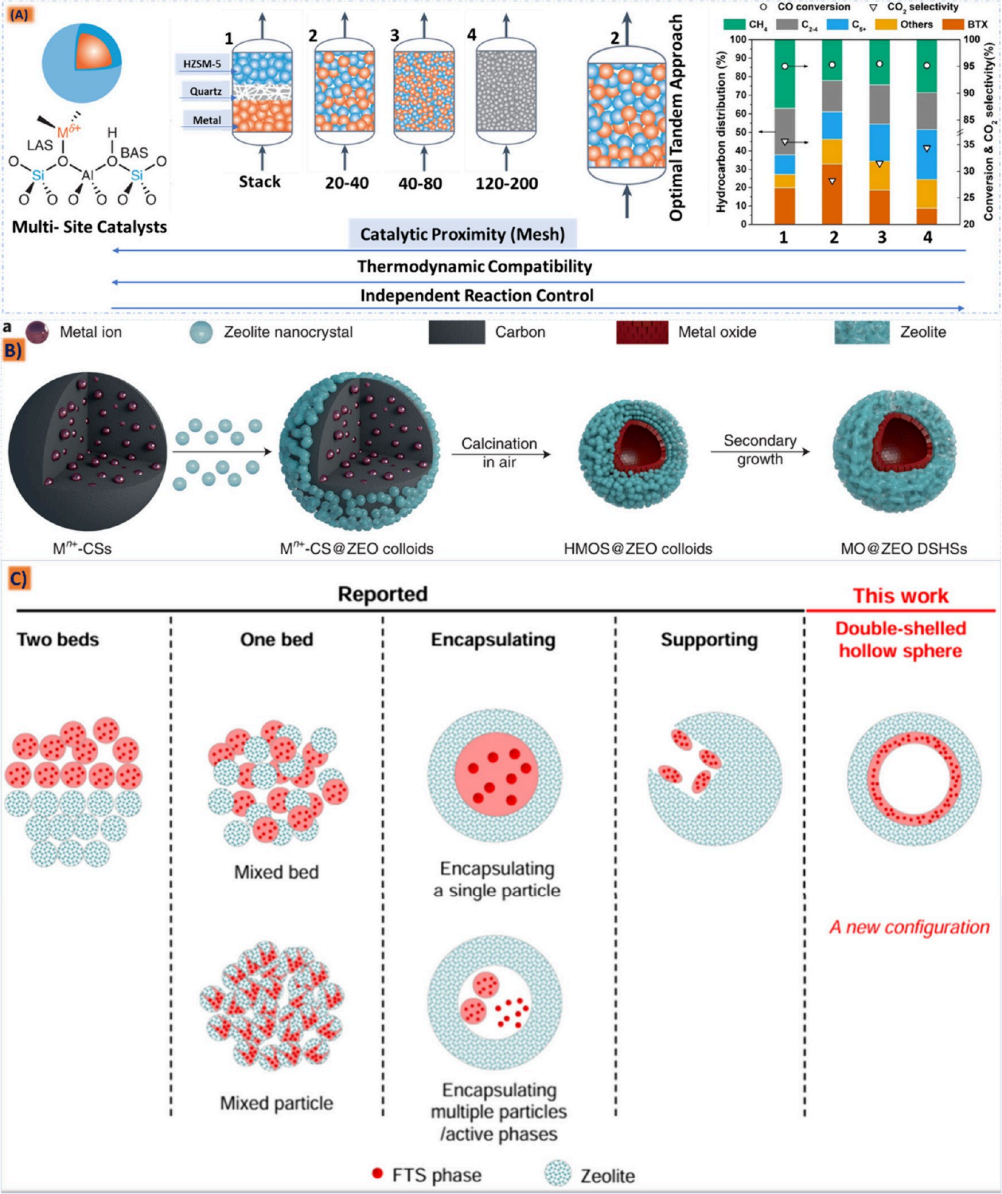
Different proximity analysis and synthesis schemes of single-bed, dual-bed, granular, or powder mixing of the Fe-based/HZSM-5 catalyst. (A) Reproduced with permission from refs (11) and (89). Copyright 2024 Nature Springer and 2020 American Chemical Society, respectively. (B, C) Reproduced with permission from ref (281). Copyright 2024 Nature Springer.

It has always been demonstrated in various studies that meticulous design and the regulation of catalyst proximity are imperative to optimize the efficiency and stability of tandem catalytic systems for syngas conversion to aromatic hydrocarbons (Figure 20A). Similarly, a straightforward approach of creating double-shelled, metal oxide@zeolite hollow spheres (MO@ZEO DSHSs) with adjustable structural parameters and compositions by assembling zeolite nanocrystals onto the surface of carbon spheres containing metal ions, followed by calcination and zeolite growth, contributed to the advancement of the rational synthesis and exploration of hierarchically hollow, core–shell multifunctional catalysts^281^ (Figure 20B and C). Multiple characterization approaches revealing the step-by-step formation mechanism of these materials illustrated that Fe_2_O_3_@H-ZSM-5 DSHSs catalyst could exemplify superior performance of 79% CO conversion and 64% C_5_–C_11_ hydrocarbon selectivity with the enhanced fractions of isohydrocarbons and aromatics resulting from the oligomerization of linear alkenes catalyzed by the acidic zeolite component followed by isomerization, aromatization, and cracking, which notably competed with the upper limit of the ASF distribution. On the other hand, various studies have also explored the developmental and technological aspects of specialized yolk/core–shell nanoreactors (YCSNs) suitable across various gas- and liquid-phase hydrogenation reactions, while discussing the catalyst structures, catalytic performance, structure-performance relationships, and reaction mechanisms. It has been demonstrated that the catalytic performance of the heterogeneous catalysts can be enhanced by employing YCSNs with unique architectures and beneficial properties in nanoreactor design.^262,285^ Spatial arrangement or close proximity between catalysts streamlines the transfer of intermediates, boosts reaction rates, and mitigates unwanted side reactions. This setup fosters synergistic interactions among diverse active sites, facilitating the sequential conversion of intermediates into desired products without intermediary separation.^42,266,268,281^ Nonetheless, achieving an ideal catalyst proximity presents challenges. The haphazard distribution of catalyst components may induce nondirectional diffusion, resulting in diminished catalytic efficacy. Moreover, maintaining stable proximity over prolonged reaction periods can be arduous, as catalysts may undergo deactivation or structural alterations. On the contrary, adverse effects might occur when a product of the first catalyst inhibits the second or the catalytic material reacts with each other, causing deactivation. Here, carbon deposition or promoter migration may block the microchannels in the zeolite, leading to reduced diffusion of syngas between the shell and the core. In particular, at the nanoscale, such as in powder mixing (PM) 120–200 mesh, rapid catalyst deactivation often occurs due to metal migration, which neutralizes zeolite acid sites and alters the metal surface, thus leading to earlier deactivation.^31,89,286,287^ Therefore, a trade-off between such effects will result in an optimal distance between the active sites that needs to be adjusted by the catalyst design scheme, especially for a FTS-based aromatic synthesis route.^81,89,140,284,286,288^

**Figure 21:**
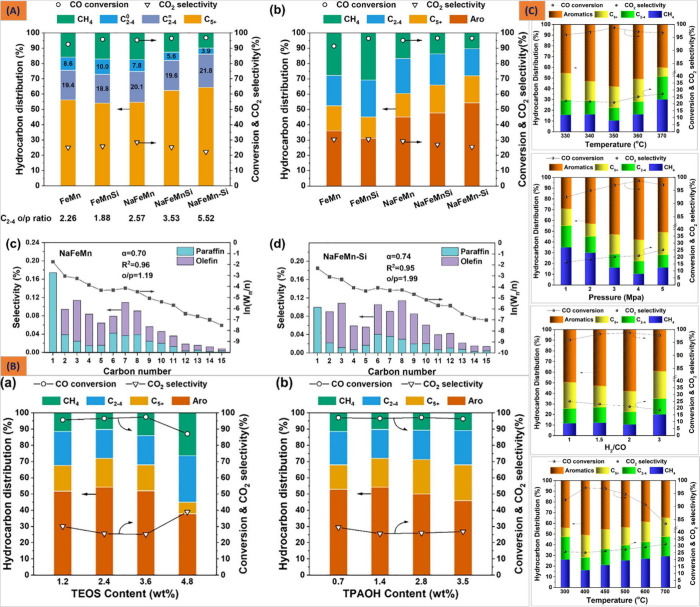
Effect of different catalytic and reaction parameters, such as temperatures, pressures, TEOS/syngas ratios, and space velocities over Fe-based/HZSM-5 catalysts in syngas conversion to aromatic hydrocarbons. (A, B) Reproduced with permission from ref (138). Copyright 2022 Elsevier. (C) Reproduced with permission from ref (136). Copyright 2022 Elsevier.

The catalytic prowess of the Fe-based/HZSM-5 catalyst could spark a deeper exploration into the optimal conditions for its performance across various reaction environments,^36,61,62,67,81,89,136,138−140,200^ venturing into different process or catalytic parameters for pretreatment or reaction temperature, pressure, space velocity, H_2_/CO ratio, and TEOS/TPAOH ratio to uncover a myriad of insights as per our recent studies (Figure 21). Generally, it has been demonstrated that at lower temperatures the sluggish progression of CO conversion gradually accelerates before plateauing, while equilibrium peaks at 90–95% for temperatures around 320–350 °C. Meanwhile, the FT polymerization reaction, known for its exothermic nature, benefits from elevated temperatures, aiding in the breakdown of C–C linkages. This temperature boost prompts a shift in intermediate products toward lighter olefins, courtesy of enhanced desorption probabilities on the metal catalyst surface. However, to catalyze the aromatization of these olefins on the zeolite surface, higher temperatures are required, leading to an enriched BTX fraction in the liquid phase. Intriguingly, as temperatures increase, CH_4_ selectivity experiences a noticeable uptick as well, since a careful tuning of temperature around 300–350 °C for Fe-based catalyst could emerge as the optimal STA reaction temperature. This choice is pivotal, as it dictates the diverse catalytic behavior of hydrocarbon growth on the catalytic surface, profoundly impacting the overall distribution of aromatics. Similarly, increasing the reaction pressure from 10 to 40 bar, guided by Le Chatelier’s principle, sparks a positive transformation in CO conversion and the preference for STA products. This pressure uptick coincides with a drop in CH_4_ levels and a surge in liquid-phase offerings, boasting an array of C_5_^+^ aliphatic compounds and enticing aromatics. The intensified pressure nurtures the tangled processes of polymerization and cyclization among intermediate olefins, culminating in an elevated preference for aromatic hydrocarbons. Furthermore, simultaneous pressurization amplifies the likelihood of chain growth while curtailing the formation of CH_4_ and CO_2_. Yet, excessive pressure proves counterproductive for aromatic hydrocarbon synthesis. Thus, following meticulous scrutiny, 40 bar emerges as the goldilocks pressure for optimal performance. Scaling further to 50 bar, dominance tilts toward C_5_^+^ aliphatic compounds in the liquid phase, accompanied by a notable spike in CO_2_ selectivity. This hints at the potential promotion of hydrogenation and isomerization of olefins at higher pressures, albeit at the expense of their transformation into aromatics over HZSM-5. When it comes to the promoters’ interaction in the Fe-based catalysts, obviously CH_4_ and CO_2_ selectivity tends to be lower around active sites, attributed to the strong CO adsorption facilitated by those added metal components. However, tinkering with the H_2_/CO ratio can cause a significant shift due to the twisted interplay between FT synthesis and the WGS reaction. For instance, at a 1:1 H_2_/CO molar ratio, a decrease in CH_4_ selectivity and an increase in CO_2_ levels would occur, with significant WGS activity. Contrarily, increasing the ratio may restrain WGS activity through Le Chatelier’s principle while lowering CO_2_ selectivity and promoting carbon atoms. Conversely, an increase in the H_2_/CO ratio increases the overall selectivity of aromatics; however, intensively elevating this ratio could also lead to a higher partial pressure of H_2_, favoring excessive surface hydrogenation and subsequently increasing CH_4_ formation while limiting overall aromatics selectivity. CO_2_ is a typical byproduct in Fe-based FTS, with its formation potentially linked to the WGS reaction, since in ideal cases of syngas as a reactant molecule low CO_2_ selectivity is desirable for maintaining high H_2_ pressure and suppressing the overall WGS reaction. Additionally, altering the gas hourly space velocity (GHSV) impacts reactant and product contact times, thus influencing conversion and chain growth. While a higher GHSV decreases the contact time, resulting in incomplete transformation of olefins into aromatics, a lower GHSV increases the residence time on active sites, accelerating conversion and enhancing product selectivity. However, excessive contact time may lead to undesired side reactions, diminishing overall aromatics selectivity. Consequently, an average value of 1000–2000 h^–1^ is deemed optimal for space velocity.

**Figure 22:**
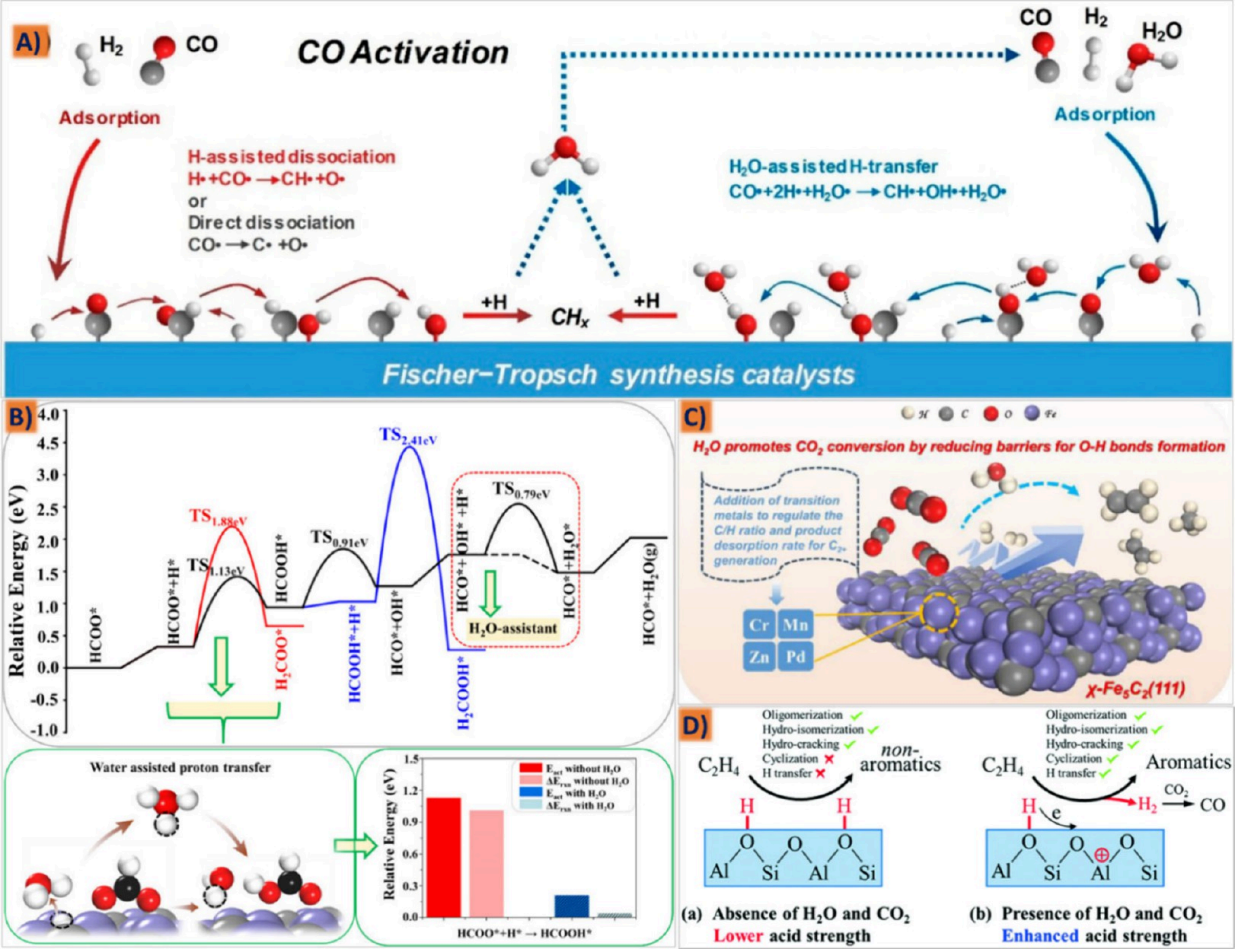
Effect of H_2_O on the catalytic activity of the FTS reaction. (A) Reproduced with permission from ref (289). Copyright 2024 American Chemical Society. (B, C) Reproduced with permission from ref (193). Copyright 2023 American Chemical Society. (D) Reproduced with permission from ref (137). Copyright 2019 Royal Society of Chemistry.

Yet, amidst this crucial endeavor, water, typically seen as a competitor to these molecules in catalytic processes, unveils a dual role: a hindrance and a catalyst. A detailed report on the effect of water composed of recent strides unraveled the mechanistic insights behind water’s influence on C_1_ molecule conversion, offering a profound exploration into its roles as a promoter, a conduit for proton transfer, an oxidant, and a direct source of elemental hydrogen or oxygen^289^ (Figure 22A). H_2_O, a ubiquitous byproduct in processes like the direct hydrogenation of CO_2_ to methanol and the reverse water–gas shift reaction, holds a complex sway over methanol synthesis. Sometimes intentionally introduced as a promoter, its impact on the process is a blend of blessings and hurdles, contingent upon the catalyst nature and the water partial pressure. While conventionally the methanol production pathway involves adsorbed hydrogen, recent insights suggest the involvement of co-adsorbates derived from surface oxygen or water-related species, which is crucial for catalyzing methanol over certain catalysts, such as Cu-based ones. The presence of water can dynamically alter reaction pathways, facilitating the formation of intermediates pivotal for methanol synthesis. Additionally, in the realm of Fe-based FTS catalysts, water’s role becomes even more periphrastic due to the concurrent water–gas shift reaction. Its addition to syngas can modulate CO and H_2_ partial pressures, influencing FTS rates, albeit with nuances influenced by factors like reactor type, water quantity, and H_2_/CO ratio. The interplay between water and catalysts underscores the complex choreography of factors shaping industrial catalysis, revealing a rich tapestry of possibilities and challenges. Therefore, in the captivating world of chemical reactions, the interplay between each parameter and reactants unfolds a mesmerizing adaptability of multiple factors. Zhang et al.,^193^ in an attempt to define the mechanism behind hydrocarbon formation from CO_2_ hydrogenation on the χ-Fe_5_C_2_ (111) surface by DFT calculations, determined that the presence of H_2_O* species on the χ-Fe_5_C_2_ (111) surface notably lowered barriers for O–H bond formation, thereby modifying reaction routes and enhancing CO_2_ conversion rates (Figure 22B and C). Similarly, incorporating a secondary transition metal (e.g., Mn, Zn, Cr, or Pd) into χ-Fe_5_C_2_ (111) was found to adjust the surface C/H ratio and influence product desorption rates, thus offering a means to tailor the product selectivity. In another study by Wang et al.^137^ taking the model reaction with C_2_H_4_ over HZSM-5, for instance, at a gentle value of 320 °C, a smooth progression was witnessed in oligomerization and hydrocracking-isomerization reactions (Figure 22D). Yet, as the temperature climbed to 350 and 450 °C, a dramatic surge in aromatics formation took center stage. An intriguing result was the positive impact of introducing H_2_O or CO_2_ alongside C_2_H_4_, akin to raising the temperature, hinting at an efficient H transfer mechanism fueled by H_2_O and CO_2_. While in their earlier study the authors found that a higher density of acid sites could catalyze aromatics formation by facilitating C–C bond breakage, when H_2_O or CO_2_ enters the mix, the acid sites within HZSM-5 zeolite strengthen, potentially accelerating the conversion of hydrocarbons into aromatics. For the HZSM-5 zeolite with a low Si/Al ratio, the mechanism was even more fascinating. Partial hydrolysis of paired Al sites created Al^+^ sites, boosting the neighboring Brønsted acid site’s potency. And then there is CO_2_, perhaps the unsung hero in this chemical ballet. It might play a pivotal role in driving H transfer, enhancing cyclization and dehydrogenation for aromatic formation and acting as a “hydrogen (H^•^) acceptor”. A notable surge in CO formation and aromatic content was produced by introducing H_2_ into the mix with CO_2_, possibly fueled by the reaction of CO_2_ with H_2_. However, intriguingly, no aromatics were formed when C_2_H_4_ reacted with CO/H_2_ over HZSM-5 at 320 °C, emphasizing the complex nature of various parameters dictating the product streams. Therefore, it could be stated that in this labyrinthine chemical narrative, every element and every reaction adds depth and complexity, elucidating the multifaceted tapestry of molecular interactions that shape our understanding of catalytic processes.

## 5 Challenges and Opportunities

In the quest for sustainable and efficient chemical processes, the synthesis of aromatic hydrocarbons from syngas via tandem catalysis has emerged as a promising frontier in chemical engineering. As we navigate through the complexities of this multifaceted reaction network, we encounter both challenges and opportunities that are beckoning innovative solutions and advancements.

**Figure 23:**
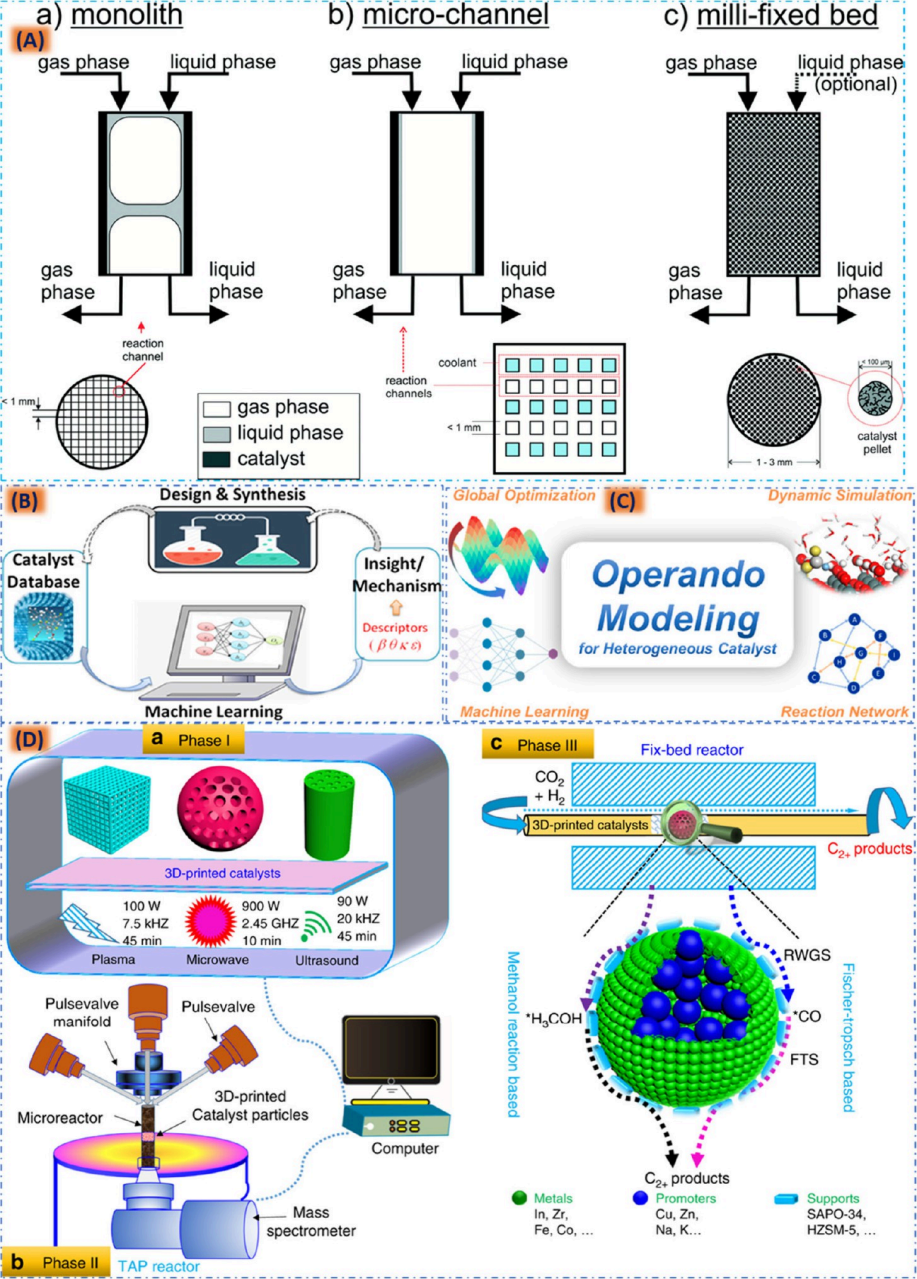
Use of compact reactor designs and process intensifications for the tandem approach via machine learning and artificial intelligence, (A) Reproduced with permission from ref (293). Copyright 2015 Royal Society of Chemistry. (B) Reproduced with permission from ref (290). Copyright 2020 American Chemical Society. (C) Reproduced with permission from ref (291). Copyright 2021 American Chemical Society. (D) Reproduced with permission from ref (263). Copyright 2022 American Chemical Society.

Optimizing the efficiency and selectivity of tandem catalyst systems stands as a formidable challenge, requiring precise control over reaction pathways and intermediate species. Achieving high yields of aromatic hydrocarbons requires precise control over the reaction pathways and intermediate species, which can be influenced by factors such as catalyst composition, proximity of active sites, and reaction conditions. Yet, within these challenges lie opportunities for breakthroughs in catalyst design and synthesis techniques. By tailoring catalyst structures and compositions, we can enhance catalytic activity and selectivity, paving the way for economically viable and environmentally sustainable processes. Ensuring optimal catalyst proximity is crucial for facilitating the efficient transfer of intermediates between different active sites and minimizing undesirable side reactions. Additionally, maintaining stable catalyst proximity over extended reaction times can be challenging due to catalyst deactivation or structural changes. Furthermore, the complex nature of the reaction network involved in syngas conversion presents another challenge. The production of aromatic hydrocarbons involves multiple sequential reactions, including hydrogenation, oligomerization, cyclization, and dehydrogenation, each with its own set of kinetics and thermodynamics. Balancing these reactions to favor the desired aromatic products while minimizing the formation of undesired byproducts requires a thorough understanding of the underlying mechanisms and careful optimization of the reaction conditions. The intricacies of syngas conversion demand a comprehensive understanding of the reaction kinetics and thermodynamics. Through ongoing research into fundamental mechanisms, compact reactor designs, and the advent of computational modeling, machine learning, and operando characterization tools, we gain invaluable insights into catalytic behavior (Figure 23A–D).^196,290−293^ Armed with this knowledge, we can devise novel strategies to improve the catalytic performance and overcome existing limitations. Additionally, overcoming hurdles like sintering, metal–support interactions, and metal–reactant/solvent interactions will be pivotal in attaining optimal performance for specific reaction pathways. Hence, the strategy of process intensification involves amalgamating Fischer–Tropsch synthesis (FTS) and hydrocracking catalysts to streamline numerous stages within a continuous framework with the goal of boosting atom economy. This approach emphasizes the efficient incorporation of heat and the effective utilization of residual heat streams within the intensified process to improve the overall energy management.

Ongoing research is also focused on developing new catalytic systems including compact reactor designs (microreactors or structured reactors) and process intensifications, which can provide better control over reactant ratios and residence times, improving the overall selectivity of the process and optimizing reaction conditions for a tandem approach. The laminar fluid flow within microchannels would enable precise process control, rapid mixing, and short residence time, leading to faster reactions with higher yields and productivity compared to conventional large reactors. The objective would be to accelerate the industrialization of this technology to eliminate the radial diffusion limitations and make it effective for converting syngas into ethylene or propylene monomers and expanding the product stream to the wide range of fundamental chemicals and fuels, including cycloparaffins, aromatics (BTX), and synthetic aviation fuels. In summary, although the production of aromatic hydrocarbons from syngas by tandem catalysis presents significant challenges, it also offers numerous opportunities for innovation and advancement. By addressing these challenges and capitalizing on emerging opportunities, researchers can contribute to the development of more efficient, sustainable, and economically viable processes to produce aromatic hydrocarbons from syngas. In a tandem catalyst, the metal oxide phase dictates the type of intermediate and the rate of formation in tandem catalysis, while the molecular sieve’s size, structure, and environmental properties determine the final product. Therefore, by modulating the oxides and molecular sieves, such as their composition and structure, and optimizing the coupling between the two, precise control over product composition can be achieved. It should be ensured that selected catalysts are compatible with the overall tandem setup, where the consideration of factors such as the physical form of the catalyst (e.g., supported nanoparticles, zeolites, or composite materials) and their integration manner within the catalytic system is much more important. Thus , process intensification, exemplified by the integration of Fischer–Tropsch synthesis and hydrocracking catalysts, holds the promise of streamlining multiple stages within a continuous framework. Here, adopting high pressure (2–4 MPa) for oligomerization or cyclization of intermediates, elevated temperature (300–350 °C) for increasing cracking reactions, an appropriate syngas ratio (1–2), and adequate space velocity (1500–4000 mL·h^–1^·g^–1^) or residence time is essential in a multistep tandem catalysis reaction for biofuel production. By harnessing various process parameters (pressure, temperature, and appropriate syngas ratios), we aim to drive specific reactions while minimizing unwanted byproducts. This strategy not only enhances the atom economy but also optimizes energy management, contributing to the sustainability of the process. The current production of aromatic hydrocarbons from syngas by tandem catalysis also presents several opportunities for advancement. Advances in catalyst design and synthesis techniques offer the potential to develop highly efficient and selective catalyst systems tailored specifically for syngas conversion. By engineering catalysts with optimized structures and compositions, researchers can enhance catalytic activity, selectivity, and stability, leading to improved process economics and environmental sustainability. Moreover, ongoing research into the fundamental mechanisms of tandem catalysis provides valuable insights into the factors influencing the reaction kinetics and selectivity. By elucidating the underlying principles governing catalytic behavior, researchers can identify new strategies for enhancing the catalytic performance and overcoming existing limitations.

## 6 Conclusion

In the ever-evolving landscape of chemical synthesis, the journey toward sustainability has never been more crucial. As we stand at the crossroads of industrial growth and environmental stewardship, the synthesis of aromatic hydrocarbons from syngas has emerged as a beacon of innovation and responsibility. The narrative of ingenuity woven through nonpetroleum routes not only presents a compelling story of sustainability but also reflects our commitment to shaping a future where chemistry and environmental consciousness walk hand in hand. By embracing sustainable practices, we not only address pressing environmental concerns but also fortify the resilience and competitiveness of the industry in the long term. As we navigate toward greener horizons, transformative technologies for catalyst development hold the promise of revolutionizing the landscape of aromatic hydrocarbon synthesis. From pioneering material manufacturing to AI-guided catalyst evaluation, these advancements offer a glimpse into a future where sustainability is not just a goal but also a guiding principle. In summary, the soaring demand for aromatic hydrocarbons underscores the urgency of embracing sustainable synthesis methods. Through the integration of biomass-derived feedstocks, catalytic innovations for Fe-based/HZSM-5 systems, and a commitment to evolving regulatory standards, we pave the way for a more sustainable chemical industry that prioritizes environmental responsibility, resource conservation, and the principles of the circular economy. Together, let us embark on this journey toward a brighter, greener future.
